# The relationship between circulating and tissue biomarkers and OA-related pain: A systematic literature review

**DOI:** 10.1016/j.ocarto.2025.100684

**Published:** 2025-09-17

**Authors:** Sylvain Mathieu, Liisa Kuhi, Marie Binvignat, Philip G. Conaghan, Niels Eijkelkamp, Yves Henrotin, Eva Kosek, Ali Mobasheri, Hans-George Schaible, Kalle Kisand, Jérémie Sellam, GO-PAIN working group, Françoise Alliot-Launois, Nadine Attal, Francis Berenbaum, Marie Binvignat, Philip Conaghan, Alice Courties, Niels Eijkelkamp, Camille Fauchon, Rinie Geenen, Ida K. Haugen, Yves Henrotin, Kalle Kisand, Margreet Kloppenburg, Eva Kosek, Liisa Kuhi, Sylvain Mathieu, Céline Mathy, Ali Mobasheri, Stanislas Moumbe Talla, Patrick Omoumi, Serge Perrot, Roland Peyron, Simo Saarakkala, Alain Saraux, Hans-Georg Schaible, Jérémie Sellam

**Affiliations:** a Université Clermont Auvergne, Service de Rhumatologie, CHU Clermont-Ferrand, INSERM, Neuro-Dol, 63000, Clermont-Ferrand, France; b Central Laboratory, East Tallinn Central Hospital, Tallinn, Estonia; c Department of Rheumatology, Saint-Antoine Hospital, Assistance Publique-Hôpitaux de Paris (AP-HP), Paris, France; d Leeds Institute of Rheumatic and Musculoskeletal Medicine, University of Leeds, NIHR Leeds Biomedical Research Centre, Leeds Teaching Hospitals NHS Trust, Leeds, UK; e Center for Translational Immunology, University Medical Center Utrecht, Utrecht University, Utrecht, the Netherlands; f MusculoSKeletal Innovative Research Lab, CHU Sart-Tilman, Liège, Belgium; g Physical Therapy and Rehabilitation Department, Princess Paola Hospital, Vivalia, Marche-en-Famenne, Belgium; h Department of Clinical Neuroscience, Karolinska Institutet, Stockholm and Department of Surgical Sciences, Uppsala University, Uppsala, Sweden; i Research Unit of Health Sciences and Technology, Faculty of Medicine, University of Oulu, Oulu, Finland; j State Research Institute Centre for Innovative Medicine, Vilnius, Lithuania; k Department of Joint Surgery, First Affiliated Hospital of Sun Yat-sen University, Guangzhou, China; l Institute of Physiology 1/Neurophysiology, Jena University Hospital, Friedrich-Schiller-University, D-07740, Jena, Germany; m Department of Internal Medicine, Institute of Clinical Medicine, University of Tartu, Estonia; n Sorbonne Université Department of Rheumatology, Saint-Antoine Hospital, Assistance Publique–Hôpitaux de Paris (AP-HP), Centre de Recherche Saint-Antoine (CRSA), Inserm UMRS-938, Paris, France

**Keywords:** Osteoarthritis, Pain, Biomarkers, Systematic review

## Abstract

**Objective:**

This study aimed to provide an overview of the relationship between osteoarthritis (OA) pain and various fluid biomarkers by conducting a systematic literature review (SLR), to help the development of OA-related pain endotyping.

**Method:**

An SLR was conducted, using the PubMed, Embase, Scopus, Web of Science and the Cochrane Library databases, up to December 2024. Pain measures (VAS, WOMAC, HOOS/KOOS, AUSCAN, PainDETECT and Pain Pressure Threshold) were analysed for their association with circulating biomarkers in blood, urine, synovial and cerebrospinal fluids or tissue and genetic biomarkers. Biomarkers were categorised as “associated” depending on statistical significance and further subcategorised as “consistently associated”, “uncertainly associated” or “not associated” based on the quality of evidence determined by the number of studies, sample size and the strength of correlation.

**Results:**

The five databases yielded 30,088 citations, of which 263 relevant papers were selected. Total cholesterol in the blood was the only biomarker consistently associated with pain. Among blood biomarkers, CRP, hsCRP and IL-17 showed suggestive but inconsistent associations with OA-related pain. In synovial fluid, IL-17, C2C and VEGF were consistently associated with increasing pain intensity, based on multiple concordant studies. In cerebrospinal fluid, CX3CL1 and Flt-1 were consistently associated with pain, displaying a negative correlation.

**Conclusion:**

This SLR identified no relevant biomarkers in different body fluids that were associated with OA-related pain. Further investigation of CRP and IL-17 is required to achieve greater consistency across studies.

**PROSPERO:**

CRD42024550244.

## 1 Introduction

Osteoarthritis (OA) is one the most prevalent degenerative joint diseases, affecting millions globally and serving as a leading cause of chronic pain, which significantly reduces the quality of life of people with OA [1]. Despite its widespread occurrence, early detection and effective treatment of OA remain challenging. OA-related pain is complex and involves not only structural changes in the joints but also chemical and neurophysiological factors [[2], [3], [4]]. The immune system is known to contribute to pain [5]. Within the OA population several phenotypes can be defined [[8], [9], [10]]. In addition, several pain subtypes coexist, distinguished by nociceptive or neuropathic components [6,7]. Identifying these phenotypes through synovial fluid (SF) biomarkers may help identify and tailor treatment options by targeting inflammatory pathways, similar to approaches used in patients with rheumatoid arthritis [11].

Biomarkers, which serve as measurable biological indicators, have emerged as valuable tools in the diagnosis, prognosis, and management of OA. They also provide insights into pathological biological processes such as inflammation, cartilage degradation, and nerve sensitization, all contributing to the pain experience in OA [[12], [13], [14], [15], [16], [17]]. We adopted the updated definition of a biomarker reported by the FDA-NIH Biomarker Working Group. A biomarker is a measurable characteristic that indicates a normal biological process or response to an exposure or intervention. Here, we have focused on circulating biomarkers in relation to OA-related pain. These biomarkers can be cells, proteins or mediators in biological fluids. We have also included the synovial biopsy and genetic findings [18].

A wide range of studies have linked various biomarkers reflecting various biological processes such as inflammation, cartilage degradation or sensitization, to be associated with OA and OA-related pain [[18], [19], [20], [21], [22], [23], [24], [25]]. A considerable number of studies have assessed the association of biomarkers in blood, urine, SF or cerebrospinal fluid (CSF) biomarkers to OA-related pain [18,19]. However, diverse methodologies and occasional conflicting results have made it difficult to draw definitive conclusions. There are also studies on DNA single nuclear polymorphism and synovial tissue analysis in relation to OA pain. Identifying specific biomarkers linked to pain can help researchers and clinicians develop more effective pain management strategies, improve patient outcomes, and tailor treatments to meet individual needs. A precise understanding of the biomarkers profile in OA patients could refine the definition of OA-related pain and lead to a more efficient analgesic treatments [26].

Therefore, to improve the assessment and management of OA-related pain, this study aimed to delineate the current state-of-the-art knowledge on OA-related pain phenotyping through a well-established systematic literature analysis and explore the relationship between OA pain and various fluid biomarkers.

## 2 Materials and methods

The research protocol was registered on PROSPERO (CRD42024550244).

### 2.1 Literature search

We searched 5 databases: MEDLINE via PubMed, EMBASE, Scopus, Web of Science, and the Cochrane library to identify all reports of interest concerning the relation between biomarkers and OA-related pain from inception until 2024/12/31. The search strategy comprised terms relating to three key concepts: ‘osteoarthritis’, ‘pain’ and ‘biomarkers’. For each concept, key words and Medical Subject Heading terms were combined. The search equations can be found in the supplementary file S1.

### 2.2 Eligibility criteria

Included studies were case-controls, cross-sectional or longitudinal studies whatever the joint location (knee, hip, hand, or other). It was mandatory that the data pertain to pain and to its relationship with biomarkers. Circulating biomarkers could be measured in blood, urine, SF or CSF and tissue biomarkers could concern synovial, muscle or fat pad tissue. Excluded were commentaries, protocols, editorials, case reports, articles concerning children, and studies with no full-text available. No comparator (e.g. a control group) was necessary in our study. Our search was restricted to articles published in English or French. Reviews were excluded, but congress abstracts from the American College of Rheumatology, European League Against Rheumatism (EULAR) and the Osteoarthritis Research Society International congresses in the past two years were allowed. We also excluded the studies that assessed biomarkers after surgery, especially arthroplasty.

### 2.3 Study selection

Records were imported into Rayyan software [27] and duplicates were removed. Two investigators (SM and LK) initially selected potentially relevant articles by titles, keywords, and abstracts, followed by full text review. Two additional authors (KK and JS) oversaw the selection process, resolved discrepancies between the investigators, confirmed the inclusion and exclusion studies, and ensured no relevant studies were overlooked. Articles were selected after consensus between all investigators. The methodological quality of each included study was assessed using the Newcastle-Ottawa Scale forms for case-control, cross-sectional and cohort studies [28].

### 2.4 Data extraction

Two investigators (SM and LK) extracted all data from each study using a standardized data abstraction form. Extracted were the number of patients with OA included, the location of OA, the characteristics of patients (mean age, percentage of women) and the pain intensity measured by the different scales. We defined as relevant scales of pain: the visual analogic scale (VAS), the Western Ontario and McMaster University Hip Disability and Osteoarthritis Outcome Score (WOMAC pain) [29], the Knee Injury and Osteoarthritis Outcome Score (KOOS) [30], the Hip Injury and Osteoarthritis Outcome Score (HOOS) [31], the AUStralian CANadian pain index [32], the painDETECT [33], the pain pressure threshold (PPT) [34]. All circulating biomarkers measured in blood, urine, SF, and CSF biomarkers were included in case of results in the article concerning the relation between pain and biomarkers. For blood-based biomarkers, we distinguished between serum and plasma when this information was available in the original study. Genetic markers were also included, but analysed separately from circulating biomarkers. These results could be correlation or regression coefficients. To gain an overall view of the biomarkers related to OA-related pain and to highlight their complexity, we decided to explore the associations or correlations between all circulating biomarkers, genetic or synovial biopsy findings and all pain measures. To this end, we compiled both patient-reported outcome (PRO) measures (VAS, HOOS/KOOS, WOMAC and PainDETECT) and pain phenotype assessments (PPT).

### 2.5 Data analysis, synthesis and report

The relationship between pain measures and biomarkers was first defined as “associated” or “not associated”. In case of a significant correlation or regression (*p* ​< ​0.05), the biomarker was defined as “associated”. The further categorization of biomarkers as either “consistently associated”, “uncertainly associated” or “not associated” was based on the quality of available evidence. We used “consistently” when all the studies produced concordant results, meaning that they produced coherent results that pointed in the same direction. However, this classification reflects the consistency of evidence, not the magnitude or strength of the correlation. Some biomarkers with “consistent associations” may still exhibit low correlation coefficients (e.g., r ​∼ ​0.2–0.25), which limits their clinical significance. “Uncertainly” was used if an association between OA pain and a biomarker was reported in only one study or if the direction of the association (positive or negative) differed between studies. A positive (or negative, respectively) association was identified when the correlation coefficient or regression coefficient was positive (or negative, respectively) for the association between biomarkers and VAS pain or WOMAC pain, and negative (or positive, respectively) for the association between biomarkers and HOOS or KOOS. For HOOS and KOOS, the scale is inverted in relation to the VAS and WOMAC scales; the lower the score, the more intense the pain. A score of 0 in HOOS and KOOS indicates the worst possible symptoms, while a score of 100 indicates no symptoms. The choice between “uncertainly associated” and “uncertainly not associated” considered the number of studies, the samples size of patients within each study, and the strength of correlation. The relation was defined as strong, moderate, weak and very weak according to the “r” correlation coefficient, i.e. respectively >0.7, between 0.4 and 0.7, between 0.2 and 0.4, and <0.2. Where there are several studies on the same biomarker with different strengths of association, a consensus group (SM, LK, KK, JS) defined the final decision on the strength of correlation. For example, the association was strong in the first study (correlation>0.7) and moderate in the second (correlation between 0.4 and 0.7). After analysing these two studies, the consensus group concluded that the biomarker is consistently associated with pain, and that this association is positive. Although we did not take adjustment factors into account when making our decision, we noted whether the results had been adjusted for factors such as age and BMI. A sample size higher than altogether 100 patients included in the studies for each biomarker was considered as relevant, i.e. high enough to make the results strong. The categorization of each biomarker was first discussed between four researchers (SM, LK, KK, JS) and then with all the co-authors until consensus was reached. Similarly, groups reflecting the main pathophysiological processes of OA were formed, and each biomarker was assigned to one of these groups: inflammation, pain sensitization, bone or cartilage degradation, formation, or metabolism.

When the data on the correlation coefficient (the estimate and the measure of uncertainty or effect size) were available in at least three studies for a biomarker, we performed a meta-analysis of the correlation coefficients. If heterogeneity was observed (p-value of the I^2^ test was <0.05), we used a random effects model; otherwise, we used a fixed effects model. The overall correlation coefficients, expressed as an effect size with a 95 ​% confidence interval, were obtained using Stata software version 14.2.

## 3 Results

### 3.1 Selected studies

**Fig. 1:**
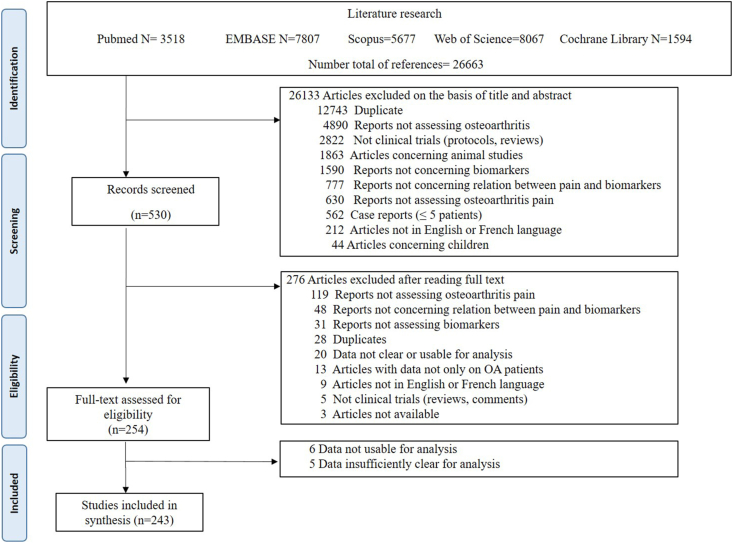
Flow chart depicting the identification of studies for inclusion in the review.

The five databases yielded a total of 30,088 citations with 263 studies meeting the eligibility criteria (Fig. 1 and Table S1).

### 3.2 Study and patients' characteristics

The 263 studies (*N* ​= ​number of studies) included 50,712 patients with OA (Table S1). For 28,762 patients (185 studies) data on sex were available, with 18,729 (68.5 ​% by metaproportion) being women. The weighted mean age of patients was 62.4 years, (193 studies; 30,886 patients) and the weighted mean BMI was 27.8 ​kg/m2 (152 studies; 27,286 patients).

The weighted mean pain intensity of all studies and measured using the VAS (0–10 range) was 5.1 across 62 studies involving 6285 OA patients. For the KOOS pain scale (0–100 range) the weighted mean was 80.1 from 16 studies with 2869 OA patients (Table S1). Due to differences and heterogeneity in measurement and results reporting across the 53 studies involving 9380 OA patients), the weighted mean WOMAC pain could not be calculated.

Of the 263 studies included, 68 were only conference abstracts (25.8 ​%) which were published in various journals, such as Osteoarthritis and Cartilage (*N* ​= ​28), Annals of the Rheumatic Diseases (*N* ​= ​9) or Arthritis and Rheumatology (*N* ​= ​5). Most of included studies involved knee OA (*N* ​= ​227), hip OA (*N* ​= ​24), hand OA (*N* ​= ​12), and other OA sites (*N* ​= ​6). The methodological quality concerned 195 studies (abstracts excluded). The most frequent bias was the lack of justification or calculation of the sample size (Table S6).

### 3.3 Relation between OA-related pain and fluid biomarkers

A total of 210 OA pain biomarkers were identified across 263 studies (Table S1). The significance and categorization of each biomarker, based on pathophysiological processes (inflammation, pain sensitization, bone or cartilage degradation, formation, or metabolism), are detailed in Table S2. Most pain-related studies focused on blood and SF biomarkers (Table S3). Blood (serum, plasma) biomarkers were studied in 182 studies, identifying 152 distinct biomarkers. Most blood biomarkers were measured in serum, while some (*n* ​= ​11, e.g. IL-1, IL-6, IL-8 and TNF) were measured in both serum and plasma (Table S3). In blood, total cholesterol was the only biomarker “consistently associated” with OA-related pain. Among blood biomarkers, CRP, hsCRP and IL-17 showed suggestive positive but inconsistent associations with OA-related pain. (Table 1 and Table S3). Forty-one and 20 other biomarkers were “uncertainly associated” with respectively a positive and negative association with pain. Conversely, 63 biomarkers were categorised as “consistently not associated”, while 29 others were “uncertainly not associated”.

**Table 1:** Association between pain and blood biomarkers.

	Association with pain			
	Consistently not associated	Uncertainly not associated	Uncertainly associated	Consistently associated
Blood biomarkers	ApoA1; ApoB; ARGS; b-endorphin; C1,2C; C2C; C10C; C3-α; C3f; calprotectin; Cath-K; CD14; CIR; classical monocyte CCR2; Coll 2-1; Coll2-1 NO2; cortisone; CPII; creatinine; CS846; CTX-I; CU/Zn SOD; ​CX3CL1; FABP2; Fib 3-2; GDNF; GM-CSF; HDL cholesterol; IGF-I; IL1-R; IL2; IL4-R; IL7; Int monocyte HLA-DR; LPS; MIF; MMP1; MMP13; MnSOD; Monocyte CD16; MUFA; myoglobin; NO; NTXI; omega3 PUFA; omega6 PUFA; omentin; PIIANP; PINP; PRO-C1; PRO-C2; PRO-C3; PRO-C4; resistin; S100A8/S100A9; SFA; TIMP; TGFbeta; Total FA; Total PUFA; visfatin; YKL-40 ; ZRP	Adiponectin; Adipsin; BDNF; C1M; C2M; C3M; chemerin; clusterin; COMP; cortisol; CRPM; CTX-II; ESR; FBG; HA; HbA1C; hTG; IL1; IL6; IL6-R; IL8; leptin; MCP1; MMP3; OC total; PIIINP; TNF alpha; uric acid; vitamin D	Positive association	
			13KODE; Acyl ornithine; Angiopoietin-2; ASAT; BMP2; B-GNLY; C3A; C4M; Carnosine; CGRP; CPK; CRP; Dopa; Dopamine-Ig; FGF-21; Fib 3-1; Fib 3-3; galectin3; ghrelin; hsCRP; IL2-R; IL15; IL17; IL23; IL25; IL38; Int monocyte CCR2; Insulin resistance; LBP; LC3A; LDH; LDL cholesterol; L/A or A/L; LTα; mTOR; osteopontin; PEA; PGE2; succinic acid; TNFalpha-R1; TNFalpha-R2; Treg; VEGF	Total cholesterol
			Negative association	
			C3-β; CCL2; CXCL9; CXCL10; Cystine; GLCAS; ICAM-1; IL4; IL5; IL10; IL12; IL13; IL21; IL22; INFgamma; IP10; miR300; PIICP; PDGF; T2GM	

Signification of the biomarkers is in Table S2.

In SF, 98 different biomarkers were analysed across 63 studies. IL17, C2C and vascular endothelial growth factor (VEGF) were identified as “consistently associated” with a positive correlation to OA pain. Thirty-seven biomarkers were “consistently not associated” including COMP, while seventeen were “uncertainly not associated”. Additionally, thirty-seven and nine biomarkers were “uncertainly associated” with pain with respectively a positive and negative association (Table 2).

**Table 2:** Association between pain and urinary, SF and CSF biomarkers.

	Association with pain			
	Consistently not associated	Uncertainly not associated	Uncertainly associated	Consistently associated
Urinary biomarkers	C1,2C; CIIM; Coll2-1 NO2; COMP; creatinine; CTX-I; NTXI	C2C; CTX-II	Positive association	
			Glc-Gal-PYD; TNFα	
			Negative association	

Synovial fluid biomarkers	ACRP-30; ADAMTS5; BDNF; BDNF/LNGFR; BK; C1M; C2M; C3-α; C3M; C10C; chemerin; COMP; CTX-I; CTX-II; IGF-1; IL1-R; IL23; LPS; lubricin; MCSF; MMP1 ​; MMP2; MMP8; NGF/LNGFR; NT-3; NT-3/LNGFR; osteoprotegerin; proNGF; proNGF/LNGFR ; RANK; RANKL/OPG; resistin; SP; TIMP; Treg; TrkA ; uric acid	Adiponectin; Adiponectin/leptin; ARGS; CGRP; HA; IL6; IL1; IL10; LBP; leptin/adiponectin; MMP3; NGF; NPY; omentin; osteopontin; PGE2; visfatin	Positive association	
			ATP; BMP2 ; bNGF; calprotectin; CCL20; CD163; CHOP; CIR; CPII; hsCRP; DKK1; eotaxin-I; GRP78; HMGB1; ICAM1; IL4; IL7; IL8; IL12; IL13; IFNgamma; leptin; LNGFR; MCP1; MDA; MIF; MMP9; MMP13; MMP14; NO; NGF/TrKA; uPA; SCGF-beta; TIMP-1; TNF alpha; VCAM-1; YKL-40	IL17; C2C; VEGF
			Negative association	
			C3-β; CD11c/CD206 ratio; GAG; ghrelin; hexadecenoic acid; PACAP; SOD; TAC; TSG-6	
CSF biomarkers	BDNF; GNDF; ICAM-1; IL1; IL10; IL15; Leptin; MCP1; PIGF; VCAM-1; TNFalpha; TNFalpha-R1	IL6; IL8; IP-10 ; TGFbeta; VEGF	Positive association	
			Aβ40; IL6-R; SPLI ; TNFalpha-R2	
			Negative association	
			CSF-1; HGF; IL1-R; LIF-R; SCF; VEGFA; TWEAK	CX3CL1; Flt-1

Signification of the biomarkers is in Table S2.

In urine, eleven biomarkers were studied with none “consistently associated”. CTX-II and C2C were “uncertainly not associated”, whereas Glc-Gal-PYD and TNF were “uncertainly associated”.

In CSF, biomarkers were studied in eleven studies with twelve “uncertainly associated”, including four with a positive association (e.g., Aβ40, IL6-R, SPLI and TNFalpha-R2) and the other ones with an inverse relationship. Flt-1 and CX3CL1 were the only CSF biomarkers “consistently associated” with pain in two and three studies involving respectively 67 patients for Flt-1 and 104 patients for CX3CL1 (both with a negative association). IL6, IL8, TGFbeta, VEGF and IP10 were “uncertainly not associated”, and others were “consistently not associated”.

Most biomarkers were related to inflammation and metabolism (Table S4). Of the six biomarkers “consistently associated” with pain, four were related to inflammation, i.e. IL17, VEGF, CX3CL1 and Flt-1. Moreover, biomarkers that were consistently associated with OA-related pain were also linked to inflammation (e.g. CRP, hsCRP and IL10). The other two biomarkers consistently associated with pain were C2C, a cartilage degradation biomarker and total cholesterol, a metabolic biomarker.

**Fig. 2:**
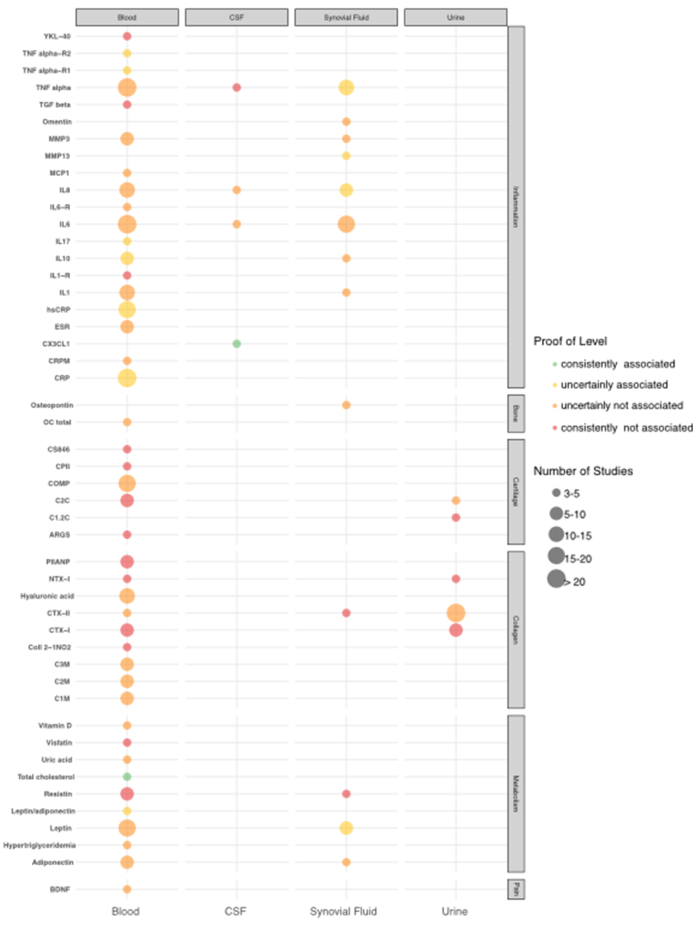
Dot plot map representing the association between the most studied circulating biomarkers and OA pain according to the categorization of biomarkers. Biomarkers associations are described in blood, SF, CSF and urine. Biomarkers consistently associated are represented in green, uncertainly associated in yellow, uncertainly not associated in orange and not associated in red. The size of each dot is proportional to the number of studies. **Note:** Only biomarkers evaluated in at least three studies were included in this figure. The classification as “consistently associated” reflects con**s**istent findings across studies, but not necessarily strong statistical associations, which remain weak in many cases. (For interpretation of the references to colour in this figure legend, the reader is referred to the Web version of this article.)

Two hundred and 33 fluid biomarkers were respectively assessed in only one and two studies. Fig. 2 shows the results for the 65 fluid biomarkers found in three or more studies. Of these, only two biomarkers, total cholesterol (in blood) and CX3CL1 (in CSF), met the criteria for a “consistent association” and were studied in at least three publications with consistent results. This highlights the limited number of biomarkers with sufficient data and consistent findings. Readers should bear in mind the low-to-moderate correlation coefficients shown in the meta-analyses (e.g. r ∼0.2–0.25 for CRP) when interpreting these associations, as these coefficients suggest weak statistical associations despite consistency.

Table S7 and Supplementary Figures present the overall correlation coefficients for the most widely studied biomarkers (also examined in at least three studies).

### 3.4 Relation between OA-related pain and other biomarkers

The tissue and genetic markers were analysed separately from the circulating biomarkers. Thirty-three studies assessed the relation between OA-related pain and biomarkers in non-fluid samples such as tissue samples, or genetic markers like SNPs.

In synovial tissue, 8 studies explored the relation of biomarkers with OA pain. The number of CD4^+^ T cells was “consistently associated” with VAS pain in two studies: de Jong and Klein-Wieringa [35,36]. The number of mast cells, Treg, or CD116 were “uncertainly associated” with VAS pain, each demonstrated in separate studies Farinelli, Nees and Pustjens [[37], [38], [39]]. GMCSF levels showed a negative correlation with VAS pain and were “consistently associated” (two studies: Pustjens and van Helvoort) [39,40]. Depending on the gender of the included patients, Shibata found different results between IL24 and OA-related pain (consistently associated and not associated, respectively, in women and men). Tsuchiya concluded that CD39^+^CD55^−^fibroblast-like cells were “consistently associated” with pain in 25 patients [41,42].

One study on the infrapatellar fat pad found that the levels of CD4^+^ cells, mast cells, CD8^+^ cells, and macrophages were “consistently not associated” with VAS pain (Klein-Wieringa) [36].

Similarly, an analysis of the acetabular labrum analysis by Sato et al., showed that VEGF and NGF were “consistently not associated” with VAS pain [43].

In the muscle tissue, levels of MCP-1 and atrogin-1 were found to be “uncertainly associated” with VAS pain (Levinger) [44].

Twenty-one studies assessed the genetic association of the SNPs with OA-related pain. For example, Abd Elazeem et al. reported a possible genetic association between GDF5 (+104T/C) single nuclear polymorphism and the severity (radiographic and pain) of Knee OA [45]. Schutte suggested the involvement of four genes (*EDNRA*, *COMT*, *BDRKB1*, and *IL1B*) in various pain components in 74 persons with knee OA [46].

### 3.5 Relationship between OA-related pain and biomarkers depending the type of pain measures

PRO measures were used in most pain assessments (97.0 ​%: 255/263 studies). In five studies, PPT was the only measure used to evaluate the association between OA pain and the fluid (*N* ​= ​3: Ahn, Bjurström and Puts) or the genetic biomarkers (*N* ​= ​2: Ho and Liu). The relationship between OA-related pain and 16 biomarkers was assessed using both PRO and PPT measures (Table S5). The only biomarker for which the results of the association with OA pain differed depending on whether the PPT or PRO was considered was Flt-1 in CSF, which was “consistently” associated with PPT and “uncertainly” associated with VAS. Table S5 shows the relationship between biomarkers and the different pain measures. No biomarker was associated with all the pain outcome measures. However, some differences can be seen. For example, vitamin D was consistently associated with the WOMAC index but not with the VAS scale.

## 4 Discussion

This study aimed to provide a comprehensive overview of the available evidence on OA-related pain biomarkers using a well-established methodology. We identified total blood cholesterol as the only blood biomarker with a consistent association with OA pain, as evidenced by multiple studies. CRP, VEGF and IL-17 were classified as “uncertainly associated”, and while their associations were observed in more than one study, the correlation strengths were weak (e.g., r ​∼ ​0.2) and the findings were not fully consistent. These findings highlight systemic low-grade inflammation as a key feature of OA and may explain the limited but observed effects of NSAIDs or intra-articular steroid injections in alleviating OA pain [[48], [49], [50], [51], [52]].

The consistent association between pain and total blood cholesterol, along with the uncertain associations with other metabolic or inflammatory markers, may reflect the influence of obesity-related processes in OA. The occurrence of metabolic syndrome in hand OA patients was associated with higher pain intensity [53]. However, in our study, the parameters of metabolic syndrome (triglycerides, HDL cholesterol and fasting blood glucose) were not associated with pain. The relationship between metabolic profile and OA pain may be more linked to inflammation than to metabolic syndrome. Obesity is characterised by a chronic low-grade inflammatory state [47] and many biomarkers categorised as either metabolic (e.g., cholesterol, insulin resistance) or inflammatory (e.g., CRP, leptin, adiponectin) may reflect overlapping pathophysiological pathways linked to adiposity [54,55]. Furthermore, there is a link between systemic inflammation and dyslipidaemia [56]. Therefore, the association between total cholesterol and OA pain may be more related to low-grade inflammation. However, it is difficult to draw strong conclusions due to the intricate relationship between all these parameters, and it is therefore plausible that some findings were partially or entirely confounded by obesity.

Blood TNF, IL6 or IL1 were found “uncertainly” not associated with OA pain, but blood IL17 was. These results illustrate the complexity of inflammatory network of cytokines: not all of them are associated at similar levels in OA-related pain. This complexity may explain why therapies that target these cytokines have achieved limited success in treating OA pain. Blocking TNF, IL1b, or IL6 did not provide efficacy over placebo in randomized controlled trial, although some improvement in structural and in systemic inflammation have been reported [[57], [58], [59]]. There does not seem to be a unique inflammatory process in OA but different types of inflammation at different tissue: systemic inflammation mediated by some cytokines, especially IL17, but also adipose inflammation [47]. Moreover, inconsistencies in the biological matrix (serum vs. plasma) used across studies may partly explain variability in biomarker levels and their associations with pain, underscoring the need for matrix-specific standardization in future research.

Our systematic review highlighted a consistent association between IL-17 levels in SF and OA-related pain, while its association in blood remains uncertain. IL17 is expressed at higher levels in inflamed OA synovium [60,61] and circulating IL17 levels play an important role in the pathogenesis and progression of inflammatory arthritis. Blocking IL17 with secukinumab or ixekizumab effective decreased systemic inflammation, synovitis, and the arthritis-related pain in spondyloarthropathies [[62], [63], [64]]. IL17 serum levels significantly correlates with cartilage defects, bone marrow lesions, and severity of OA in patients with knee osteoarthritis [65]. Thus, blocking IL17 in OA patients, such as in spondyloarthropathies, might decrease disease-associated inflammation and maybe also OA-related pain. However, until now no data about the efficacy of IL17 targeted therapies in OA or upcoming studies are available.

Increased VEGF levels are associated with OA progression. VEGF is involved in OA specific pathologies including cartilage degeneration, osteophyte formation, subchondral bone cysts and sclerosis, synovitis, and pain [66]. VEGF stimulates angiogenesis and vasodilation which has been attributed to OA progression. Furthermore, angiogenesis and inflammation are partly associated through VEGF, and VEGF has been associated with pain and sensitizes sensory neurons [67]. Inhibition of VEGF decreases OA progression and pain in animals models [68,69]. CSF Flt-1 consistently associated with pain in OA with a negative association. Flt-1 is a soluble splice variant of VEGFR-1 that acts as a VEGF decoy receptor. Flt-1 reduced OA and rheumatoid arthritis progression in animal models [70,71]. CX3CL1 (fractalkin) is a chemokine that is involved in T-cell recruitment in inflammation, e.g. to solid tumours [72]. CX3CL1 in CSF was consistently associated with lower knee pain intensity and milder symptoms [73]. These examples perfectly illustrate the need for identification of biomarkers associated with pain for identifying potential clinical value in treating pain.

Synovial NGF “uncertainly associated” with OA pain, potentially explain the observed pain improvement with NGF inhibitors (tanezumab) in OA [74,75]. Unfortunately, this treatment faced significant side effects that limited its application [76,77]. Similarly, blood CGRP was “uncertainly associated” with pain with a positive association. CGRP is a neuropeptide involved in neuronal pathways, crucial in nociception transmission and a potent vasodilator [78]. Its well-documented role in migraine pathophysiology where monoclonal anti-CGRP antibodies effectively prevent migraine [79] suggest a broader potential in treating in other peripheral pain types, including OA [80]. Notably, elevated serum CGRP levels have been documented in patient painful peripheral neuropathy [81]. Further exploration of the CGRP levels in OA patients could reveal its role in OA and inform trial assessing anti-CGRP treatment for OA pain.

C2C, a cartilage degradation biomarker was found “consistently associated” with OA pain with a positive association, when analysed in SF. However, C2C was not associated with pain in blood or urine, like the other cartilage degradation biomarkers (C1,2C; ADAMTS5, COMP), except for Fib 3-1 and 3-3 that were “uncertainly associated” in one study with 241 OA patients [82]. While bone and cartilage metabolism biomarkers generally lack strong associations with pain, several muscle metabolism indicators, including LDH and CPK, were “uncertainly associated” with pain. This highlights the importance of physical activity and sarcopenia prevention to decrease OA pain, as sarcopenia inversely associated with serum CPK levels based on a study with 1425 patients with knee or hip OA [83] and sarcopenia associated with higher prevalence of knee OA and pain group [84].

While certain synovial tissue or muscle tissue biomarkers were “consistently associated” with pain, we did not include them in the main results because they cannot be routinely and easily measured due to the invasive nature of the procedure to obtain the tissue (synovial biopsy or muscle biopsy). Although 12 studies have investigated genetic associations (e.g., SNPs) with OA-related pain, no consistent or replicated findings have emerged. These data have been included to reflect the broader biomarker landscape, but their role remains exploratory and no genetic variant can currently be considered a reliable OA-related pain biomarker. Future studies involving larger cohorts and functional validation are needed to determine their relevance. The association between genes and pain deserves to be further studied in OA patients to aid in developing targeted pain therapies, with recent studies linking genes like CHRFAM7A as novel genetic risk factor and therapeutic target for pain [85].

Our systematic review was not designed to assess the relation between the microbiome and OA-pain. Nevertheless, a few studies reported the potential role of the gut microbiome in OA-related pain [86], although it has been hypothesized that patients with OA-related pain exhibit an imbalance of the gut microbiota associated with pain intensity [87]. The abundance of *Streptococcus* sp. is associated with increased knee pain in the Rotterdam study, a large cohort of knee OA patients [88]. Given te relative unexplored role of microbiome, stool collection and microbiota analysis can also be part of multiomics analysis of OA pain.

Conclusions and treatments of chronic rheumatic diseases cannot be transposed in OA. It is therefore important to properly characterize pain mechanisms in OA patients by performing a multiomic analysis of biomarkers in blood, in SF, in synovial tissue, CSF and also in stool. A collaboration between European League Against Rheumatism and OMERACT working groups developed a consensual set of items for the analysis of synovial biopsies in clinical practice and translational research [89]. As our studies progress, a coordinated multiomic approach remains vital. To illustrate the idea of promoting and advocating a multiomic analysis to characterize OA pain, a cellular study was recently presented at the 2024 European League Against Rheumatism congress [90]. This study aimed to investigate whether and how Upadacitinib may affect pain-related and neuroinflammation-related molecules expression in monocyte-derived microglia, specifically regarding brain-derived neurotrophic factor.

Our study has some limitations. First, we would have liked to give a single result by doing a meta-analysis of the different included studies, which would have been more readable and more relevant for each biomarker. Our study’s design precluded a meta-analysis due to study heterogeneity, encompassing varied OA locations, pain assessments, and biomarker methodologies. Instead, we focused on a consistent classification system, categorizing biomarkers based on study count, sample size, and correlation strength. Despite these efforts, some categorizations remain open to interpretation, particularly with limited study numbers, as seen with IL17. Further studies will be welcome to increase the number of OA patients evaluated and the statistical power of the association. The method we chose to categorise each biomarker depended on our criteria, the number of studies and the sample size of patients within each study. The strength of the correlation is open to debate. Each biomarker classification was arbitrary, with some critical points, such as whether or not to consider adjustment. However, we attempted to mitigate this limitation by performing a classification of each biomarker discussed by all investigators and validated by consensus. It is plausible that some observed associations and findings are partially or entirely confounded by obesity or other parameters. Although a few of the included studies adjusted for BMI or other covariates such as age and physical activity, this was not uniformly reported, and our review was unable to account for these adjustments in the categorization of associations. This heterogeneity limits the strength of our conclusions. Future studies should systematically adjust for such confounders to better disentangle independent biomarker-pain relationships and identify specific mechanistic pathways in OA-related pain.

In conclusion, despite considerable effort has been devoted to identifying biological biomarkers of OA-related pain, none were found. The biomarkers of inflammation CRP and IL-17 levels showed the most promise, but further investigation is required. However, our study revealed that pain is often inadequately described in OA studies. The scientific community must conduct more detailed research on the relationship between different types of pain and biomarkers. The next steps in characterising OA-related pain and finding efficient treatments require OA biomarker studies to adopt a more standardised and objective approach to pain characterisation. This would involve multiomic assessment and further analysis of stool and synovial tissue specimens.

## Author contributions

All authors were involved in the drafting of this manuscript or critically revising it for important intellectual content, and all authors approved the final version to be submitted for publication.

**Study conception and design:** Sellam, Binvignat, Conaghan, Eijkelkamp, Henrotin, Kisand, Kosek, Kuhi, Mathieu, Mobasheri and Schaible.

**Acquisition of data:** Kuhi, Mathieu.

**Performing the figures:** Binvignat.

**Interpretation of the data, manuscript writing and revision:** Sellam, Binvignat, Conaghan, Eijkelkamp, Henrotin, Kisand, Kosek, Kuhi, Mathieu, Mobasheri and Schaible.

## Data availability statement

Data are available from the corresponding author upon reasonable request.

## Role of the funding source

NEURON-ERA-NET (Network of European Funding for Neuroscience Research) and Agence Nationale de la Recherche that fund the 2022 Going Inside Osteoarthritis-Related Pain network (for organization and logistical requirements for the network).

## Declaration of competing interest

LK, KK, HGS, MB, AB and NE reports no competing interest.

SM reports personal fees from BMS, Pfizer, AbbVie, Novartis, Roche, Chugai, Merck, Sharp, and Dohme, Tilman, but not related to the submitted work.

JS reports personal fees from MSD, Pfizer, Abbvie, Fresenius Kabi, BMS, Roche, Chugai, Sandoz, Lilly, Novartis, Galapagos, AstraZeneca, UCB, Grünenthal and Janssen and research grants from Pfizer, Schwa Medico, and BMS.

PC has done speakers bureaus or consultancies for AbbVie, AstraZeneca, Eli Lilly, Eupraxia, Galapagos, Genascence, GSK, Grunenthal, Janssen, Levicept, Medipost, Merck, Moebius, Novartis, Sandoz, Stryker, TrialSpark and UCB.

EK reports fees from Eli Lilly, Orion and UCB for lecturing or consulting.

YH reports fees from Artialis SA for lecturing or consulting.

## References

[1] Hunter D.J., March L., Chew M. (2020). Osteoarthritis in 2020 and beyond: a lancet commission. Lancet.

[2] Neogi T., Felson D., Niu J., Nevitt M., Lewis C., Aliabadi P. (2009). Association between radiographic features of knee osteoarthritis and pain: results from two cohort studies. BMJ.

[3] Kittelson A.J., Schmiege S.J., Maluf K., George S.Z., Stevens-Lapsley J.E. (2021). Determination of pain phenotypes in knee osteoarthritis using latent profile analysis. Pain Med..

[4] Zolio L., Lim K., McKenzie J., Yan M., Estee M., Hussain S. (2021). Systematic review and meta-analysis of the prevalence of neuropathic-like pain and/or pain sensitization in people with knee and hip osteoarthritis. Osteoarthr. Cartil..

[5] Raoof R., Willemen H., Eijkelkamp N. (2018). Divergent roles of immune cells and their mediators in pain. Rheumatology.

[6] Van Helvoort E., Welsing P., Jansen M., Gielis W., Loef M., Kloppenburg M. (2021). Neuropathic pain in the IMI-APPROACH knee osteoarthritis cohort: prevalence and phenotyping. RMD Open.

[7] Thakur M., Dickenson A., Baron R. (2014). Osteoarthritis pain: nociceptive or neuropathic?. Nat. Rev. Rheumatol..

[8] Dell’Isola A., Steultjens M. (2018). Classification of patients with knee osteoarthritis in clinical phenotypes : data from the osteoarthritis initiative. PLoS One.

[9] Deveza L., Nelson A., Loeser R. (2019). Phenotypes of osteoarthritis : current state and future implications. Clin. Exp. Rheumatol..

[10] Mobasheri A., Loeser R. (2024). Clinical phenotypes, molecular endotypes and theratypes in OA therapeutic development. Nat. Rev. Rheumatol..

[11] Meehan R., Regan E., Hoffman E., Wolf M., Gill M., Crooks J. (2021). Synovial fluid cytokines, chemokines, and MMP levels in osteoarthritis patients with knee pain display a profile siminal to many rheumatoid arthritis patients. J. Clin. Med..

[12] Mobasheri A., van Spil W., Budd E., Uzieliene I., Bernotiene E., Bay-Jensen A.C. (2019). Molecular taxonomy of osteoarthritis for patient stratification, disease management and drug development: biochemical markers associated with emerging clinical phenotypes and molecular endotypes. Curr. Opin. Rheumatol..

[13] Sarmanova A., Hall M., Fernandes G., Bhattacharya A., Valdes A., Walsh D. (2017). Associations between ultrasound-detected synovitis and knee pain: a population-based case-control study with both cross-sectional and follow-up data. Arthritis Res. Ther..

[14] Bacon K., LaValley P., Jafarzadeh S., Felson D. (2020). Does cartilage loss cause pain in osteoarthritis and if so, how much?. Ann. Rheum. Dis..

[15] Aso K., Shahtaheri S., Hill R., Wilson D., McWilliams D., Nwosu L. (2020). Contribution of nerves within osteochondral channels to osteoarthritis knee pain in humans and rats. Osteoarthr. Cartil..

[16] Obotiba A., Swain S., Kaur J., Yaseen K., Doherty M., Zhang W. (2021). Synovitis and bone marrow lesions associate with symptoms and radiographic progression in hand osteoarthritis: a systematic review and meta-analysis of observational studies. Osteoarthr. Cartil..

[17] Carlesso L., Segal N., Frey-Law L., Zhang Y., Na L., Nevitt M. (2019). Pain susceptibility phenotypes in those free of knee pain with or at risk of knee osteoarthritis: the multicenter osteoarthritis study. Arthritis Rheumatol..

[18] Mobasheri A., Thudium C., Bay-Jensen A.C., Maleitzke T., Geissler S., Duda G. (2023). Biomarkers for osteoarthritis: current status and future prospects. Best Pract. Res. Clin. Rheumatol..

[19] Hannani M., Thudium C., Karsdal M., Ladel C., Mobasheri A., Uebelhoer M. (2024). From biochemical markers to molecular endotypes of osteoarthritis: a review of validated biomarkers. Expert Rev. Mol. Diagn.

[20] Leung Y., Huebner J., Haaland B., Wong S., Kraus V. (2017). Synovial fluid pro-inflammatory profile differs according to the characteristics of knee pain. Osteoarthr. Cartil..

[21] Gao F., Hu Q., Chen W., Li J., Qi C., Yan Y. (2024). Brain regulates weight bearing bone through PGE2 skeletal interoception: implication of ankle osteoarthritis and pain. Bone Res..

[22] Li H., Li L., Min J., Yang H., Xu X., Yuan Y. (2012). Levels of metalloproteinase (MMP-3, MMP-9), NF-kappaB ligand (RANKL), and nitric oxide (NO) in peripheral blood of osteoarthritis (OA) patients. Clin. Lab..

[23] Collison J. (2019). Anti-NGF therapy improves osteoarthritis pain. Nat. Rev. Rheumatol..

[24] Kondo F., Takegami Y., Ishizuka S., Hasegawa Y., Imagama S. (2021). The association of the progression of knee osteoarthritis with high-sensitivity CRP in community-dwelling people-the Yakumo study. Clin. Rheumatol..

[25] Fernandes F., Pucinelli M., da Silva N., Feldman D. (2007). Serum cartilage oligomeric matrix protein (COMP) levels in knee osteoarthritis in a Brazilian population: clinical and radiological correlation. Scand. J. Rheumatol..

[26] Conaghan P., Cook A., Hamilton J., Tak P. (2019). Therapeutic options for targeting inflammatory osteoarthritic pain. Nat. Rev. Rheumatol..

[27] Ouzzani M., Hammady H., Fedorowicz Z., Elmagarmid A. (2016). Rayyan- a web and mobile app for systematic reviews. Syst. Rev..

[28] Stang A. (2010). Critical evaluation of the Newcastle-Ottawa scale for the assessment of the quality of nonrandomized studies in meta-analyses. Eur. J. Epidemiol..

[29] Bellamy N., Buchanan W., Goldsmith C., Campbell J., Stitt L. (1988). Validation study of WOMAC : a health status instrument for measuring clinical important patient relevant outcomes to antirheumatic drug therapy in patients with osteoarthritis of the hip or knee. J. Rheumatol..

[30] Roos E.M., Lohmander L.S. (2003). The knee injury and osteoarthritis outcome score (KOOS): from joint injury to osteoarthritis. Health Qual. Life Outcome.

[31] Nilsdotter A.K., Lohmander L.S., Klässbo M., Roos E.M. (2003). Hip disability and osteoarthritis outcome score (HOOS)–validity and responsiveness in total hip replacement. BMC Muscoskelet. Disord..

[32] Bellamy N., Campbell J., Haraoui B., Gerecz-Simon E., Buchbinder R., Hobby K. (2002). Clinimetric properties of the AUSCAN osteoarthritis hand index: an evaluation of reliability, validity and responsiveness. Osteoarthr. Cartil..

[33] Freynhagen R., Baron R., Gockel U., Tolle T. (2006). painDETECT : a new screening questionnaire to identify neuropathic components in patients with back pain. Curr. Med. Res. Opin..

[34] Park G., Kim C.W., Park S.B., Kim M.J., Jang S.H. (2011). Reliability and usefulness of the pressure pain threshold measurement in patients with myofascial pain. Ann. Rehabil. Med..

[35] de Jong A.J., de Lange-Brokaar B.J., Klein-Wieringa I., Heijink M., Hoekstra A., Everts B. (2016). Synovial CD4+ Tcells associate with pain in osteoarthritis: is there a role for fatty acids?. Osteoarthr. Cartil..

[36] Klein-Wieringa I.R., de Lange-Brokaar B., Yusuf E., Andersen S., Kwekkeboom J., Kroon H. (2016). Inflammatory cells in patients with endstage knee osteoarthritis: a comparison between the synovium and infrapatellar fat pad. J. Rheumatol..

[37] Farinelli L., Aquili A., Mattioli-Belmonte M., Manzotti S., D’Angelo F., Ciccullo C. (2022). Synovial mast cells from knee and hip osteoarthritis: histological study and clinical correlations. J. Exp. Orthop..

[38] Nees T., Rosshirt N., Zhang J.A., Platzer H., Sorbi R., Tripel E. (2020). T helper cell infiltration in osteoarthritis-related knee pain and disability. J. Clin. Med..

[39] Pustjens M., Mastbergen S., Lafeber F. (2014). Granulocyte-macrophage colony stimulating factor and its receptor CD116 expression in the synovium of osteoarthritis patients is negatively correlated with pain. Osteoarthr. Cartil..

[40] van Helvoort E., Eijkelkamp N., Labefer, Mastbergen S. (2020). Expression of granulocyte macrophage-colony stimulating factor and its receptor in the synovium of osteoarthritis patients is negatively correlated with pain. Rheumatology.

[41] Shibata N., Ohashi Y., Tsukada A., Iwase D., Aikawa J., Mukai M. (2024). IL24 expression in synovial myofibroblasts: implications for female osteoarthritis pain through propensity score matching analysis. Medicina.

[42] Tsuchiya M., Ohashi Y., Kodera Y., Satoh M., Matsui T., Fukushima K. (2023). CD39+CR55- Fb subset exhibits myofibroblast-like phenotype nad is associated with pain in osteoarthritis of the knee. Biomedicines.

[43] Sato Y., Tetsunaga T., Yamada K., Kawamura Y., Yoshida A., Ozaki T. (2023). Expression of acetabular labral vascular endothelial growth factor and nerve growth factor is directly associated with hip osteoarthritis pain: investigation by immunohistochemical staining. Int. J. Mol. Sci..

[44] Levinger P., Trenerry M., Levinger I., Feller J., Bartlett J., Bergman N. (2010). An increase in inflammatory markers in people with knee osteoarthritis is related to pain and altered walking pattern. Osteoarthr. Cartil..

[45] Abd Elazeem M., Abdelaleem E., Mohamed R. (2017). Genetic influence of growth and differentiation factor 5 gene polymorphism (+104T/C) on the development of knee osteoarthritis and its association with disease severity. Eur. J. Rheumatol..

[46] Schutte D., Mukhopadhyay N., Holwerda T., Sluka K., Rakel B., Govil M. (2020). Genetic predictors of knee pain in persons with mild to moderate osteoarthritis. Res. Gerontol. Nurs..

[47] Binvignat M., Sellam J., Berenbaum F., Felson T. (2024). The role of obesity and adipose tissue dysfunction in osteoarthritis pain. Nat. Rev. Rheumatol..

[48] Sanchez-Lopez E., Coras R., Torres A., Lane N., Guma M. (2022). Synovial inflammation in osteoarthritis progression. Nat. Rev. Rheumatol..

[49] Sellam J., Courties A., Eymard F., Ferrero S., Latourte A., Ornetti P. (2020). Recommendations of the French society of rheumatology on pharmacological treatment of knee osteoarthritis. Jt. Bone Spine.

[50] Gray B., Gibbs A., Bowden J., Eyles J., Grace S., Bennell K. (2024). Appraisal of quality and analysis of the similarities and differences between osteoarthritis clinical practice guideline recommendations: a systematic review. Osteoarthr. Cartil..

[51] Kolasinksi S., Neogi T., Hochberg M., Oatis C., Guyatt G., Block J. (2020). 2019 American college of rheumatology/arthritis foundation guideline for the management of osteoarthritis of the hand, hip, and knee. Arthritis Rheumatol..

[52] Bannuru R., Osani M., Vaysbrot E., Arden N., Bennell Bierma-Zeinstra S. (2019). KOARSI guidelines for the non-surgical management of knee, hip, and polyarticular osteoarthritis. Osteoarthr. Cartil..

[53] Charton A., Lacoste-Badie R., Tuffet S., Rousseau A., Maheu E., Fautrel B. (2025). Metabolic syndrome is associated with more pain in hand osteoarthritis : results from the DIGICOD cohort. Osteoarthr. Cartil. Open.

[54] Engin A. (2024). Reappraisal of adipose tissue inflammation in obesity. Adv. Exp. Med. Biol..

[55] Khanna D., Khanna S., Khanna P., Kahar P., Patel B.M. (2022). Obesity : a chronic low-grade inflammation and its markers. Cureus.

[56] Arida A., Protogerou A.D., Kitas G.D., Sfikakis P.P. (2018). Systemic inflammatory response and atherosclerosis : the paradigm of chronic inflammatory rheumatic diseases. Int. J. Mol. Sci..

[57] Mathieu S., Tournadre A., Soubrier M., Sellam J. (2022). Effect of disease-modifying anti-rheumatic drugs in osteoarthritis: a meta-analysis. Jt. Bone Spine.

[58] Estee M., Cicuttini F., Page M., Wluka A., Wang Y. (2023). Efficacy of tumor necrosis factor inhibitors in hand osteoarthritis: a systematic review and meta-analysis of randomized controlled trials. Osteoarthr. Cartil. Open.

[59] Richette P., Latourte A., Sellam J., Wendling D., Piperno M., Goupille P. (2021). Efficacy of tocilizumab in patients with hand osteoarthritis : double blind, randomised, placebo-controlled, multicentre trial. Ann. Rheum. Dis..

[60] Xiao J., Zhang P., Cai F.L., Luo G.C., Pu T., Pan X.L. (2023). IL-17 in osteoarthritis: a narrative review. Open Life Sci..

[61] Deligne C., Casulli S., Pigenet A., Bougault C., Campillo-Gimenez L., Nourissat G. (2015). Differential expression of interleukin-17 and interleukin-22 in inflamed and non-inflamed synovium from osteoarthritis patients. Osteoarthr. Cartil..

[62] Mease P.J., McInnes I., Kirkham B., Kavanaugh A., Rahman P., van der Heijde (2015). Secukinumab inhibition of interleukin-17A in patients with psoriatic arthritis. NEJM.

[63] Baeten D., Sieper J., Braun J., Baraliakos X., Dougados M., Emery P. (2015). Secukinumab inhibition of interleukin-17A in ankylosing spondylitis. NEJM.

[64] Nash P., Kirkham B., Okada M., Rahman P., Combe B., Burmester G.R. (2017). Ixekizumab for the treatment of patients with active psoriatic arthritis and an inadequate response to tumour necrosis factor inhibitors: results from the 24-week randomised, double-blind, placebo-controlled period of the SPIRIT-P2 phase 3 trial. Lancet.

[65] Wang K., Xu J., Cai J., Zheng S., Yang X., Ding C. (2017). Serum levels or resistin and interleukin-17 are associated with increased cartilage defects and bone marrow lesions in patients with knee osteoarthritis. Mod. Rheumatol..

[66] Hamilton J., Nagao M., Levine B., Chen D., Olsen B., Im H.J. (2016). Targeting VEGF and its receptor for the treatment of osteoarthritis and associated pain. J. Bone Miner. Res..

[67] Selvaraj D., Gangadharan V., Michalski C.W., Kurejova M., Stösser S., Srivastava K. (2015). A functional role for VEGFR1 expressed in peripheral sensory neurons in cancer pain. Cancer Cell.

[68] Nagai T., Sato M., Kobayashi M., Yokoyama M., Tani Y., Mochida J. (2014). Bevacizumab, an anti-vascular endothelial growth factor antibody, inhibits osteoarthritis. Arthritis Res. Ther..

[69] You S.A., Bae D.G., Ryoo J.W., Kim H.R., Park G.S., Cho C.S. (2005). Arginine-rich anti-vascular endothelial growth factor (anti-VEGF) hexapeptide inhibits collagen-induced arthritis and VEFG-stimulated productions of TNF-alpha and IL-6 by human monocytes. J. Immunol..

[70] Matsumoto T., Cooper G., Gharaibeh B., Meszaros L., Li G., Usas A. (2009). Cartilage repair in a rat model of osteoarthritis through intraarticular transplantation of muscle-derived stem cells expressing bone morphogenetic protein 4 and soluble Flt-1. Arthritis Rheum..

[71] Afuwape A., Feldmann M., Paleolog E. (2003). Adenoviral delivery of soluble VEGF receptor 1 (sFlt-1) abrogates disease activity in murine collagen-induced arthritis. Gene Ther..

[72] Do H.T.T., Lee C.H., Cho J. (2020). Chemokines and their receptors : multifaceted roles in cancer progression and potential value as cancer prognostic markers. Cancers.

[73] Palada V., Ahmed A.S., Freyhult E., Hugo A., Kultima K., Svensson C.I. (2020). Elevated inflammatory proteins in cerebrospinal fluid from patients with painful knee osteoarthritis are associated with reduced symptom severity. J. Neuroimmunol..

[74] Hochberg M., Carrino J., Schnitzer T., Guermazi A., Walsh D., White A. (2021). Long-term safety and efficacy of subcutaneous tanezumab versus nonsteroidal antiinflammatory drugs for hip and knee osteoarthritis. Arthritis Rheumatol..

[75] Berenbaum F., Blanco F., Guermazi A., Miki K., Yamabe T., Viktrup L. (2020). Subcutaneous tanezumab for osteoarthritis of the hip or knee : efficacy and safety results from a 24-week randomised phase III study within a 24-week follow-up period. Ann. Rheum. Dis..

[76] Carrino J., McAlindon T., Schnitzer T., Guermazi A., Hochberg M., Conaghan P. (2023). Characterization of adverse joint outcomes in patients with osteoarthritis treated with subcutaneous tanezumab. Osteoarthr. Cartil..

[77] Brown M., Cornblath D., Koltzenburg M., Gorson K., Hickman A., Pixton G. (2023). Peripheral nerve safety of nerve growth factor inhibition by tanezumab : pooled analyses of phase III clinical studies in over 5000 patients with osteoarthritis. Clin. Drug Invest..

[78] Brain S., Williams T., Tippins J., Morris H., MacIntyre I. (1985). Calcitonin gene-related peptide is a potent vasodilator. Nature.

[79] Ducros A., de Gaalon S., Roos A., Donnet A., Giraud P., Guegan-Massardier E. (2021). Revised guidelines of the French headache society for the diagnosis and management of migraine in adults. Part 2 : pharmacological treatment. Rev. Neurol..

[80] Moisset X. (2023). Are CGRP and PACAP involved in the pathophysiology of peripheral neuropathic pain. Rev. Neurol..

[81] Yevgi R., Laloglu E., Bilge N. (2023). High plasma calcitonin gene-related peptide and serum pituitary adenylate cyclase-activating polypeptide levels in patients with neuropathic pain. Rev. Neurol..

[82] Runhaar J., Sanchez C., Taralla S., Henrotin Y., Bierma-Zeinstra S. (2016). Fibulin-3 fragments are prognostic biomarkers of osteoarthritis incidence in overweight and obese women. Osteoarthr. Cartil..

[83] Kurita N., Kamitani T., Wada O., Shintani A., Mizuno K. (2021). Disentangling associations between serum muscle biomarkers and sarcopenia in the presence of pain and inflammation among patients with osteoarthritis : the SPSS-OK study. J. Clin. Rheumatol..

[84] Kim H.I., Ahn S.H., Kim Y., Lee J.E., Choi E., Seo S.K. (2022). Effects of sarcopenia and sarcopenic obesity on joint pain and degenerative osteoarthritis in postmenopausal women. Sci. Rep..

[85] Courties A., Olmer M., Myers K., Ordoukhanian P., Head S., Natarajan P. (2023). Human-specific duplicate CHRFAM7A gene is associated with more severe osteoarthritis and amplifies pain behaviours. Ann. Rheum. Dis..

[86] Sanchez Romero E., Melendez Oliva E., Alonso Perez J., Martin Perez S., Turroni S., Marchese L. (2021). Relationship between the gut microbiome and osteoarthritis pain : review of the literature. Nutrients.

[87] Turroni S., Pedersini P., Villafane J.H. (2021). The human gut microbiome and its relationship with osteoarthritis pain. Pain Med..

[88] Boer C., Radjabzadeh D., Medina-Gomez C., Garmaeva S., Schiphof D., Arp P. (2019). Intestinal microbiome composition and its relation to joint pain and inflammation. Nat. Commun..

[89] Najm A., Le Goff B., Orr C., Thurlings R., Canete J., Humby F. (2018). Standardisation of synovial biopsy analyses in rheumatic diseases : a consensus of the EULAR Synovitis and OMERACT Synovial tissue biopsy groups. Arthritis Res. Ther..

[90] Navarini L., Vomero M., Corberi E., Berardicurti O., Imperatori G., Currado D. (2024). Mechanisms of pain modulation by JAK inhibition: upadacitinib regulates pain-related pathways and BDNF expression in microglial cells. Ann. Rheum. Dis..

