# Supplementary material for: The relationship between circulating and tissue biomarkers and OA-related pain: A systematic literature review
Source: Osteoarthr Cartil Open. 2025 Sep 17;7(4):100684. doi: 10.1016/j.ocarto.2025.100684 (PMC12492238; doi:10.1016/j.ocarto.2025.100684)
Supplement: Multimedia component 1 [file mmc1.docx]

File S1. Search equations

Medline via PubMed: ("Osteoarthritis"[mesh] OR "Osteoarthritis"[tiab] OR "Osteo-arthritis"[tiab] OR osteoarthr*[tiab] OR osteo-arthr*[tiab] OR "osteoarthrosis"[tiab] OR "osteoarthroses"[tiab] OR "degenerative arthritis"[tiab] OR "arthrosis"[tiab] OR "arthroses"[tiab]) AND (“biomarkers”[MeSH] OR “biomarkers”[tiab] OR “soluble biomarkers”[tiab] OR CTX[tiab] OR COMP[tiab] OR “cartilage oligomeric matrix protein”[tiab] OR CRP[tiab] OR interleukin[tiab] OR alarmin[tiab] OR TNF[tiab] OR TGF[tiab] OR cytokine[tiab] OR chemokine[tiab] OR „growth factor“[tiab] OR “insulin resistance”[tiab] OR “glucose intolerance”[tiab] OR dyslipidemia[tiab] OR adiponectin[tiab] OR leptin[tiab] OR adipokine[tiab] OR C2C[tiab] OR CILP[tiab] OR insulin[tiab] OR NTX[tiab] OR BSAP[tiab] OR Coll2[tiab] OR C1M[tiab] OR C2M[tiab] OR C3M[tiab] OR genomics[tiab] OR proteomics[tiab] OR epigenomics[tiab] OR transcriptomics[tiab] OR lipidomics[tiab] OR metabolomics[tiab] OR immunomics[tiab] OR glycomics[tiab] OR genetic[tiab] OR “synovial membrane”[Mesh] OR synovial[tiab] OR synovium[tiab]) AND (pain[MeSH] OR pain[tiab] OR WOMAC[tiab] OR KOOS[tiab] OR „soreness“[tiab] OR „hyperalgesia“[tiab] OR ache[tiab] OR hurt[tiab]) NOT (animals[MeSH Terms] NOT humans[MeSH Terms])

Embase: ('osteoarthritis'/exp OR 'osteoarthritis':ti,ab,kw OR 'osteo-arthritis':ti,ab,kw OR osteoarthr*:ti,ab,kw OR 'osteo arthr*':ti,ab,kw OR 'osteoarthrosis':ti,ab,kw OR 'osteoarthroses':ti,ab,kw OR 'degenerative arthritis':ti,ab,kw OR 'arthrosis':ti,ab,kw OR 'arthroses':ti,ab,kw) AND ('biological marker'/exp OR 'biomarkers':ti,ab,kw OR 'soluble biomarkers':ti,ab,kw OR ctx:ti,ab,kw OR comp:ti,ab,kw OR 'cartilage oligometric matrix protein':ti,ab,kw OR crp:ti,ab,kw OR interleukin:ti,ab,kw OR alarmin:ti,ab,kw OR tnf:ti,ab,kw OR tgf:ti,ab,kw OR cytokine:ti,ab,kw OR chemokine:ti,ab,kw OR 'growth factor':ti,ab,kw OR 'insulin resistance':ti,ab,kw OR 'glucose intolerance':ti,ab,kw OR dyslipidemia:ti,ab,kw OR adiponectin:ti,ab,kw OR leptin:ti,ab,kw OR adipokine:ti,ab,kw OR c2c:ti,ab,kw OR cilp:ti,ab,kw OR insulin:ti,ab,kw OR ntx:ti,ab,kw OR bsap:ti,ab,kw OR coll2:ti,ab,kw OR c1m:ti,ab,kw OR c2m:ti,ab,kw OR c3m:ti,ab,kw OR genomics:ti,ab,kw OR proteomics:ti,ab,kw OR epigenomics:ti,ab,kw OR transcriptomics:ti,ab,kw OR lipidomics:ti,ab,kw OR metabolomics:ti,ab,kw OR immunomics:ti,ab,kw OR glycomics:ti,ab,kw OR genetic:ti,ab,kw OR 'synovium'/exp OR synovial:ti,ab,kw OR synovium:ti,ab,kw) AND ('pain'/exp OR pain:ti,ab,kw OR womac:ti,ab,kw OR koos:ti,ab,kw OR 'soreness':ti,ab,kw OR 'hyperalgesia':ti,ab,kw OR ache:ti,ab,kw OR hurt:ti,ab,kw) NOT ('animal'/exp NOT 'human'/exp)

Cochrane: ("Osteoarthritis":ti,ab,kw OR "Osteo-arthritis":ti,ab,kw OR osteoarthr*:ti,ab,kw OR osteo-arthr*:ti,ab,kw OR "osteoarthrosis":ti,ab,kw OR "osteoarthroses":ti,ab,kw OR "degenerative arthritis":ti,ab,kw OR "arthrosis":ti,ab,kw OR "arthroses":ti,ab,kw ) AND ( “biomarkers”:ti,ab,kw OR “soluble biomarkers”:ti,ab,kw OR CTX:ti,ab,kw OR COMP:ti,ab,kw OR “cartilage oligometric matrix protein”:ti,ab,kw OR CRP:ti,ab,kw OR interleukin:ti,ab,kw OR alarmin:ti,ab,kw OR TNF:ti,ab,kw OR TGF:ti,ab,kw OR cytokine:ti,ab,kw OR chemokine:ti,ab,kw OR “growth factor”:ti,ab,kw OR “insulin resistance”:ti,ab,kw OR “glucose intolerance”:ti,ab,kw OR dyslipidemia:ti,ab,kw OR adiponectin:ti,ab,kw OR leptin:ti,ab,kw OR adipokine:ti,ab,kw OR C2C:ti,ab,kw OR CILP:ti,ab,kw OR insulin:ti,ab,kw OR NTX:ti,ab,kw OR BSAP:ti,ab,kw OR Coll2:ti,ab,kw OR C1M:ti,ab,kw OR C2M:ti,ab,kw OR C3M:ti,ab,kw OR genomics:ti,ab,kw OR proteomics:ti,ab,kw OR epigenomics:ti,ab,kw OR transcriptomics:ti,ab,kw OR lipidomics:ti,ab,kw OR metabolomics:ti,ab,kw OR immunomics:ti,ab,kw OR glycomics:ti,ab,kw OR genetic:ti,ab,kw OR synovium:ti,ab,kw OR "synovial membrane":ti,ab,kw OR synovial:ti,ab,kw) AND (pain:ti,ab,kw OR WOMAC:ti,ab,kw OR KOOS:ti,ab,kw OR "soreness":ti,ab,kw OR "hyperalgesia":ti,ab,kw OR ache:ti,ab,kw OR hurt:ti,ab,kw )

Scopus: (INDEXTERMS(osteoarthr*) OR TITLE-ABS(osteoarthr*) OR TITLE-ABS(osteo-arthr*) OR TITLE-ABS(osteoarthros*) OR TITLE-ABS(“degenerative arthritis") OR TITLE-ABS(arthros*)) AND (TITLE-ABS(“biomarkers”) OR TITLE-ABS(“soluble biomarkers”) OR TITLE-ABS(CTX) OR TITLE-ABS(COMP) OR TITLE-ABS(“cartilage oligometric matrix protein”) OR TITLE-ABS(CRP) OR TITLE-ABS(interleukin) OR TITLE-ABS(alarmin) OR TITLE-ABS(TNF) OR TITLE-ABS(TGF) OR TITLE-ABS(cytokine) OR TITLE-ABS(chemokine) OR TITLE-ABS(”growth factor”) OR TITLE-ABS(“insulin resistance”) OR TITLE-ABS(“glucose intolerance”) OR TITLE-ABS(dyslipidemia) OR TITLE-ABS(adiponectin) OR TITLE-ABS(leptin) OR TITLE-ABS(adipokine) OR TITLE-ABS(C2C) OR TITLE-ABS(CILP) OR TITLE-ABS(insulin) OR TITLE-ABS(NTX) OR TITLE-ABS(BSAP) OR TITLE-ABS(Coll2) OR TITLE-ABS(C1M) OR TITLE-ABS(C2M) OR TITLE-ABS(C3M) OR TITLE-ABS(genomics) OR TITLE-ABS(proteomics) OR TITLE-ABS(epigenomics) OR TITLE-ABS(transcriptomics) OR TITLE-ABS(lipidomics) OR TITLE-ABS(metabolomics) OR TITLE-ABS(immunomics) OR TITLE-ABS(glycomics) OR TITLE-ABS(genetic) OR TITLE-ABS(“synovial membrane”) OR TITLE-ABS(synovial) OR TITLE-ABS(synovium)) AND (TITLE-ABS(pain) OR TITLE-ABS(WOMAC) OR TITLE-ABS(KOOS) OR TITLE-ABS(”soreness”) OR TITLE-ABS(”hyperalgesia”) OR TITLE-ABS(ache) OR TITLE-ABS(hurt))

Web of Science : (TS=osteoarthr* OR TS=osteo-arthr* OR TS=osteoarthros* OR TS=(“degenerative arthritis") OR TS=arthros*) AND (TS=“biomarkers” OR TS=(“soluble biomarkers”) OR TS=CTX OR TS=COMP OR TS=(“cartilage oligometric matrix protein”) OR TS=CRP OR TS=interleukin OR TS=alarmin OR TS=TNF OR TS=TGF OR TS=cytokine OR TS=chemokine OR TS=(”growth factor”) OR TS=(“insulin resistance”) OR TS=(“glucose intolerance”) OR TS=dyslipidemia OR TS=adiponectin OR TS=leptin OR TS=adipokine OR TS=C2C OR TS=CILP OR TS=insulin OR TS=NTX OR TS=BSAP OR TS=Coll2 OR TS=C1M OR TS=C2M OR TS=C3M OR TS=genomics OR TS=proteomics OR TS=epigenomics OR TS=transcriptomics OR TS=lipidomics OR TS=metabolomics OR TS=immunomics OR TS=glycomics OR TS=genetic OR TS= (“synovial membrane”) OR TS=synovial OR TS=synovium) AND (TS=pain OR TS=WOMAC OR TS=KOOS OR TS=”soreness” OR TS=”hyperalgesia” OR TS=ache OR TS=hurt) Not [abstracts and letters]

Table S1. Characteristics of the 263 included studies.

| Name first author | Reference journal | N patients | Pain scales | Biomarkers | | | | | Adjustments reported (e.g., BMI, age) |
| --- | --- | --- | --- | --- | --- | --- | --- | --- | --- |
|  |  |  |  | Blood  (S)=serum; (P)=plasma; (U)=unspecified | Urine | Synovial | CSF | Others |  |
| Abassifard | Iran J Allergy Asthma Immunol 2021 ;20 :114. | 23 | VAS | IL38 (S) |  |  |  |  | N |
| Abd Elazeem | Eur J Rheumatol 2017 ;4 :98 | 50 | WOMAC |  |  |  |  | Genes | Y |
| Abd Elghany | EJHM 2021 ;85 :4279 | 30 | VAS, WOMAC | Chemerin (S) |  |  |  |  | N |
| Ahn | Biol Res Nurs 2019 ;21 :400. | 40 | Other | b-endorphin (P) |  |  |  |  | Y |
| Alekseeva* | Aging Clin Exp Res 2023 ;35 :479 | 170 | VAS, WOMAC KOOS/HOOS | hsCRP, hTG (U) |  |  |  |  | N |
| Alexander | Arthritis Res Ther 2021 ;23 :226 | 25 | WOMAC | hsCRP, C1M, C3M, CRPM (S) |  |  |  |  | N |
| Altaie | Clin Exp Rheumatol 2024 ;42 :713 | 43 | VAS |  |  | bNGF, proNGF, NT-3, BDNF, LNGFR, TrkA |  |  | N |
| Arendt-Nielsen | Arthritis Rheum 2014 ;12 :3317 | 281 | VAS, Other | hsCRP, C1M, C2M, C3M, CRPM  (S) |  |  |  |  | N |
| Askari | PLoS One 2016 ;11 :e0164757 | 131 | WOMAC | IL17, IL21, IL23, vitamin D  (S) |  |  |  |  | N |
| Askari | Endocrine Regul 2020 ; 54 :6 | 150 | WOMAC | Leptin, resistin, adiponectin, visfatin,  (S) |  |  |  |  | N |
| Aslam | J Rheumatol. 2014 ;41 :938 | 638 | Other | COMP, C2C, CPII, HA,  (S) | CTXII, NTXI |  |  |  | Y |
| Attur | Arthritis Rheum 2011 ;63 :1908 | 96 | VAS, WOMAC |  |  |  |  | Genes | Y |
| Attur* | Arthritis Care Res. 2011 ;S61 | 194 | VAS, WOMAC |  |  |  |  | Genes | N |
| Awadallah | Journal American Science 2010 ;6 :1059 | 140 | VAS | COMP (S) |  |  |  |  | N |
| Azim | The Knee 2018 ;25 :25 | 98 | VAS, Other | IL6, leptin, TNFα,  (S) |  | IL6, leptin, TNFα | IL6, leptin, TNFα |  | N |
| Barker | Cytokine 2019 ;115 :45 | 29 | WOMAC | Cu/Zn SOD, GM-CSF, IL1, IL1-R, IL2, IL4, IL4-R, IL5, IL7, IL6, IL6-R, IL8, IL10, IL12, IL13, INFγ, MnSOD, TNFα, TNFα-R1, TNFα-R2  (S) |  |  |  |  | N |
| Barman* | Arch Phys Med Rehabil 2022 ;103 :81 | 96 | VAS | COMP, CRP, CTXII, ESR, HA, NTXI, PIIANP  (S) |  |  |  |  | N |
| Bas | Intern Orthop 2014 ;38 :2577 | 206 | VAS, WOMAC |  |  | Adiponectin, A/L, IL6, leptin, resistin, visfatin, |  |  | N |
| Bay-Jensen | Osteoarthr Cartil Open 2023 ;5 :100379 | 146 | WOMAC | C1M, C2M, C3M, C4M, PRO-C1, PRO-C2, PRO-C3, PRO-C4  (S) |  |  |  |  | Y |
| Bay-Jensen | Clinical Biochemistry 2018 ;58 :37 | 261 | VAS, WOMAC | C3A (S) |  |  |  |  | Y |
| Bay-Jensen* | Osteoarthr Cart 2016 ;24 :S87 | 216 | VAS, WOMAC | C3A (S) |  |  |  |  | Y |
| Bay-Jensen* | Osteoarthri Cart 2015 ;23 :A86 | 474 | WOMAC | COMP, CRPM, C3M  (S) | CTXII |  |  |  | Y |
| Bay-Jensen* | Osteoarthr Cart 2022 ;30 :S112 | 103 | WOMAC | CTXI, C1M  (S) | CTXI, CTXII, |  |  |  | Y |
| Bihlet | Arthritis Res Ther 2019 ;21 :23 | 1242 | WOMAC | OC total  (S) | CTXI, CTXII |  |  |  | Y |
| Bihlet* | Ann Rheum Dis 2015 ;74 :364 | 767 | WOMAC | CTXI  (S) | CTXII |  |  |  | Y |
| Binvignat | BioRxiv 2025 | 46 | WOMAC | IL22, IL2R, TNFR1, TNFR2  (S) |  |  |  |  | N |
| Binvignat | Osteoarthritis Cartilage Mars 2025 | 410 | Other | Calprotectin, CD14, FABP2, hsCRP, LBP, ZRP  (S) |  |  |  |  | Y |
| Bjurström | Pain Practice. 2022 ;22 :66 | 15 | Other | IL8, IP10  (P) |  |  | IP10, Flt-1 |  | N |
| Blichfeldt-Eckhardt | Bone Joint Res. 2024;13:741-74 | 50 | VAS | IL6R, LTα, TNFR1, TNFR2  (P) |  |  | IL1R, IL6, IL6R, TNFR1, TNFR2, |  | N |
| Cafferata | Oral Diseases. 2021 ;27 :970 | 5 | VAS |  |  | IL6, IL23, MMP1, MMP2, MMP8, MMP13, RANKL, RANKL/OPG, OPG, TNFα |  |  | N |
| Calvet | Osteoarthr Cart 2018 ; 26 :276 | 115 | WOMAC |  |  | Calprotectin, hsCRP |  |  | Y |
| Calvet* | Ann Rheum Dis 2018 ;77 :1605 | 108 | WOMAC |  |  | Adiponectin, chemerin, CRP, IL6, leptin, resistin, visfatin, osteopontin, omentin, TNFα |  |  | N |
| Calvet | Arthritis Res Ther 2024 ;26 :19 | 168 | KOOS | IL6  (P) |  |  | Omentin, osteopontin |  | Y |
| Chen | Clin Lab 2020 ;66 :1433 | 220 | WOMAC |  |  |  |  | Circ RNA | N |
| Cheng | Int J Rheum Dis 2020 ;23 :569 | 44 | KOOS/HOOS | C3-α, C3-β  (P) |  | C3-α, C3-β |  |  | N |
| Chiba | Arthritis Res Ther 2022 ;24 :269 | 297 | KOOS/HOOS | FBG  (S) |  |  |  |  | Y |
| Chong | Osteoarthr Cart Open 2023 ;5 :100405 | 137 | WOMAC | Adiponectin, hsCRP, leptin, resistin  (S) |  |  |  |  | N |
| Cioroianu | Rom J Morphol Embryol. 2024;65:217 | 46 | VAS | Total chol, ESR, hTG  (U) |  |  |  |  | N |
| Colombini | BMC Musculoskelet Dis 2023 ;24 :647 | 38 | KOOS/HOOS |  |  | C2C, CD11c/CD206, CPII, CTXII, HA, TNFα |  |  | N |
| Connelly* | Osteoarthr Cart 2014 ;22 :S66 | 54 | WOMAC | COMP, CRP, HA, MMP3, PIIANP  (S) |  |  |  |  | N |
| Costello* | Osteoarthr Cart 2022 ;30:S80 | 283 | Other |  |  |  |  | Genes | N |
| Dam | J Musculoskelet Pain 2011 ;19 :144 | 134 | VAS | CTXI  (S) | CTXII |  |  |  | Y |
| David Ho | Molecular Pain 2017 ;13 :1 | 161 | WOMAC Other |  |  |  |  | Genes | Y |
| de Jong* | Osteoarthr Cart 2016 ;24 :S321 | 40 | VAS |  |  |  |  | Synovial tissue | N |
| Dinç | Cartilage 2024 ;0(0) | 70 | VAS, WOMAC |  |  | IL1, IL6, TNFα |  |  | N |
| Ding* | Osteoarthr Cart 2011 ;19S1 :S40 | 149 | WOMAC | hsCRP, IL6, TNFα  (S) |  |  |  |  | N |
| Dong | Intern Orthop 2015 ;39 :1237 | 65 | WOMAC | CGRP  (S) |  | CGRP |  |  | N |
| Dong | Intern Orthop 2018 ;42 :1283 | 80 | VAS, WOMAC | Adiponectin, leptin, resistin  (P) |  | Adiponectin, leptin, resistin |  |  | N |
| Dorleijn | Osteoarthr Cart 2015 ;23 :57 | 222 | VAS, WOMAC |  | CTXII, CIIM |  |  |  | Y |
| Dündar | Int J Rheum Dis 2016 ;19 :287 | 40 | VAS, WOMAC | YKL-40  (S) |  |  |  |  | N |
| Eathakkattu* | Osteoarthr Cart 2021 ;29 :S93 | 156 | WOMAC | COMP, HA, MMP3  (S) |  |  |  |  | Y |
| Eitner | Pain 2017 ;158 : 1743 | 70 | KOOS/HOOS | CRP, hTG, HbA1C  (S) |  | IL6 |  |  | N |
| Ellabban | Life Sci J 2015 ;12 :38 | 36 | VAS | Leptin  (S) |  |  |  |  | N |
| Ellaithy | Egypt J Chem 2022 ; 6 :707 | 123 | WOMAC |  | CTXII |  |  |  | N |
| Elnemr | Hum Genomics. 2024;18:105 | 70 | VAS, WOMAC | Adiponectin  (S) |  |  |  |  | N |
| Erden | Turk J Phys Rehab 2022 ;33 :146 | 29 | VAS | IL1, IL6, TNFα  (S) |  |  |  |  | N |
| Esmayil | NUJHS 2014 ;4 :90 | 30 | VAS |  |  | SOD |  |  | N |
| Farinelli | J Exp Orthop 2022 ;9 :13 | 30 | VAS, KOOS/HOOS |  |  |  |  | Synovial tissue | N |
| Finckh* | Ann Rheum Dis 2013 ;71 :579 | 106 | VAS | Leptin  (S) |  |  |  |  | N |
| Fioravanti | Int J Biometeorol 2015 ;549 :1691 | 49 | VAS, WOMAC | Adiponectin, hsCRP, resistin, visfatin  (S) |  |  |  |  | N |
| Flores Bjurström | Pain 2020 ;161 :2142 | 52 | VAS, Other | IL8, IP-10  (S) |  |  | FlT-1, ICAM1, IL6, IL8, IL15, IP-10, MCP1, PIGF, VEGFA |  | Y |
| Gaballah | The Egyptian Rheumatol 2016 ;38 :29 | 20 | VAS |  |  |  | VEGF |  | N |
| Galvez | Int J Hypertherm 2018 ;35 :340 | 36 | VAS | IL8, TGFbeta  (S) |  |  |  |  | N |
| Gandhi | Clin Rheum 2010 ;29 :1223 | 60 | WOMAC Other |  |  | Adiponectin, A/L, leptin |  |  | N |
| García‑Manrique | Sci Rep 2021 ;11 :5258 | 115 | KOOS/HOOS | IL8  (P) |  | IL8 |  |  | Y |
| Garnero | Ann Rheum Dis 2001 ;60 :619 | 67 | WOMAC | COMP, CRP, CTXI, HA, OC total, PIIINP, YKL-40  (S) | CTXI, CTXII, Glc-Gal-PYD |  |  |  | N |
| Garnero | J Rheumatol 2005 ; 32 :697 | 376 | VAS | COMP, CRP, HA, MMP1, MMP3, PINP, YKL-40  (S) | CTXI, CTXII |  |  |  | Y |
| Georgiev | Rheumatology  2018 ;23 :4 | 132 | VAS | CRP  (U) |  |  |  |  | N |
| Giordano | Clin J Pain 2020 ;36 :229 | 127 | VAS | FGF-21  (S) |  |  |  |  | N |
| Gloersen* | Osteoarthr Cart 2021;29:S231 | 281 | VAS | hsCRP, leptin  (P) |  |  |  |  | N |
| Gómez-Aristizábal | Arthr Res Ther 2019 ;21 :26 | 83 | WOMAC KOOS/HOOS | CD14  (U) |  |  |  |  | Y |
| Guan | Clin Lab 2015 ;61 :991 | 144 | WOMAC | YKL-40  (S) |  | YKL-40 |  |  | Y |
| Guan | J Musculoskelet Neuronal Interact 2019 ;19 :326 | 99 | VAS |  |  | CCL20 |  |  | Y |
| Hafez* | QJM 2021 ;114 :i157 | 90 | VAS, WOMAC | Vitamin D  (U) |  |  |  |  | N |
| Haraden | Arthritis Res Ther 2019 ;21 :146 | 25 | Other |  |  | VCAM1, MMP3, ICAM1, MCP1, TIMP1, VEGF |  |  | N |
| Harsanyi* | Intern Med J 2012 | 27 | VAS | CRP  (U) |  |  |  |  | N |
| Hefferman | Knee. 2024;51:189 | 23 | KOOS, other | IL6  (S) |  |  |  |  | N |
| Herrero-Manley | Front Nutr 2023 ;10 :1126796 | 48 | VAS | CRP, hTG, uric acid, total chol, LDL chol, HDL chol  (S) |  |  |  |  | N |
| Hick | Cartilage 2021 ;13 :1637S | 121 | VAS, KOOS/HOOS | Coll2-1, Coll2-1NO2  (S) |  |  |  |  | N |
| Ho* | J Pain 2017 ;18 :S74 | 161 | Other |  |  |  |  | Genes | Y |
| Huang* | Osteoarthr Cart 2016 24 :1769 | 25 | WOMAC, Other | LPS, LBP  (S) |  | LPS, LBP |  |  | Y |
| Huebner | Osteoarthr Cart 2016 ;24 :1528 | 169 | WOMAC | IL6, leptin  (S) |  |  |  |  | N |
| Huebner* | Osteoarthr Cart 2018 ;28 :S42 | 25 | Other |  |  | ICAM1, MCP1,MMP3, TIMP1, VCAM1, VEGF |  |  | Y |
| Ilia | Medicina. 2024;60:571 | 24 | VAS, WOMAC |  |  | ACRP-30, IL6, IL10, TNFα |  |  | N |
| Imamura | Intern J Inflam 2015 :3269792 | 54 | VAS, WOMACOther | IL6, IL8, IL10, TNFα  (S) |  |  |  |  | N |
| Inoue | Osteoarthr Cart 2011 ;19 :51 | 616 | VAS, KOOS/HOOS | HA  (S) |  |  |  |  | N |
| Ishijima | Arthritis Res Ther 2011 ;13 :R22 | 46 | VAS | C2C, CPII, HA  (S) | CTXI, CTXII |  |  |  | N |
| Ismail S* | Osteoporos Int 2017;28: S497 | 72 | WOMAC | Angiopoietin 2  (U) |  |  |  |  | N |
| Jiang | Current Medical Science 2020 ; 40 :574 | 40 | VAS |  |  | DKK1 |  |  | N |
| Jurewicz | Genes 2022 ;13 :1775 | 207 | VAS, Other |  |  |  |  | Genes | N |
| Kalai* | Immuno-Anal Biol Spec 2012,27:6 | 125 | VAS | COMP  (S) |  |  |  |  | N |
| Kalogera | Int J Mol Sci 2023 ;24 :9463 | 31 | VAS, KOOS/HOOS, Other | ARGS, C1M, C2M, C3M, C10C  (S) |  | ARGS, C1M, C2M, C3M, C10C |  |  | Y |
| Kamel | Arch Rheumatol 2022 ;37 :187 | 70 | VAS, WOMAC, Other | IL17  (S) |  | IL17 |  |  | N |
| Kamiab | Adv Rheumatol. 2024;64:11 | 34 | VAS | IL17A, IL25  (S) |  |  |  |  | N |
| Kang | Int J Oral Maxillofac Surg 2007 ;36 :391 | 100 | VAS |  |  |  |  | Genes | N |
| Kapetanakis | Orthopedics 2010 ;33 :1938 | 63 | VAS | TGFbeta, TIMP1  (S) |  |  |  |  | N |
| Karimov* | Ann Rheum Dis 2023 ;82 :194 | 41 | VAS |  |  | IL4, IL6, IL10, TNFα |  |  | N |
| Kato | Acta Anaesthesiol Scand 2023 ;67 :1091 | 38 | Other |  |  |  | CSF-1, CX3CL1, IL6, IL8, TNFα |  | N |
| Keenan | Clin Exp Rheumatol 2008 ;26 :814 | 110 | VAS | CRP, ESR  (U) |  |  |  |  | N |
| Kim | J Korean Acad Rehab Med 2005 ;29 :87 | 30 | VAS | IGF-I  (S) |  | IGF-I |  |  | N |
| Kittelson* | J Pain 2014 ;15 :S257 | 21 | Other | CRP, IL6, IL8, TNFα  (U) |  |  |  |  | N |
| Klein-Wieringa | J Rheumatol 2016 ;43 :771 | 42 | VAS |  |  |  |  | Infrapatellar fat pad | Y |
| Klocke | Rheumatol Int 2018 ;38 :455 | 80 | WOMAC |  | CTXII |  |  |  | N |
| Kluzek* | Osteoarthr Cart 2014 ;22 :S74 | 724 | Other | COMP  (S) |  |  |  |  | Y |
| Kosek | J Neuroimmuno 2018 ;321 :48 | 40 | VAS, KOOS/HOOS, Other |  |  | IL6, IL8, MCP1 | IL6, IL8, MCP1 |  | N |
| Kraus | Ann Rheum Dis 2017 ;76 :186 | 194 | WOMAC | C1,2C, C2C, Coll2-1NO2, COMP, CPII, CS846, CTXI, HA, MMP3, NTX1, PIIANP  (S) | C1,2C, C2C, Coll2-1NO2, CTXI, CTXII, NTX1, |  |  |  | N |
| Kropáčková | BMC Musculoskelet Dis 2018 ;19 :264 | 135 | VAS, Other | Clusterin  (S) |  |  |  |  | N |
| Kumahashi | The Knee 2011 ;18 :160 | 28 | VAS |  |  | ATP |  |  | N |
| Lambova | Biomedicines 2021 ;9 :1019 | 73 | WOMAC | Leptin, resistin  (S) |  |  |  |  | N |
| Larsson | Osteoarthr Cart 2015 ;23 :1906 | 132 | KOOS/HOOS |  |  | IL6, IL8, TNFα |  |  | Y |
| Larsson | Osteoarthr Cart 2012 ;20 :388 | 141 | KOOS/HOOS |  |  | ARGS, MCP1 |  |  | Y |
| Larsson | Osteoarthr Cart 2024 ;32 :1463 | 233 | WOMAC | ARGS  (S) |  |  |  |  | N |
| Laskarin* | Ann Rheum Dis 2019 ;78 :1869 | 20 | VAS | B-GNLY  (U) |  |  |  |  | N |
| Lee | Arthritis Care Res 2011 ;63 :320 | 26 | Other | CRP, IL6, TNFα  (S) |  |  |  |  | N |
| Lee | Int J Vitam Nutr Res 2023 ;93 :410 | 121 | VAS |  |  |  |  | Genes | N |
| Lei | Benef Microbes 2017 ;8 :697 | 433 | VAS | hsCRP  (S) |  |  |  |  | N |
| Leung | Osteoarthr Cart 2017 ; 25 :1420 | 70 | VAS, WOMAC Other |  | CTXII | CTXI, CTXII, IL1, IL6, IL8, TNFα |  |  | Y |
| Levinger* | Osteoarthr Cart 2010;18:S31 | 19 | VAS |  |  |  |  | Muscle | N |
| Li | Scand J Clin Lab Invest 2012 ;72 :623 | 263 | WOMAC | Omentin  (S) |  | Omentin, IL1 |  |  | Y |
| Li | Ann Clin Biochemistry 2015 ;52 :276 | 161 | WOMAC | MCP1  (S) |  | MCP1 |  |  | Y |
| Li | Cartilage 2019 ;10 :408 | 143 | WOMAC |  |  | Eotaxin-I |  |  | Y |
| Li | BMC Musculoskelet Dis 2020 ;21:99 | 83 | VAS, Other |  |  | ADAMTS5, BK, CGRP, IL1, IL6, MCSF, MMP3, MMP13, NPY, SP, TNFα |  |  | N |
| Liem | Biomark Med 2022 ;16 :633 | 600 | WOMACKOOS/HOOS | COMP, Coll2-1, CPII, CS846, HA, PIIANP  (S) | CTXII, C1,2C |  |  |  | Y |
| Liem | Sci Rep 2020 ;10 :11328 | 580 | WOMAC,KOOS/HOOS | C1,2C, Coll2-1NO2, COMP, CPII, CS846, CTXI, HA, MMP3, NTXI, PIIANP  (S) | C1,2C, Coll2-1NO2, creatinin, CTXI, CTXII, NTXI |  |  |  | Y |
| Liem* | Arthritis Rheum 2022 ;74 (Suppl 9) | 415 | WOMAC | C3f  (S) |  |  |  |  | Y |
| Lindh | Scand J Rheum 1997;26:468 | 11 | VAS |  |  |  | SPLI |  | N |
| Liu | Plos One 2022 ;17 :e0262074 | 29 | VAS | BDNF, IL8, TNFα, TGFbeta  (P) |  |  | BDNF, IL8, TNFα, TGFbeta |  | N |
| Liu* | Osteoarthr Cart 2014 ;22 :S229 | 1199 | Other |  |  |  |  | Genes | N |
| Liu | Med Sci Monit 2015 ;21 :363 | 263 | WOMAC | BMP-2  (S) |  | IL17, BMP-2 |  |  | N |
| Liu | J App Biomed 2024 | 140 | VAS |  |  | CHOP, GRP78 |  |  | N |
| Loef | Osteoarthr Cart 2020 ;28 :223 | 906 | KOOS/HOOS, Other | Total FA, SFA, MUFA, omega3PUFA, omega6PUFA  (P) |  |  |  |  | N |
| Loukov | Osteoarthr Cart 2018 ;26 :255 | 22 | KOOS/HOOS | CRP, IL6, IL10, TNFα, int monocyte CCR2, classical monocyte CCR2, int monocyte HLADR, monocyte CD16  (S) |  |  |  |  | N |
| Lubbeke* | Arthritis Care Res 2011;63 | 250 | VAS, WOMAC |  |  | Leptin |  |  | N |
| Lubbeke | Int Orthop 2013 ;37 :2071 | 219 | VAS, WOMAC |  |  | Leptin |  |  | Y |
| Lundborg | J Neuroimmunol2010 ;220 :108 | 20 | VAS | GDNF, IL1, IL6, IL8, IL10, TNFα  (U) |  |  | GDNF, IL6, IL8, IL10, TNFα |  | N |
| Luo | Osteoarthr Cart Open 2020 ;2 :100082 | 163 | VAS | CTXII  (S) | CTXII |  |  |  | N |
| Marouf | Indian J. Pharmacol. 2021 ;53 :270 | 82 | VAS | IL1, IL6, TNFα  (S) |  |  |  |  | N |
| Martel-Pelletier | Osteoarthr Cart 2019 ;27 :1163 | 583 | WOMAC | Adiponectin, adipsin, chemerin, CRP, IL8, leptin, MCP1, visfatin  (S) |  |  |  |  | N |
| Massengale* | Plos One 2012;7:10 | 86 | VAS | Adiponectin, leptin, L/A, resistin  (S) |  |  |  |  | Y |
| Mehta | J Pain 2023 ;24 :1251 | 154 | VAS | Acylornithine, carnosine, cortisone, cortisol, cystine, DOPA, GLCAS, PEA, succinic acid  (S) |  |  |  |  | N |
| Messier | J Am Geriatr Soc 2000;48:1062 | 24 | Other | IL1  (S) |  |  |  |  | N |
| Miller* | Osteoarthr Cart 2022;30:S103 | 31 | KOOS/HOOS | ARGS, T2GM  IL1  (S) |  |  |  |  | N |
| Mishra | Am. J. Biochem. Biotechnol 2013 ;9 :1 | 300 | VAS, WOMAC |  |  |  |  | Genes | N |
| Mohammed | Indian J Public Health 2020 ;11 :1618 | 60 | VAS | Osteopontin  (P) |  | Osteopontin |  |  | N |
| Mohasseb | Mediterr J Rheumatol 2019 ;30 :114 | 47 | WOMAC |  |  |  |  | Genes | N |
| Mohasseb* | Osteoporos Int 2014;25:S162 | 25 | VAS | IL6  (U) |  |  |  |  | N |
| Moraes* | Osteoporos Int 2022;32 | 29 | VAS | CTXII  (S) |  |  |  |  | N |
| Mukundan* | Ann Rheum DIS 2022 ;81 :227 | 146 | WOMAC | C2M  (S) |  |  |  |  | N |
| Mundermann | F1000Res. 2024;12:299 | 24 | KOOS | C2C, MMP3  (S) |  |  |  |  | N |
| Muraki* | Osteoarthr Cart 2011 ;19 :1301 | 787 | Other | Vitamin D  (S) |  |  |  |  | Y |
| Najirman* | Int J Rheum Dis 2010;13:163 | 40 | VAS |  | CTXII |  |  |  | N |
| Nees | Biomedicines 2022 ;10 :2111 | 46 | VAS | Treg  (U) |  | Treg |  | Synovial tissue | N |
| Nees | J Clin Med 2020 ;9 :2423 | 47 | VAS |  |  |  |  | Synovial tissue | N |
| Nees | J Clin Med 2019 ;8 :1343 | 34 | VAS |  |  | CSF, IL6, IL7, IL8, IL10, IL12, IL13, IFNgamma, NGF, SGF, TNFα, VEGF |  |  | N |
| Neogi* | Arthritis Rheum 2009 | 1128 | Other |  |  |  |  | Genes | N |
| Nongmaithem | Indian J Rheumatol 2023 | 102 | VAS |  | CTXII |  |  |  | N |
| Nwosu | Osteoarthr Cart 2017;25:1428 | 129 | WOMAC | Cath-K  (S) |  |  |  |  | Y |
| Ogawa | Mod Rheumatol 2017;27:489 | 61 | Other |  |  | Lubricin |  |  | N |
| Ohashi | J Orthop Res 2022;40:1365 | 72 | VAS |  |  | CD163 |  |  | N |
| Oikonomidis | J Musculoskeletal Res 2017 ;20 :1750008 | 40 | VAS |  |  | TAC |  |  | N |
| Oliinyk* | Osteoarthr Cart 2020;28:S330 | 28 | WOMAC | hsCRP, leptin, resistin  (U) |  |  |  |  | N |
| Oliinyk* | Osteoarthr Cart 2016;24:S237 | 21 | WOMAC | IL1, CRP, TNFα  (U) |  |  |  |  | N |
| Orellana | Cartilage 2021;13:1675 | 115 | WOMAC | Adiponectin, leptin  (S) |  | Adiponectin, leptin, L/A |  |  | Y |
| Orita | BMC Musculoskeletal Dis 2011;12:144 | 47 | WOMAC | TNFα  (U) |  |  |  |  | N |
| Palada | J Neuroimmuno 2020;349 :577391 | 40 | VAS, KOOS/HOOS |  |  |  | CSF-1, CX3CL1, HGF, LIF-R, SCF, TWEAK, VEGFA |  | N |
| Pan | Pain Ther 2022;11:107 | 169 | WOMAC | IL6, CRP, TNFα  (S) |  |  |  |  | Y |
| Papaneophytou | BMC Musculoskeletal Dis 2022;23:195 | 174 | VAS | COMP, HA, PIICP  (S) |  |  |  |  | N |
| Peeler* | FASEB Journal 2019 ;33 :S1 | 26 | KOOS/HOOS | COMP  (U) |  |  |  |  | N |
| Pelletier* | Arthritis Rheum 2009;60:1944 | 155 | WOMAC | CRP  (S) |  |  |  |  | N |
| Penninx | J Rheumatol 2004 ;31 :2027 | 274 | WOMAC | CRP, IL6, IL6-R, IL1-R, TNFα, TNFα-R1, TNFα-R2  (S) |  |  |  |  | Y |
| Perruccio | Osteoarthr Cart Open 2019 ;1 :100004 | 196 | WOMAC | IL1, IL6, IL8, IL10, TNFα  (P) |  |  |  |  | Y |
| Pers | Theranostics 2018 ;8 :5519 | 18 | VAS, WOMAC | Int monocyte  (U) |  |  |  |  | N |
| Petersen | J Clin Pain 2016 ;32 :841 | 58 | VAS | C1M, C2M, C3M, hsCRP, CRPM  (S) |  |  |  |  | N |
| Philothra | Journal of Medicinal and Pharmaceutical Chemistry Research 2024 | 13 | Other |  | CTXII |  |  |  | N |
| Pustjens* | Osteoarthr Cart 2014 ;22 :S419 | 18 | VAS |  |  |  |  | Synovial tissue | N |
| Puts | J Clin Med. 2024 ;13:5212 | 17 | Other |  |  | CCL2, CXCL9, CXCL10, IL8 |  |  | N |
| Radojcic | Pain 2017 ;158 :1254 | 104 | WOMAC, Other | C1M (S),  IL6 (P) |  | IL6 |  |  | Y |
| Reijman | Arthritis Rheum 2004 ;50 :2471 | 460 | Other |  | CTXII |  |  |  | N |
| Ren | BMC Musculoskeletal Dis 2018 ;19 :39 | 50 | KOOS/HOOS | IL6, IL10  (S) |  |  |  |  | N |
| Richette | J Rheumatol 2008 ;35 :1650 | 42 | VAS |  |  | IL1, IL1-R |  |  | N |
| Riegger | J Clin Med 2020 ;9 :268 | 278 | WOMAC | COMP  (S) |  |  |  |  | N |
| Rotterud | BMC Musculoskeletal Dis 2014 ;15 :99 | 48 | KOOS/HOOS |  | CTXII |  |  |  | N |
| Ruan | Osteoarthr Cart 2019 ;27 :99 | 141 | WOMAC | S100A8/S100A9  (S) |  |  |  |  | Y |
| Ruan | Clin Rheum 2019 ;38 :3609 | 160 | WOMAC | IL8  (S) |  |  |  |  | Y |
| Runhaar | Osteoarthr Cart 2016 ;24 :672 | 241 | Other | Fib3-1, Fib3-2, Fib3-3  (S) |  |  |  |  | Y |
| Saengsiwaritt | Clin Transl Sci. 2023;16:2543 | 165 | VAS | LC3A  (S) |  |  |  |  | N |
| Saich* | Arthritis Rheum 2022 ;74 (Suppl9) | 23 | WOMAC, Other |  |  | NGF |  |  | N |
| Sandhu | Osteoarthr Cart 2024 ;32 :98 | 489 | WOMAC |  |  | C2C |  |  | N |
| Sato | Int J Mol Sci 2023 ;24 :2926 | 44 | VAS |  |  |  |  | Acetabular | N |
| Savitskaya* | Osteoarthr Cart 2011 ;19S1 :S8 | 75 | VAS | Dopamine-IgG  (S) |  |  |  |  | N |
| Schutte | Res Gerontol Nurs 2020 ;13 :191 | 75 | Other |  |  |  |  | Genes | N |
| Selistre | Braz J Phys Ther 2021 ;25 :62 | 25 | WOMAC |  | CTXII |  |  |  | N |
| Sellam | Semin Arthritis Rheum 2021 ;51 :129 | 863 | VAS, WOMAC | Adiponectin, ApoA1, ApoB, L/A, hsCRP, visfatin  (S) |  |  |  |  | Y |
| Sellam * | Ann Rheum Dis 2014;73 | 284 | WOMAC Other | L/A  (S) |  |  |  |  | N |
| Shao | BMC Musculoskeletal Dis ;24 :183 2023 | 30 | VAS |  |  | HMGB1 |  |  | N |
| Sharma | J Postgrade Med 2017 ;63 :151 | 499 | VAS |  |  |  |  | Genes | N |
| Shen | Front Med 2023 ;10 :1210170 | 12 | WOMAC | 13KODE, PGE2  (P) |  |  |  |  | N |
| Shibata | Medicina. 2024;60:741 | 68 | VAS |  |  |  |  | Synovial tissue | N |
| Shimura | Osteoarthr Cart 2013;21:1179 | 160 | VAS | IL6, hsCRP  (S) |  |  |  |  | Y |
| Shimura* | Osteoarthr Cart 2018 ;26 :S354 | 115 | VAS | IL6, HA  (S) |  |  |  |  | N |
| Si | BMC Musculoskeletal Dis 2017 :18 :265 | 96 | VAS | ASAT, CPK, CRP, ESR, IL6, IL8, LDH, myoglobin, creatinin, TNFα  (S) |  | IL6, IL8, PGE2, TNFα |  |  | N |
| Sibille | Clinl J Pain 2018 ;34 :182 | 167 | WOMAC | Omega6/omega3 PUFA  (P) |  |  |  |  | N |
| Siebuhr* | Arthritis Rheum 2013;65:S528 | 281 | Other | C1M, hsCRP  (S) |  |  |  |  | N |
| Simao | Rheumatol Int 2014 ;34 :1759 | 27 | WOMAC | BDNF, TNFα-R1, TNFα-R2  (P) |  |  |  |  | N |
| Singh* | Arthritis Rheum 2010;62:951 | 100 | Other | ICAM-1, TNFα  (S) |  |  |  |  | Y |
| Solignac* | Presse Med 2004 ;33 :1S13 | 507 | Other | CRP  (S) | CTXII |  |  |  | N |
| Song | J Clin Lab Anal 2016 ;30 :437 | 74 | WOMAC | Resistin  (S) |  | resistin |  |  | N |
| Sowers | Osteoarthritis Cartilage 2009 | 72 | Other | COMP  (S) | CTXII |  |  |  | N |
| Srivastava* | Osteoarthr Cart 2014 ;22 :S393 | 180 | VAS | Uric acid  (S) |  |  |  |  | N |
| Srivastava* | Osteoarthr Cart 2015 ;23 :A193 | 500 | WOMAC |  |  |  |  | Genes | N |
| Stabler* | Osteoarthr Cart 2010:18:S55 | 69 | Other |  |  | IL1, IL8, uric acid |  |  | N |
| Stannus* | Intern Med J 2011;41 :23 | 149 | WOMAC | hsCRP, IL6, TNFα  (S) |  |  |  |  | N |
| Strebkova* | Ann Rheum Dis 2017;76:968 | 25 | WOMAC | Leptin  (S) |  |  |  |  | N |
| Strebkova* | Osteoporos Int 2022;32: S373 | 50 | VAS, WOMAC | Leptin, mTOR  (S) |  |  |  |  | N |
| Sturmer | Ann Rheum Dis 2004 ;63 :200 | 770 | VAS, WOMAC | hsCRP  (S) |  |  |  |  | N |
| Sun | Disease Markers 2013 ;35 :203 | 226 | WOMAC | IL15  (S) |  |  |  |  | N |
| Sun | Innate Immun 2019 ;25 :255 | 101 | VAS |  |  | PACAP |  |  | N |
| Takahashi | Oral Surg Oral Med Oral Pathol Oral Radiol Endod 1999 ;88 :129 | 20 | Other |  |  | NO |  |  | N |
| Tamm* | Osteoarthr and Cart 2014 ;22 :S70 | 107 | KOOS/HOOS |  | C2C |  |  |  | N |
| Tarasovs | Int J Mol Sci. 2024;25:11918 | 50 | VAS |  | COMPTNF |  |  |  | N |
| Taskina* | Aging Clin. Exp. Res 2023 S244 | 116 | VAS, WOMAC | Leptin  (U) |  |  |  |  | N |
| Taskina* | Aging Clin. Exp. Res 2023 S484 | 183 | VAS | Total cholesterol  (U) |  |  |  |  | N |
| Tay* | New Zealand Med J 2023;136:106 | 35 | VAS | VEGF  (S) |  |  |  |  | N |
| Thudium* | Ann Rheum Dis 2017 ;76 :966 | 585 | Other | C3M  (S) |  |  |  |  | Y |
| Trifonova* | Ann Rheum Dis 2022 ;81 :897 | 137 | VAS | IL1, IL6, IL10, NO  (S) |  |  |  |  | N |
| Tsuchiya | Biomedicines. 2023;11:3047 | 25 | VAS |  |  |  |  | Synovial tissue | N |
| Turan | Clin Rheum 2007 ;26 :1293 | 58 | WOMAC | HA  (S) |  |  |  |  | N |
| Udomsinprasert | Int Immunopharmacol 2023 ;122 :110673 | 272 | VAS | Galectin3  (P) |  |  |  |  | N |
| Valdes | Arthritis Care Res 2011 ;63 :440 | 1854 | Other |  |  |  |  | Genes | N |
| Valdes | Ann Rheum Dis 2011 ;70 :1556 | 4368 | Other |  |  |  |  | Genes | Y |
| van Berkel | Osteoarthr Cart 2022 ;30 :1640 | 1002 | Other | hsCRP, PINP  (S) | CTXII |  |  |  | Y |
| van Helvoort | Rheumatology 2020 ;59 :3452 | 20 | VAS, WOMAC |  |  |  |  | Synovial tissue | N |
| van Meurs | Arthritis Rheum 2009 ;60 :628 | 288 | Other |  |  |  |  | Genes | N |
| Verma | J Orthop Res 2013 ;31 :999 | 100 | VAS | COMP  (S) |  |  |  |  | N |
| Villanova Lopez* | Euro. J. Hosp. Pharm. Sci. Pra. 2019;26:A199 | 38 | VAS | PDGF  (U) |  |  |  |  | N |
| Vincent | Open Orthop J 2013 ;7 :378 | 28 | VAS | IL1  (U) |  |  |  |  | N |
| Vincent* | Osteoarthr Cart 2018;26:S193 | 59 | WOMAC | CTXII, PIIANP  (S) |  |  |  |  | N |
| Waluyo | Bali Med J 2022;11:1151 | 26 | WOMAC |  | CTXII |  |  |  | N |
| Wang | Am J Transl Res 2022 ;14 :3915 | 104 | VAS | Cortisol, IL6, IL10  (S) |  |  |  |  | N |
| Wang | World J Clin Cases 2019 ;7 :2963 | 83 | VAS | IL6, IL10, TNFα  (S) |  |  |  |  | N |
| Wang | Journal of Jilin University Medicine Edition 2014;40:650 | 228 | VAS |  |  | MMP3, MMP9, MMP13, MMP14, uPA |  |  | N |
| Wang L* | Eur J Immunol 2019;49:922 | 23 | WOMAC | CRP, ESR  (S) |  |  |  |  | N |
| Wang L* | Neuropeptides 2016;55:6 | 100 | Other |  |  | Hexadecenoic acid |  |  | N |
| Wang Z | Arthritis Res Ther 2023;25:178 | 173 | WOMAC | Vitamin D  (S) |  |  |  |  | N |
| Warner | Front Immunol 2020 ;11 :1385 | 10 | Other | IL15  (S) |  |  |  |  | Y |
| Warner | Eur J Pain 2017 |  | Other |  |  |  |  | Genes | Y |
| Wen | Int J Mol Sci. 2024;25:12575 | 26 | VAS | BDNF, CX3CL1, IL1, IL6, MCP1, TGFβ, TNFα  (P) |  |  | Aβ40, BDNF, CX3CL1, IL1, IL6, MCP1, TGFβ, TNFα |  | N |
| Wislowska | Clin Rheum 2005;24:278 | 30 | WOMAC | COMP  (S) |  |  |  |  | N |
| Wolfe | J Rheumatol 1997;24:1486 | 572 | VAS | CRP, ESR  (U) |  |  |  |  | N |
| Wu | Osteoarthr Cart 2017;25:1428 | 146 | WOMAC | Ghrelin  (S) |  |  |  |  | N |
| Xie* | Osteoarthr Cart 2020;28:S330 | 6 | VAS |  |  | CD163, COMP, CTXII, GAG, IL1-R, IL6, IL8, MMP1, MMP3, TIMP1, TNFα, TSG-6 |  |  | N |
| Xin | Front Surg 2021;8:750047 | 37 | WOMAC | MMP13  (S) |  |  |  |  | N |
| Yan | Biomark Med 2022;16:731 | 1180 | VAS |  |  |  |  | Genes | N |
| Yang | Cells 2021;10:1826 | 447 | WOMAC | C3M, COMP, CRPM  (S) |  |  |  |  | Y |
| Yokohama* | Osteoarthr Cart 2023;31:S398 | 400 | VAS | Insulin resistance  (U) |  |  |  |  | N |
| Yu | Clin Chim Acta 2017;464:44 | 156 | WOMAC | CIR  (S) |  | CIR |  |  | N |
| Zhang | Med Sci Monit 2016;22:2182 | 226 | WOMAC | MIF  (S) |  | MIF |  |  | N |
| Zhang | BMC Musculoskeletal Dis 2018;19:22 | 2291 | Other | CRP  (S) |  |  |  |  | N |
| Zhou | Clin Lab 2018;64:577 | 74 | WOMAC | miR300  (S) |  |  |  |  | N |
| Zhu | Rheumatology 2022;61:1044 | 200 | WOMAC | Adiponectin, CRP, IL10, resistin  (S) |  |  |  |  | Y |
| Zietek | The knee 2016;23:1044 | 78 | VAS |  |  | TNFα |  |  | Y |
| Zou | Clin Chim Acta 2017;470:64 | 52 | VAS |  |  | Ghrelin |  |  | N |
| Zou | Biofactors 2019;45:463 | 97 | VAS |  |  | Ghrelin |  |  | Y |

* = abstracts. Signification of the biomarkers is in Table S2.

Note: For each included study, we indicated whether adjustments were reported for key confounding variables (specifically body mass index (BMI), age, sex, and other relevant covariates, when available) or not (Y= yes reported and N= not reported). This information is intended to help assess the potential influence of confounding factors on the observed associations between biomarkers and osteoarthritis (OA)-related pain.

Table S2. Circulating biomarkers analyzed. Signification and categorisation

| Biomarkers | Signification | Categorisation |
| --- | --- | --- |
| 13KODE | Ketodienoic compounds | Metabolism |
| PACAP | Pituitary adenylate cyclase-activating polypeptide | Inflammation |
| Aβ40 | Amyloid-β peptide 40 | Inflammation |
| ACRP-30 | Adiponectin (ACR-30) | Metabolism |
| Acyl ornithine |  | Metabolism |
| ADAMTS5 | A disintegrin and metalloproteinase with thrombospondin motifs 5 | Cartilage biomarker (degradation) |
| Adiponectin |  | Metabolism |
| Adiponectin/leptin= A/L |  | Metabolism |
| Adipsin |  | Metabolism |
| Angiopoietin-2 |  | Inflammation |
| ApoA1 | Apolipoprotein A1 | Metabolism |
| ApoB | Apolipoprotein B | Metabolism |
| ARGS | Autophagy-related genes | Collagen turnover (degradation) |
| ASAT | Aspartate aminotransferase | Muscle damage markers |
| ATP | Adenosine triphosphate | Metabolism |
| BDNF | Brain-derived neurotrophic | Pain sensitization |
| b-endorphin |  | Pain sensitization |
| B-GNLY | Granulysin | Inflammation |
| BMP2 | Bone morphogenic protein 2 | Bone degradation |
| b-NGF | b nerve growth factor | Pain sensitization |
| Bradykinin=BK |  | Inflammation |
| C1,2C | Col2-3/4 C-terminal cleavage product of types I and II collagen | Cartilage biomarker (degradation) |
| C1M |  | Type I Collagen turnover (degradation) |
| C2C | Col2-3/4 C-terminal cleavage product of human type II collagen | Cartilage biomarker (degradation) |
| C2M |  | Type II Collagen turnover (degradation) |
| C3A |  | Inflammation (Complement system activation) |
| C3-α |  | Inflammation (Complement system activation) |
| C3-β |  | Inflammation (Complement system activation) |
| C3f | C3 complement fragment | Collagen turnover (degradation) |
| C3M |  | Type III Collagen turnover (degradation) |
| C4M |  | Type IV Collagen turnover (degradation) |
| C10C | cathepsin-degraded type X collagen | Collagen turnover (degradation) |
| Calprotectin |  | Inflammation |
| Carnosine |  | Metabolism |
| Cath-K | Cathepsin K | Bone degradation |
| CCL2 | C-C motif ligand 2 | Inflammation |
| CCL20 | C-C motif ligand 20 | Inflammation |
| CD11c/CD206 |  | Inflammation |
| CD14 | Monocyte/macrophage chemokine | Inflammation |
| CGRP | calcitonin gene-related peptide | Pain sensitization |
| CD163 |  | Inflammation |
| CHOP | CCAAT/enhancer-binding protein homologous protein | Cartilage degradation |
| Chemerin |  | Metabolism |
| CIIM | collagen type II specific neoepitope | Type II collagen turnover (degradation) |
| CIR | Carbon isotope ratio | Metabolism |
| classical monocyte CCR2 |  | Inflammation |
| Clusterin | =Apolipoprotein J | Metabolism |
| Cortisone |  | Metabolism |
| Coll 2-1 | α-helical region of type II collagen | Type II collagen turnover (degradation) |
| Coll 2-1NO2 | nitrated epitope of the α-helical region of type II collagen | Type II collagen turnover (degradation) |
| COMP | Cartilage Oligometric matrix protein | Cartilage biomarker (degradation) |
| Cortisol |  | Metabolism |
| CPII | type II procollagen | Cartilage biomarker (degradation) |
| CPK | Creatin phosphokinase | Muscle damage markers |
| Creatinine |  | Muscle damage markers |
| CRP | C reactive protein | Inflammation |
| CRPM | Metabolite of CRP | Inflammation |
| CS846 | chondroitin sulfate 846 epitope | Cartilage biomarker (degradation) |
| CSF-1 | Colony-stimulating factor 1 | Inflammation |
| CTX-I | C-terminal telopeptides of type I collagen | Type I collagen turnover (degradation) |
| CTX-II | C-terminal telopeptides of type II collagen | Type II collagen turnover (degradation) |
| CU/Zn SOD | Copper/Zinc superoxide dismutase | Antioxidant |
| CX3CL1 | =Fractalkin | Inflammation |
| CXCL9 | C-X-C motif ligand 9 | Inflammation |
| CXCL10 | C-X-C motif ligand 10 | Inflammation |
| Cystine |  | Metabolism |
| DKK1 | Dickkopf-related protein 1 | Bone degradation |
| DOPA | Dihydroxyphenylalanine | Metabolism |
| Dopamine-IgG |  | Pain sensitization |
| Eotaxin-I |  | Inflammation (chemokine) |
| ESR | Erythrocyte sedimentation rate | Inflammation |
| FABP2 | Fatty acid binding protein 2 | Metabolism |
| Fib 3-1 | Fibulin 3-1 | Cartilage biomarker (degradation) |
| Fib 3-2 | Fibulin 3-2 | Cartilage biomarker (degradation) |
| Fib 3-3 | Fibulin 3-3 | Cartilage biomarker (degradation) |
| FGF-21 | Fibroblast growth factor 21 | Inflammation |
| Flt-1 | =VEGFR-1 Vascular endothelial growth factor receptor 1 | Inflammation (antiangiogenic factor) |
| GAG | Glycosaminoglycan | Cartilage degradation |
| Galectin3 |  | Inflammation |
| GDNF | glial cell line-derived neurotrophic factor | Pain sensitization |
| Ghrelin |  | Metabolism |
| GLCAS | Glycolithocholic acid sulphate | Metabolism |
| Glc-Gal-PYD | Galactopyranosyl-O-pyridinoline | Bone degradation |
| Glycemia =fasting blood glucose (FBG) |  | Metabolism |
| GM-CSF | Granulocyte-macrophage colony-stimulating factor | Inflammation |
| GRP78 | Glucose-regulated protein 78 | Cartilage degradation |
| Hexacetonid acid |  | Metabolism |
| HbA1C | Glycated hemoglobin | Metabolism |
| HDL cholesterol | High density lipoprotein cholesterol | Metabolism |
| HGF | Hepatocyte growth factor | Inflammation |
| HMGB1 | High-mobility group box-1 | Inflammation |
| hsCRP | High-sensitivity CRP | Inflammation |
| Hyaluronic acid=HA |  | collagen turnover (degradation) |
| Hypertriglyceridemia=hTG |  | Metabolism |
| ICAM-1 | intercellular adhesion molecule-1 | Inflammation |
| IGF-I | Insulin-like growth factor I | Metabolism |
| IL1 | Interleukin 1 | Inflammation |
| IL1-R | Interleukin 1 receptor | Inflammation |
| IL2 | Interleukin 2 | Inflammation |
| IL2-R | Interleukin 2 receptor | Inflammation |
| IL4 | Interleukin 4 | Inflammation |
| IL4-R | Interleukin 4 receptor | Inflammation |
| IL5 | Interleukin 5 | Inflammation |
| IL6 | Interleukin 6 | Inflammation |
| IL6-R | Interleukin 6 receptor | Inflammation |
| IL7 | Interleukin 7 | Inflammation |
| IL8 | Interleukin 8 | Inflammation |
| IL10 | Interleukin 10 | Inflammation |
| IL12 | Interleukin 12 | Inflammation |
| IL13 | Interleukin 13 | Inflammation |
| IL15 | Interleukin 15 | Inflammation |
| IL17 | Interleukin 17 | Inflammation |
| IL21 | Interleukin 21 | Inflammation |
| IL22 | Interleukin 22 | Inflammation |
| IL23 | Interleukin 23 | Inflammation |
| IL25 | Interleukin 25 | Inflammation |
| IL38 | Interleukin 38 | Inflammation |
| INFgamma | Interferon gamma | Inflammation |
| Insulin resistance |  | Metabolism |
| Int monocyte CCR2 |  | Inflammation |
| Int monocyte HLA-DR |  | Inflammation |
| IP-10 | Interferon gamma-induced  protein 10 | Inflammation |
| LBP | Lipopolysaccharide binding protein | Inflammation |
| LC3A | Microtubule-associated protein 1A/1B light chain 3A | Cartilage degradation |
| LDH | Lactate dehydrogenase | Muscle damage markers |
| LDL cholesterol | Low density lipoprotein cholesterol | Metabolism |
| Leptin |  | Metabolism |
| Leptin/adiponectin= L/A |  | Metabolism |
| LIF-R | leukemia inhibitory factor receptor | Inflammation |
| LNGFR | Low affinity nerve growth factor receptor | Pain sensitization |
| LPS | Lipopolysaccharide | Inflammation |
| Lubricin |  | Cartilage protection |
| LTα | Lymphotoxin alpha | Inflammation |
| MCP1 | Monocyte chemoattractant protein 1 | Inflammation |
| MCSF | Macrophage colony stimulating factor | Inflammation |
| MDA | Malonyldialdehyde | Inflammation |
| MIF | Macrophage migration in­hibitory factor | Inflammation |
| miR300 |  | Bone degradation |
| MMP1 | matrix metalloproteinase 1 | Inflammation |
| MMP2 | matrix metalloproteinase 2 | Inflammation |
| MMP3 | matrix metalloproteinase 3 | Inflammation |
| MMP8 | matrix metalloproteinase 8 | Inflammation |
| MMP9 | matrix metalloproteinase 9 | Inflammation |
| MMP13 | matrix metalloproteinase 13 | Inflammation |
| MMP14 | matrix metalloproteinase 14 | Inflammation |
| MnSOD | Manganese superoxide dismutase | Antioxidant |
| Monocyte CD16 |  | Inflammation |
| mTOR | Mechanistic targetof rapamycin | Inflammation |
| MUFA | monounsaturated fatty acids | Metabolism |
| Myoglobin |  | Muscle damage markers |
| Neuropeptide Y = NPY |  | Inflammation |
| NGF | nerve growth factor | Pain sensitization |
| NO | Nitric oxide | Inflammation |
| NTXI | N-telopeptide of type I collagen | Type I collagen turnover (degradation) |
| Number Treg= Treg |  | Inflammation |
| NT-3 | Neurotrophin 3 | Pain sensitization |
| OC total | Osteocalcin total | Bone Anabolism |
| Omega-3 PUFA | Omega-3 polyunsaturated fatty acids | Metabolism |
| Omega-6 PUFA | Omega-6 polyunsaturated fatty acids | Metabolism |
| Omega-6/omega-3 PUFA | Omega-6/omega-3 polyunsaturated fatty acids | Metabolism |
| Omentin |  | Anti-inflammatory |
| Osteopontin |  | Bone resorption |
| Osteoprotegerin=OPG |  | Bone turnover |
| PDGF | Platelet derived growth factor | Inflammation |
| PEA | Phenylethylamine | Metabolism |
| PGE2 | Prostagladin E2 | Inflammation |
| PIIANP | N-terminal propeptide of collagen IIA | Collagen Anabolism |
| PIICP | C-terminal propeptide | Collagen Anabolism |
| PIIINP | N-terminal propeptide of type III procollagen | Bone Anabolism |
| PIGF | Placenta growth factor | Inflammation (Pro-angiogenic factor) |
| PINP | N-terminal propeptide of type I procollagen | Bone Anabolism |
| PRO-C1 |  | Collagen turnover (Anabolism) |
| PRO-C2 |  | Collagen turnover (Anabolism) |
| PRO-C3 |  | Collagen turnover (Anabolism) |
| PRO-C4 |  | Collagen turnover (Anabolism) |
| ProNGF | Pro Nerve growth factor | Pain sensitization |
| RANKL | Receptor activator of nuclear factor-kappaB ligand | Bone degradation |
| RANKL/OPG | Receptor activator of nuclear factor-kappaB ligand/osteoprotegerin | Bone degradation |
| Resistin |  | Metabolism |
| S100A8/S100A9 | Alarmins S100A8 and S100 A9 | Inflammation |
| SCF | stem cell factor | Inflammation |
| SFA | saturated fatty acids | Metabolism |
| SCGF-beta | stem cell growth factor | Inflammation |
| SPLI | Secretory leukocyte protease inhibitor | Inflammation |
| Substance P = SP |  | Pain sensitization |
| Succinic acid |  | Metabolism |
| SOD | Superoxide dismutase | Antioxidant |
| T2GM | Type 2 gene mean | Cartilage degradation |
| TAC | Total antioxidant capacity | Antioxidant |
| TGF beta | Transforming growth factor beta | Inflammation |
| TIMP | Tissue inhibitor of metalloproteinases | Anti-inflammatory |
| TIMP-1 | Tissue inhibitor of metalloproteinases-1 | Anti-inflammatory |
| TNF alpha | Tumor necrosis factor alpha | Inflammation |
| TNF alpha-R1 | Tumor necrosis factor alpha receptor 1 | Inflammation |
| TNF alpha-R2 | Tumor necrosis factor alpha receptor 2 | Inflammation |
| Total cholesterol |  | Metabolism |
| Total Fatty Acid= Total FA |  | Metabolism |
| Total PUFA | Total polyunsaturated fatty acids | Metabolism |
| TrkA | Tropomyosin receptor kinase A | Pain sensitization |
| TSG-6 | Tumor necrosis factor- stimulated gene-6 | Inflammation |
| uPA | urokinase-type plasminogen activator | Inflammation |
| Uric acid |  | Metabolism |
| VCAM-1 | Vascular cell adhesion molecule 1 | Inflammation |
| VEGF | Vascular endothelial growth factor | Inflammation |
| VEGFA | Vascular endothelial growth factor A | Inflammation |
| Visfatin |  | Metabolism |
| Vitamin D |  | Metabolism |
| TWEAK | Fibroblast growth factor 21 | Inflammation |
| YKL-40 | =Chitinase-3-like protein 1 (CHI3L1) | Inflammation |
| ZRP | Zonulin-related proteins | Inflammation |

Table S3. Association between circulating biomarkers and OA pain. Number of included studies (N) and OA patients (n).

| Biomarkers | Blood  (S)= serum  (P)= plasma  (U)= unspecified | Urine | Synovial fluid | CS fluid |
| --- | --- | --- | --- | --- |
| 13KODE | N=1 ; n=12 (P) |  |  |  |
| Aβ40 |  |  |  | N=1; n=26 |
| ACRP-30 |  |  | N=1; n=24 |  |
| Acyl ornithine | N=1 ; n=154 (S) |  |  |  |
| ADAMTS5 |  |  | N=1 ; n=83 |  |
| Adiponectin | N=10; n=2,198 (S) |  | N=5; n=576 |  |
| Adiponectin/leptin |  |  | N=2; n=266 |  |
| Adipsin | N=1; n=583 (S) |  |  |  |
| Angiopoietin-2 | N=1 ; n=2 (U) |  |  |  |
| ApoA1 | N=1, n=863 (S) |  |  |  |
| ApoB | N=1 ; n=863 (S) |  |  |  |
| ARGS | N=3; n=295 (S) |  | N=2; n=172 |  |
| ASAT | N=1 ; n=96 (S) |  |  |  |
| ATP |  |  | N=1 ; n=28 |  |
| BDNF | N=3; n=82 (P) |  | N=1; n=43 | N=2; n=55 |
| BDNF/LNGFR |  |  | N=1; n=43 |  |
| b-endorphin | N=1 ; n=40 (P) |  |  |  |
| B-GNLY | N=1 ; n=20 (U) |  |  |  |
| BMP2 | N=1 ; n=37 (S) |  | N=1 ; n=37 |  |
| b-NGF |  |  | N=1 ; n=42 |  |
| Bradykinin |  |  | N=1 ; n=83 |  |
| C1,2C | N=2; n=774 (S) | N=3; n=1,374 |  |  |
| C1M | N=8; n=1,029 (S) |  | N=1; n=31 |  |
| C2C | N=6; n=2,082 (S) | N=4; n=905 | N=2; n=527 |  |
| C2M | N=6; n=884 (S) |  | N=1; n=31 |  |
| C3A | N=2; n=477 (S) |  |  |  |
| C3-α | N=1 ; n=44 (P) |  | N=1 ; n=44 |  |
| C3-β | N=1 ; n=44 (P) |  | N=1 ; n=44 |  |
| C3F | N=1 ; n=415 (S) |  |  |  |
| C3M | N=8; n=2,047 (S) |  | N=1; n=31 |  |
| C4M | N=1 ; n=146 (S) |  |  |  |
| C10C | N=1; n=31 (S) |  | N=1 ; n=31 |  |
| Calprotectin | N=1; n=410 (S) |  | N=1; n=108 |  |
| Carnosine | N=1 ; n=154 (S) |  |  |  |
| Cath-K | N=1 ; n=129 (S) |  |  |  |
| CCL2 | N=1 ; n=17 (S) |  |  |  |
| CCL20 |  |  | N=1 ; n=99 |  |
| CD11c/CD206 |  |  | N=1; n=38 |  |
| CD14 | N=2 ; n=493 (S) |  |  |  |
| CD163 |  |  | N=1; n=73 |  |
| CGRP | N=1; n=65 (S) |  | N=2; n=148 |  |
| CHOP |  |  | N=1; n=140 |  |
| Chemerin | N=2; n=613 (S) |  | N=1; n=108 |  |
| CIIM |  | N=1 ; n=222 |  |  |
| CIR | N=1 ; n=156 (S) |  | N=1; n=156 |  |
| classical monocyte CCR2 | N=1 ; n=22 (S) |  |  |  |
| Clusterin | N=1 ; n=135 (S) |  |  |  |
| Cortisone | N=1 ; n=154 (S) |  |  |  |
| Coll 2-1 | N=1 ; n=121 (S) |  |  |  |
| Coll 2-1NO2 | N=4; n=1,495 (S) | N=2; n=774 |  |  |
| COMP | N=20; n=5,715 (S) | N=1; n=50 | N=1; n=6 |  |
| Cortisol | N=2; n=258 (S) |  |  |  |
| CPII | N=5; n=2,058 (S) |  | N=1; n=38 |  |
| CPK | N=1 ; n=96 (S) |  |  |  |
| Creatinine | N=1 ; n=96 (S) | N=1 ; n=580 |  |  |
| CRP | N=23; n=6,036  (S=18; U=5) |  |  |  |
| CRPM | N=5; n=1,285 (S) |  |  |  |
| CS846 | N=3; 1,374 (S) |  |  |  |
| CSF-1 |  |  |  | N=2; n=78 |
| CTX-I | N=6; n=1,731 (S) | N=6; 2,562 | N=1; n=70 |  |
| CTX-II | N=5; n=1443 (S) | N=28; n=7,916 | N=3; n=114 |  |
| CU/Zn SOD | N=1 ; n=29 (S) |  |  |  |
| CX3CL1 | N=1 ; n=26 (P) |  |  | N=3; n=104 |
| CXCL9 | N=1 ; n=17 (S) |  |  |  |
| CXCL10 | N=1 ; n=17 (S) |  |  |  |
| Cystine | N=1 ; n=154 (S) |  |  |  |
| DKK1 |  |  | N=1 ; n=40 |  |
| DOPA | N=1 ; n=154 (S) |  |  |  |
| Dopamine-IgG | N=1 ; n=75 (S) |  |  |  |
| Eotaxin-I |  |  | N=1; n=143 |  |
| ESR | N=6; n=943  (S=3; U=3) |  |  |  |
| FABP2 | N=1 ; n=410 (S) |  |  |  |
| Fib 3-1 | N=1 ; n=241 (S) |  |  |  |
| Fib 3-2 | N=1 ; n=241 (S) |  |  |  |
| Fib 3-3 | N=1 ; n=241 (S) |  |  |  |
| FGF-21 | N=1 ; n=127 (S) |  |  |  |
| Flt-1 |  |  |  | N=2; n=67 |
| GAG |  |  | N=1 ; n=6 |  |
| Galectin3 | N=1 ; n=272 (P) |  |  |  |
| GDNF | N=1 ; n=20 (U) |  |  | N=1 ; n=20 |
| Ghrelin | N=1 ; n=146 (S) |  | N=2; n=149 |  |
| GLCAS | N=1 ; n=154 (S) |  |  |  |
| Glc-Gal-PYD |  | N=1 ; n=67 |  |  |
| Glycemia (FBG) | N=1; n=297 (S) |  |  |  |
| GM-CSF | N=1 ; n=29 (S) |  |  |  |
| GRP78 |  |  | N=1; n=140 |  |
| Hexacetonid acid |  |  | N=1; n=100 |  |
| HbA1C | N=1 ; n=70 (S) |  |  |  |
| HDL cholesterol | N=1; n=48 (S) |  |  |  |
| HGF |  |  |  | N=1 ; n=40 |
| HMGB1 |  |  | N=1 ; n=30 |  |
| hsCRP | N=19; n=5,372  (S=18; U=1) |  | N=1; n=115 |  |
| Hyaluronic acid | N=14; n=3,770 (S) |  | N=1; n=38 |  |
| Hypertriglyceridemia | N=4; n=334  (S=2; U=2) |  |  |  |
| ICAM-1 | N=1 ; n=100 (S) |  | N=1 ; n=25 | N=1 ; n=52 |
| IGF-I | N=1 ; n=30 (S) |  | N=1 ; n=30 |  |
| IL1 | N=12; n=704  (S=7; P=2; U=3) |  | N=4; n=485 | N=1; n=26 |
| IL1-R | N=3; n=345 (S) |  | N=1; n=6 | N=1; n=50 |
| IL2 | N=1 ; n=29 (S) |  |  |  |
| IL2-R | N=1 ; n=46 (S) |  |  |  |
| IL4 | N=1 ; n=29 (S) |  | N=1 ; n=41 |  |
| IL4-R | N=1 ; n=29 (S) |  |  |  |
| IL5 | N=1 ; n=29 (S) |  |  |  |
| IL6 | N=28; n=2,445  (S=21; P=4; U=3) |  | N=16; n=1,293 | N=5; n=234 |
| IL6-R | N=3; n=353  (S=2 or P=1) |  |  | N=1 ; n=50 |
| IL7 | N=1 ; n=29 (S) |  | N=1 ; n=34 |  |
| IL8 | N=12; n=1,356  (S=6; P=4; U=2) |  | N=8; n=562 | N=4; n=127 |
| IL10 | N=10; n=895  (S=7; P=2; U=1) |  | N=3; n=99 | N=1; n=20 |
| IL12 | N=1 ; n=29 (S) |  | N=1 ; n=34 |  |
| IL13 | N=1 ; n=29 (S) |  | N=1 ; n=34 |  |
| IL15 | N=1 ; n=226 (S) |  |  | N=1 ; n=52 |
| IL17 | N=3; n=235 (S) |  | N=2; n=296 |  |
| IL21 | N=1 ; n=131 (S) |  |  |  |
| IL22 | N=1 ; n=46 (S) |  |  |  |
| IL23 | N=1 ; n=131 (S) |  | N=1 ; n=5 |  |
| IL25 | N=1 ; n=34 (S) |  |  |  |
| IL38 | N=1 ; n=23 (S) |  |  |  |
| INFgamma | N=1 ; n=29 (S) |  | N=1 ; n=34 |  |
| Insulin resistance | N=1; n=400 (U) |  |  |  |
| Int monocyte CCR2 | N=1 ; n=22 (U) |  |  |  |
| Int monocyte HLA-DR | N=1 ; n=22 (U) |  |  |  |
| IP-10 | N=1 ; n=15 (S) |  |  | N=1 ; n=52 |
| LBP | N=2 ; n=435 (S) |  | N=1 ; n=25 |  |
| LC3A | N=1 ; n=165 (S) |  |  |  |
| LDH | N=1 ; n=96 (S) |  |  |  |
| LDL cholesterol | N=1; n=48 (S) |  |  |  |
| Leptin | N=16; n=2,098  (S=13; P=1; U=2) |  | N=8; n=1,143 | N=1; n=98 |
| Leptin/adiponectin | N=3; n=1,230 (S) |  | N=1; n=115 |  |
| LIF-R |  |  |  | N=1 ; n=40 |
| LNGFR |  |  | N=1 ; n=43 |  |
| LPS | N=1 ; n=25 (S) |  | N=1 ; n=25 |  |
| LTα | N=1; n=50 (P) |  |  |  |
| Lubricin |  |  | N=1 ; n=61 |  |
| MCP1 | N=3; n=770  (S=2; P=1) |  | N=2; n=201 | N=2; n=66 |
| M-CSF |  |  | N=2; n=117 |  |
| MDA |  |  | N=1 ; n=40 |  |
| MIF | N=1 ; n=226 (S) |  | N=1; n=226 |  |
| miR300 | N=1 ; n=74 (S) |  |  |  |
| MMP1 | N=1; n=376 (S) |  | N=2; n=11 |  |
| MMP2 |  |  | N=1 ; n=5 |  |
| MMP3 | N=6; n=1,384 (S) |  | N=4; n=342 |  |
| MMP8 |  |  | N=1 ; n=5 |  |
| MMP9 |  |  | N=1; n=228 |  |
| MMP13 | N=1; n=37 (S) |  | N=3; n=316 |  |
| MMP14 |  |  | N=1; n=228 |  |
| Mn SOD | N=1 ; n=29 (S) |  |  |  |
| Monocyte CD16 | N=1 ; n=22 (S) |  |  |  |
| mTOR | N=1 ; n=50 (S) |  |  |  |
| MUFA | N=1 ; n=533 (P) |  |  |  |
| Myoglobin | N=1 ; n=96 (S) |  |  |  |
| Neuropeptide Y |  |  | N=2; n=183 |  |
| NGF |  |  | N=2; n=57 |  |
| NGF/LNGFR |  |  | N=1; n=43 |  |
| NGF/TrKA |  |  | N=1; n=43 |  |
| NO | N=1; n=137 (S) |  | N=1; n=10 |  |
| NTX-I | N=3; n=870 (S) | N=4; n=1,458 |  |  |
| Number Treg | N=2; n=64  (S=1; U=1) |  | N=1; n=46 |  |
| NT-3 |  |  | N=1 ; n=43 |  |
| NT-3/LNGFR |  |  | N=1 ; n=43 |  |
| OC total | N=3; n=2,311 S) |  |  |  |
| Omega-3 PUFA | N=1 ; n=533 (P) |  |  |  |
| Omega-6 PUFA | N=1 ; n=533 (P) |  |  |  |
| Omega-6/omega-3 PUFA | N=1 ; n=167 (P) |  |  |  |
| Omentin | N=1 ; n=263 (S) |  | N=3; n=546 |  |
| Osteopontin | N=1; n=60 (P) |  | N=3; n=343 |  |
| Osteoprotegerin |  |  | N=1 ; n=5 |  |
| PACAP |  |  | N=1; n=101 |  |
| PDGF | N=1 ; n=38 (S) |  |  |  |
| PEA | N=1 ; n=154 (S) |  |  |  |
| PGE2 | N=2; n=108  (S=1; P=1) |  | N=2; n=166 |  |
| PIIANP | N=6; n=1,589 (S) |  |  |  |
| PIICP | N=1 ; n=174 (S) |  |  |  |
| PIIINP | N=2; n=443 (S) |  |  |  |
| PIGF |  |  |  | N=1 ; n=52 |
| PINP | N=2; n=1,378 (S) |  |  |  |
| PRO-C1 | N=1 ; n=146 (S) |  |  |  |
| PRO-C2 | N=1 ; n=146 (S) |  |  |  |
| PRO-C3 | N=1 ; n=146 (S) |  |  |  |
| PRO-C4 | N=1 ; n=146 (S) |  |  |  |
| ProNGF |  |  | N=1 ; n=43 |  |
| ProNGF/LNGFR |  |  | N=1 ; n=43 |  |
| RANKL |  |  | N=1 ; n=5 |  |
| RANKL/OPG |  |  | N=1 ; n=5 |  |
| Resistin | N=10; n=1,148  (S=8; P=1; U=1) |  | N=4; n=475 |  |
| S100A8/S100A9 | N=1; n=141 (S) |  |  |  |
| SCF |  |  |  | N=1 ; n=40 |
| SFA | N=1 ; n=533 (P) |  |  |  |
| SCGF-beta |  |  | N=1 ; n=34 |  |
| SPLI |  |  |  | N=1 ; n=11 |
| Substance P |  |  | N=1 ; n=83 |  |
| Succinic acid | N=1 ; n=154 (S) |  |  |  |
| SOD |  |  | N=1 ; n=30 |  |
| T2GM | N=1 ; n=31 (S) |  |  |  |
| TAC |  |  | N=2; n=70 |  |
| TGF beta | N=4; n=154  (S=2; P=2) |  |  | N=2; n=55 |
| TIMP | N=1 ; n=63 (S) |  | N=1 ; n=6 |  |
| TIMP-1 |  |  | N=1 ; n=25 |  |
| TNF alpha | N=22; n=1,699  (S=15; P=3; U=4) | N=1; n=50 | N=13; n=820 | N=4; n=173 |
| TNF alpha-R1 | N=5; n=426  (S=3; P=2) |  |  | N=1; n=50 |
| TNF alpha-R2 | N=5; n=426  (S=3; P=2) |  |  | N=1; n=50 |
| Total cholesterol | N=3; n=277  (S=1; U=2) |  |  |  |
| Total Fatty Acid | N=1 ; n=533 (P) |  |  |  |
| Total PUFA | N=1 ; n=533 (P) |  |  |  |
| TrkA |  |  | N=1 ; n=43 |  |
| TSG-6 |  |  | N=1 ; n=6 |  |
| uPA |  |  | N=1; n=228 |  |
| Uric acid | N=3; n=298 (S) |  | N=1; n=69 |  |
| VCAM-1 |  |  | N=1; n=25 | N=1 ; n=52 |
| VEGF | N=2; n=55 (S) |  | N=2; n=59 | N=2; n=92 |
| VEGFA |  |  |  | N=1 ; n=40 |
| Visfatin | N=4; n=1,645 (S) |  | N=2; n=321 |  |
| Vitamin D | N=3; n=1,050  (S=2; U=1) |  |  |  |
| TWEAK |  |  |  | N=1 ; n=40 |
| YKL-40 | N=4; n=627 (S) |  | N=1; n=144 |  |
| ZRP | N=1; n=410 (S) |  |  |  |

N= number of included studies; n=number of OA patients; CS=cerebrospinal

| Colour significance | Consistently not associated | Uncertainly not associated | Uncertainly associated | Consistently  associated |
| --- | --- | --- | --- | --- |

Table S4. Association between circulating biomarkers and OA pain depending the categorization of biomarkers

| Biomarkers | Association with OA pain depending on the fluid | | | |
| --- | --- | --- | --- | --- |
|  | Blood | Urine | Synovial | CSF |
| Anti-inflammatory/antioxidant | | | | |
| CU/Zn SOD |  |  |  |  |
| Mn SOD |  |  |  |  |
| Omentin |  |  |  |  |
| SOD |  |  |  |  |
| TAC |  |  |  |  |
| TIMP |  |  |  |  |
| TIMP-1 |  |  |  |  |
| Bone degradation | | | | |
| BMP2 |  |  |  |  |
| Cath-K |  |  |  |  |
| DKK1 |  |  |  |  |
| Glc-Gal-PYD |  |  |  |  |
| miR300 |  |  |  |  |
| Osteopontin |  |  |  |  |
| Osteoprotegerin |  |  |  |  |
| RANKL |  |  |  |  |
| RANKL/OPG |  |  |  |  |
| Bone formation | | | | |
| OC total |  |  |  |  |
| PIIINP |  |  |  |  |
| PINP |  |  |  |  |
| Cartilage degradation | | | | |
| ADAMTS5 |  |  |  |  |
| ARGS |  |  |  |  |
| C1,2C |  |  |  |  |
| C2C |  |  |  |  |
| CHOP |  |  |  |  |
| COMP |  |  |  |  |
| CPII |  |  |  |  |
| CS846 |  |  |  |  |
| Fib 3-1 |  |  |  |  |
| Fib 3-2 |  |  |  |  |
| Fib 3-3 |  |  |  |  |
| GAG |  |  |  |  |
| GRP78 |  |  |  |  |
| LC3A |  |  |  |  |
| T2GM |  |  |  |  |
| Cartilage anabolism/protection | | | | |
| Lubricin |  |  |  |  |
| PRO-C1 |  |  |  |  |
| PRO-C2 |  |  |  |  |
| PRO-C3 |  |  |  |  |
| PRO-C4 |  |  |  |  |
| Collagen degradation | | | | |
| C1M |  |  |  |  |
| C2M |  |  |  |  |
| C3F |  |  |  |  |
| C3M |  |  |  |  |
| C4M |  |  |  |  |
| C10C |  |  |  |  |
| CIIM |  |  |  |  |
| Coll 2-1 |  |  |  |  |
| Coll 2-1NO2 |  |  |  |  |
| CTX-I |  |  |  |  |
| CTX-II |  |  |  |  |
| Hyaluronic acid |  |  |  |  |
| NTX-I |  |  |  |  |
| Collagen formation | | | | |
| PIIANP |  |  |  |  |
| PIICP |  |  |  |  |
| Inflammation |  |  |  |  |
| Aβ40 |  |  |  |  |
| Angiopoietin-2 |  |  |  |  |
| B-GNLY |  |  |  |  |
| Bradykinin |  |  |  |  |
| C3A |  |  |  |  |
| C3-α |  |  |  |  |
| C3-β |  |  |  |  |
| Calprotectin |  |  |  |  |
| CCL2 |  |  |  |  |
| CCL20 |  |  |  |  |
| CD11c/CD206 |  |  |  |  |
| CD14 |  |  |  |  |
| CD163 |  |  |  |  |
| Classical monocyte CCR2 |  |  |  |  |
| CRP |  |  |  |  |
| CRPM |  |  |  |  |
| CSF-1 |  |  |  |  |
| CX3CL1 |  |  |  |  |
| CXCL9 |  |  |  |  |
| CXCL10 |  |  |  |  |
| Eotaxin-I |  |  |  |  |
| ESR |  |  |  |  |
| Flt-1 |  |  |  |  |
| FGF-21 |  |  |  |  |
| Galectin3 |  |  |  |  |
| GM-CSF |  |  |  |  |
| HGF |  |  |  |  |
| HMGB1 |  |  |  |  |
| hsCRP |  |  |  |  |
| ICAM-1 |  |  |  |  |
| IL1 |  |  |  |  |
| IL1-R |  |  |  |  |
| IL2 |  |  |  |  |
| IL2-R |  |  |  |  |
| IL4 |  |  |  |  |
| IL4-R |  |  |  |  |
| IL5 |  |  |  |  |
| IL6 |  |  |  |  |
| IL6-R |  |  |  |  |
| IL7 |  |  |  |  |
| IL8 |  |  |  |  |
| IL10 |  |  |  |  |
| IL12 |  |  |  |  |
| IL13 |  |  |  |  |
| IL15 |  |  |  |  |
| IL17 |  |  |  |  |
| IL21 |  |  |  |  |
| IL22 |  |  |  |  |
| IL23 |  |  |  |  |
| IL25 |  |  |  |  |
| IL38 |  |  |  |  |
| INFgamma |  |  |  |  |
| Int monocyte CCR2 |  |  |  |  |
| Int monocyte HLA-DR |  |  |  |  |
| IP-10 |  |  |  |  |
| LBP |  |  |  |  |
| LIF-R |  |  |  |  |
| LPS |  |  |  |  |
| LTα |  |  |  |  |
| MCP1 |  |  |  |  |
| M-CSF |  |  |  |  |
| MDA |  |  |  |  |
| MIF |  |  |  |  |
| MMP1 |  |  |  |  |
| MMP2 |  |  |  |  |
| MMP3 |  |  |  |  |
| MMP8 |  |  |  |  |
| MMP9 |  |  |  |  |
| MMP13 |  |  |  |  |
| MMP14 |  |  |  |  |
| Monocyte CD16 |  |  |  |  |
| mTOR |  |  |  |  |
| Neuropeptide Y |  |  |  |  |
| NO |  |  |  |  |
| Number Treg |  |  |  |  |
| PACAP |  |  |  |  |
| PDGF |  |  |  |  |
| PGE2 |  |  |  |  |
| PIGF |  |  |  |  |
| S100A8/S100A9 |  |  |  |  |
| SCF |  |  |  |  |
| SCGF-beta |  |  |  |  |
| SPLI |  |  |  |  |
| TGF beta |  |  |  |  |
| TNF alpha |  |  |  |  |
| TNF alpha-R1 |  |  |  |  |
| TNF alpha-R2 |  |  |  |  |
| TSG-6 |  |  |  |  |
| uPA |  |  |  |  |
| VCAM-1 |  |  |  |  |
| VEGF |  |  |  |  |
| VEGFA |  |  |  |  |
| TWEAK |  |  |  |  |
| YKL-40 |  |  |  |  |
| ZRP |  |  |  |  |
| Metabolism | | | | |
| 13KODE |  |  |  |  |
| ACRP-30 |  |  |  |  |
| Acyl ornithine |  |  |  |  |
| Adiponectin |  |  |  |  |
| Adiponectin/leptin |  |  |  |  |
| Adipsin |  |  |  |  |
| ApoA1 |  |  |  |  |
| ApoB |  |  |  |  |
| ATP |  |  |  |  |
| Carnosine |  |  |  |  |
| Chemerin |  |  |  |  |
| CIR |  |  |  |  |
| Clusterin |  |  |  |  |
| Cortisone |  |  |  |  |
| Cortisol |  |  |  |  |
| Cystine |  |  |  |  |
| DOPA |  |  |  |  |
| FABP2 |  |  |  |  |
| Ghrelin |  |  |  |  |
| GLCAS |  |  |  |  |
| Glycemia (FBG) |  |  |  |  |
| Hexacetonid acid |  |  |  |  |
| HbA1C |  |  |  |  |
| HDL cholesterol |  |  |  |  |
| Hypertriglyceridemia |  |  |  |  |
| IGF-I |  |  |  |  |
| Insulin resistance |  |  |  |  |
| LDL cholesterol |  |  |  |  |
| Leptin |  |  |  |  |
| Leptin/adiponectin |  |  |  |  |
| MUFA |  |  |  |  |
| Omega-3 PUFA |  |  |  |  |
| Omega-6 PUFA |  |  |  |  |
| Omega-6/omega-3 PUFA |  |  |  |  |
| PEA |  |  |  |  |
| Resistin |  |  |  |  |
| SFA |  |  |  |  |
| Succinic acid |  |  |  |  |
| Total cholesterol |  |  |  |  |
| Total Fatty Acid |  |  |  |  |
| Total PUFA |  |  |  |  |
| Uric acid |  |  |  |  |
| Visfatin |  |  |  |  |
| Vitamin D |  |  |  |  |
| Muscle damage markers | | | | |
| ASAT |  |  |  |  |
| CPK |  |  |  |  |
| Creatinine |  |  |  |  |
| LDH |  |  |  |  |
| Myoglobin |  |  |  |  |
| Pain sensitization | | | | |
| BDNF |  |  |  |  |
| BDNF/LNGFR |  |  |  |  |
| b-endorphin |  |  |  |  |
| b-NGF |  |  |  |  |
| CGRP |  |  |  |  |
| Dopamine-IgG |  |  |  |  |
| GDNF |  |  |  |  |
| LNGFR |  |  |  |  |
| NGF |  |  |  |  |
| NGF/LNGFR |  |  |  |  |
| NGF/TrKA |  |  |  |  |
| NT-3 |  |  |  |  |
| NT-3/LNGFR |  |  |  |  |
| ProNGF |  |  |  |  |
| Substance P |  |  |  |  |
| TrkA |  |  |  |  |
|  | | | | |
| Colour significance | Consistently not associated | Uncertainly not associated | Uncertainly  associated | Consistently  associated |

Table S5. Association between circulating biomarkers and OA pain depending the outcome measure of pain.

|  | Pain measures | | | | | | |
| --- | --- | --- | --- | --- | --- | --- | --- |
| Biomarkers | VAS | WOMAC | HOOS/KOOS | Other PRO | PPT | PainDetect | Other pain phenotype |
| 13KODE (B) |  | N=1, n=12 |  |  |  |  |  |
| Aβ40 (CSF) | N=1, n=26 |  |  |  |  |  |  |
| ACRP-30 (SF) | N=1, n=24 | N=1, n=24 |  |  |  |  |  |
| Acyl ornithine (B) | N=1, n=154 |  |  |  |  |  |  |
| ADAMTS5 (SF) | N=1, n=84 | N=1, n=84 |  |  |  |  | N=1, n=84 |
| Adiponectin (B) | N=5, n=1,135 | N=12, n=2,017 |  |  |  |  |  |
| Adiponectin (SF) | N=2, n=286 | N=4, n=461 |  |  |  |  | N=1, n=80 |
| Adiponectin/leptin (SF) | N=1, n=206 | N=2, n=266 |  |  |  |  |  |
| Adipsin (B) |  | N=1, n=583 |  |  |  |  |  |
| Angiopoietin-2 (B) |  | N=1, n=72 |  |  |  |  |  |
| ApoA1 (B) | N=1, n=863 | N=1, n=863 |  |  |  |  |  |
| ApoB (B) | N=1, n=863 | N=1, n=863 |  |  |  |  |  |
| ARGS (B) | N=2, n=264 |  | N=2, n=62 | N=1, n=31 |  |  |  |
| ARGS (SF) | N=1, n=31 |  | N=2, n=172 |  |  |  |  |
| ASAT (B) | N=1, n=96 |  |  |  |  |  |  |
| ATP (SF) | N=1, n=28 |  |  |  |  |  |  |
| BDNF (B) | N=2, n=55 | N=1, n=27 |  |  |  |  |  |
| BDNF (SF) | N=1, n=43 |  |  |  |  |  |  |
| BDNF (CSF) | N=2, n=55 |  |  |  |  |  |  |
| BDNF/  LNGFR (SF) | N=1, n=43 |  |  |  |  |  |  |
| b-endorphin (B) |  |  |  |  | N=1, n=40 |  |  |
| B-GNLY (B) | N=1, n=20 |  |  |  |  |  |  |
| BMP2 (B) |  | N=1, n=37 |  |  |  |  |  |
| BMP2 (SF) |  | N=1, n=37 |  |  |  |  |  |
| b-NGF (SF) | N=1, n=42 |  |  |  |  |  |  |
| Bradykinin (SF) | N=1, n=83 | N=1, n=83 |  |  |  |  | N=1, n=83 |
| C1,2C (B) |  | N=2, n=774 | N=1, n=580 |  |  |  |  |
| C1,2C (U) |  | N=2, n=774 | N=2, n=1,180 |  |  |  |  |
| C1M (B) | N=3, n=370 | N=4, n=378 | N=1, n=31 | N=1, n=31 | N=1, n=281 |  | N=2, n=385 |
| C1M (SF) | N=1, n=31 |  |  |  |  |  |  |
| C2C (B) | N=1, n=46 | N=3, n=1374 | N=3, n=1,204 | N=1, n=638 |  |  |  |
| C2C (U) |  | N=2, n=774 | N=2, n=687 |  |  |  |  |
| C2C (SF) |  | N=1, n=489 | N=1, n=38 |  |  |  |  |
| C2M (B) | N=3, n=370 | N=3, n=514 | N=1, n=31 | N=1, n=31 | N=1, n=281 |  |  |
| C2M (SF) | N=1, n=31 |  |  |  |  |  |  |
| C3A (B) | N=2, n=477 | N=2, n=477 |  |  |  |  |  |
| C3-α (B) |  |  | N=1, n=44 |  |  |  |  |
| C3-α (SF) |  |  | N=1, n=44 |  |  |  |  |
| C3-β (B) |  |  | N=1, n=44 |  |  |  |  |
| C3-β (SF) |  |  | N=1, n=44 |  |  |  |  |
| C3f (B) |  | N=1, n=415 |  |  |  |  |  |
| C3M (B) | N=3, n=370 | N=4, n=1,092 | N=1, n=31 | N=2, n=616 | N=1, n=281 |  |  |
| C3M (SF) |  |  | N=1, n=31 |  |  |  |  |
| C4M (B) |  | N=1, n=146 |  |  |  |  |  |
| C10C (B) | N=1, n=31 |  |  |  |  |  |  |
| C10C (SF) |  |  | N=1, n=31 |  |  |  |  |
| Calprotectin (B) | N=1, n=410 |  |  |  |  |  |  |
| Calprotectin (SF) |  | N=1, n=108 |  |  |  |  |  |
| Carnosine (B) | N=1, n=154 |  |  |  |  |  |  |
| Cath-K (B) |  | N=1, n=129 |  |  |  |  |  |
| CCL2 (B) |  |  |  |  | N=1, n=17 |  |  |
| CCL20 (SF) | N=1, n=99 |  |  |  |  |  |  |
| CD11c/CD206 (SF) |  |  | N=1, n=38 |  |  |  |  |
| CD14 (B) |  | N=1, n=83 | N=1, n=83 | N=1, n=410 |  |  |  |
| CGRP (B) |  | N=1, n=65 |  |  |  |  |  |
| CGRP (SF) | N=1, n=83 | N=2, n=148 |  |  |  |  | N=1, n=83 |
| CD163 (SF) | N=1, n=73 |  |  |  |  |  |  |
| CHOP (SF) | N=1, n=140 |  |  |  |  |  |  |
| Chemerin (B) | N=1, n=30 | N=2, n=613 |  |  |  |  |  |
| Chemerin (SF) |  | N=1, n=108 |  |  |  |  |  |
| CIIM (U) | N=1, n=222 | N=1, n=222 |  |  |  |  |  |
| CIR (B) |  | N=1, n=156 |  |  |  |  |  |
| CIR (SF) |  | N=1, n=156 |  |  |  |  |  |
| Classical monocyte  CCR2 (B) |  |  | N=1, n=22 |  |  |  |  |
| Clusterin (B) | N=1, n=135 |  |  |  |  |  |  |
| Cortisone (B) | N=1, n=154 |  |  |  |  |  |  |
| Coll 2-1 (B) | N=1, n=121 |  |  |  |  |  |  |
| Coll 2-1NO2 (B) | N=1, n=121 | N=3, n=1,374 | N=3, n=1,301 |  |  |  |  |
| Coll 2-1NO2 (U) |  | N=2, n=774 | N=1, n=580 |  |  |  |  |
| COMP (B) | N=6, n=1,011 | N=10, n=3,882 | N=2, n=606 | N=2, n=796 |  |  |  |
| COMP (U) | N=1, n=50 |  |  |  |  |  |  |
| COMP (SF) | N=1, n=6 |  |  |  |  |  |  |
| Cortisol (B) | N=2, n=258 |  |  |  |  |  |  |
| CPII (B) | N=1, n=46 | N=3, n=1,274 | N=2, n=1,180 | N=1, n=638 |  |  |  |
| CPII (SF) |  |  | N=1, n=38 |  |  |  |  |
| CPK (B) | N=1, n=96 |  |  |  |  |  |  |
| Creatinine (B) | N=1, n=96 |  |  |  |  |  |  |
| Creatinine (U) |  | N=1, n=580 | N=1, n=580 |  |  |  |  |
| CRP (B) | N=8, 1457 | N=9, 1,546 | N=2, n=92 | N=2, n=2,798 | N=2, n=45 |  |  |
| CRPM (B) | N=2, n=339 | N=3, n=946 |  |  | N=1, n=281 |  |  |
| CS846 (B) |  | N=3, n=1,374 | N=2, n=1,180 |  |  |  |  |
| CSF-1 (CSF) | N=1, n=40 |  | N=1, n=40 |  |  | N=1, n=38 | N=1, n=38 |
| CTX-I (B) | N=1 ; n=20 | N=5, n=1,711 | N=1, n=580 |  |  |  |  |
| CTX-I (U) | N=1, n=376 | N=4, n=2,119 | N=1, n=580 |  |  |  |  |
| CTX-I (SF) | N=1, n=70 | N=1, n=70 |  |  |  |  | N=1, n=70 |
| CTX-II (B) | N=2, n=259 | N=3, n=1,184 |  |  |  |  |  |
| CTX-II (U) | N=8, n=1,287 | N=12, n=2,386 | N=3, n=1,272 | N=8, n=2,155 |  |  |  |
| CTX-II (SF) | N=2, n=76 | N=1, n=70 | N=1, n=38 |  |  |  | N=1, n=13 |
| CU/Zn SOD (B) |  | N=1, n=29 |  |  |  |  |  |
| CX3CL1 (B) | N=1, n=26 |  |  |  |  |  |  |
| CX3CL1 (CSF) | N=2, n=66 |  | N=1, n=40 |  |  |  | N=1, n=38 |
| CXCL9 (B) |  |  |  |  | N=1, n=17 |  |  |
| CXCL10 (B) |  |  |  |  | N=1, n=17 |  |  |
| Cystine (B) | N=1, n=154 |  |  |  |  |  |  |
| DKK1 (SF) | N=1, n=40 |  |  |  |  |  |  |
| DOPA (B) | N=1, n=154 |  |  |  |  |  |  |
| Dopamine-IgG (B) | N=1, n=75 |  |  |  |  |  |  |
| Eotaxin-I (SF) |  | N=1, n=143 |  |  |  |  |  |
| ESR (B) | N=5, n=920 | N=1, n=23 |  |  |  |  |  |
| FABP2 (B) | N=1, n=410 |  |  |  |  |  |  |
| Fib 3-1 (B) |  |  |  | N=1, n=241 |  |  |  |
| Fib 3-2 (B) |  |  |  | N=1, n=241 |  |  |  |
| Fib 3-3 (B) |  |  |  | N=1, n=241 |  |  |  |
| FGF-21 (B) | N=1, n=127 |  |  |  |  |  |  |
| Flt-1 (CSF) | N=1, n=52 |  |  |  | N=2, n=67 |  |  |
| GAG (SF) | N=1, n=6 |  |  |  |  |  |  |
| Galectin3 (B) | N=1, n=272 |  |  |  |  |  |  |
| GDNF (B) | N=1, n=20 |  |  |  |  |  |  |
| GDNF (CSF) | N=1, n=20 |  |  |  | N=1, n=20 |  |  |
| Ghrelin (B) |  | N=1, n=146 |  |  |  |  |  |
| Ghrelin (SF) | N=2, n=149 |  |  |  |  |  |  |
| GLCAS (B) | N=1, n=154 |  |  |  |  |  |  |
| Glc-Gal-PYD (U) |  | N=1, n=67 |  |  |  |  |  |
| Glycemia =fasting  blood glucose (B) |  |  | N=1, n=297 |  |  |  |  |
| GM-CSF (B) |  | N=1, n=29 |  |  |  |  |  |
| GRP78 (SF) | N=1, n=140 |  |  |  |  |  |  |
| Hexacetonid  acid (SF) |  |  |  | N=1, n=100 |  |  |  |
| HbA1C (B) |  |  | N=1, n=70 |  |  |  |  |
| HDL cholesterol (B) | N=1, n=48 |  |  |  |  |  |  |
| HGF (CSF) | N=1, n=40 |  | N=1, n=40 |  |  |  |  |
| HMGB1 (SF) | N=1, n=30 |  |  |  |  |  |  |
| hsCRP (B) | N=10, 3,113 | N=9, n=2,340 | N=1, n=281 | N=2, n=1,412 | N=1, n=281 |  | N=1, n=281 |
| hsCRP (SF) |  | N=1, n=115 |  |  |  |  |  |
| Hyaluronic acid (B) | N=6, n=1,423 | N=7, n=1,709 | N=3, n=1,796 | N=1, n=638 |  |  |  |
| Hyaluronic acid (SF) |  |  | N=1, n=38 |  |  |  |  |
| Triglyceride (B) | N=3, n=264 | N=1, n=170 | N=2, n=240 |  |  |  |  |
| ICAM-1 (B) |  |  |  | N=1, n=100 |  |  |  |
| ICAM-1 (SF) |  |  |  |  |  |  | N=1, n=25 |
| ICAM-1 (CSF) | N=1, n=52 |  |  |  | N=1, n=52 |  |  |
| IGF-I (B) | N=1, n=30 |  |  |  |  |  |  |
| IGF-I (SF) | N=1, n=30 |  |  |  |  |  |  |
| IL1 (B) | N=9, n=463 | N=3, n=297 |  | N=1, n=24 |  |  |  |
| IL1 (SF) | N=2, n=153 | N=3, n=386 |  |  |  |  | N=2, n=153 |
| IL1 (CSF) | N=1, n=26 |  |  |  |  |  |  |
| IL1-R (B) | N=1, n=42 | N=2, n=303 |  |  |  |  |  |
| IL1-R (SF) | N=1, n=6 |  |  |  |  |  |  |
| IL1-R (CSF) | N=1, n=50 |  |  |  |  |  |  |
| IL2 (B) |  | N=1, n=29 |  |  |  |  |  |
| IL2-R (B) |  | N=1, n=46 |  |  |  |  |  |
| IL4 (B) |  | N=1, n=29 |  |  |  |  |  |
| IL4 (SF) | N=1, n=41 |  |  |  |  |  |  |
| IL4-R (B) |  | N=1, n=29 |  |  |  |  |  |
| IL5 (B) |  | N=1, n=29 |  |  |  |  |  |
| IL6 (B) | N=13, n=1,099 | N=11, n=1,410 | N=4, n=263 | N=1, n=23 | N=3, n=87 |  | N=2, n=202 |
| IL6 (SF) | N=10, n=723 | N=6, n=688 | N=2, n=243 |  | N=1, n=40 |  | N=2, n=202 |
| IL6 (CSF) | N=4, n=194 |  |  |  | N=1, n=40 |  | N=1, n=98 |
| IL6-R (B) | N=1, n=50 | N=2, n=303 |  |  |  |  |  |
| IL6-R (CSF) | N=1, n=50 |  |  |  |  |  |  |
| IL7 (B) |  | N=1, n=29 |  |  |  |  |  |
| IL7 (SF) | N=1, n=34 |  |  |  |  |  |  |
| IL8 (B) | N=5, n=235 | N=4, n=826 | N=1, n=115 |  | N=4, n=107 |  |  |
| IL8 (SF) | N=5, n=246 | N=1, n=70 | N=4, n=358 | N=1, n=69 | N=1, n=40 |  | N=1, n=70 |
| IL8 (CSF) | N=3, n=89 |  |  |  | N=1, n=40 | N=1, n=38 |  |
| IL10 (B) | N=5, n=398 | N=4, n=479 | N=2, n=72 |  | N=1, n=54 |  |  |
| IL10 (SF) | N=3, n=99 | N=1, n=24 |  |  |  |  |  |
| IL10 (CSF) | N=1, n=20 |  |  |  |  |  |  |
| IL12 (B) |  | N=1, n=29 |  |  |  |  |  |
| IL12 (SF) | N=1, n=34 |  |  |  |  |  |  |
| IL13 (B) |  | N=1, n=29 |  |  |  |  |  |
| IL13 (SF) | N=1, n=34 |  |  |  |  |  |  |
| IL15 (B) |  | N=1, n=226 |  |  |  |  |  |
| IL15 (CSF) | N=1, n=52 |  |  |  |  |  |  |
| IL17 (B) | N=2, n=104 | N=2, n=201 |  | N=1, n=70 |  |  |  |
| IL17 (SF) | N=1, n=70 | N=2, n=296 |  | N=1, n=70 |  |  |  |
| IL21 (B) |  | N=1, n=131 |  |  |  |  |  |
| IL22 (B) |  | N=1, n=46 |  |  |  |  |  |
| IL23 (B) |  | N=1, n=131 |  |  |  |  |  |
| IL23 (SF) | N=1, n=5 |  |  |  |  |  |  |
| IL25 (B) | N=1, n=34 |  |  |  |  |  |  |
| IL38 (B) | N=1, n=23 |  |  |  |  |  |  |
| INFgamma (B) |  | N=1, n=29 |  |  |  |  |  |
| INFgamma (SF) | N=1, n=34 |  |  |  |  |  |  |
| Insulin resistance (B) | N=1, n=400 |  |  |  |  |  |  |
| Int monocyte  CCR2 (B) |  |  | N=1, n=22 |  |  |  |  |
| Int monocyte  HLA-DR (B) |  |  | N=1, n=22 |  |  |  |  |
| IP-10 (B) |  |  |  |  | N=1, n=15 |  |  |
| IP-10 (CSF) |  |  |  |  | N=1, n=52 |  |  |
| LBP (B) |  | N=1, n=25 |  | N=1, n=410 |  |  |  |
| LBP (SF) |  | N=1, n=25 |  |  |  |  |  |
| LC3A (B) | N=1, n=165 |  |  |  |  |  |  |
| LDH (B) | N=1, n=96 |  |  |  |  |  |  |
| LDL cholesterol (B) | N=1, n=48 |  |  |  |  |  |  |
| Leptin (B) | N=8, n=850 | N=11, n=1,663 |  |  |  |  | N=1, n=98 |
| Leptin (SF) | N=5, n=853 | N=7, n=1,045 |  |  |  |  | N=2, n=158 |
| Leptin (CSF) | N=1, n=98 |  |  |  |  |  | N=1, n=98 |
| Leptin/adiponectin (B) | N=1, n=83 | N=1, n=863 |  | N=1, n=284 |  |  |  |
| Leptin/adiponectin (SF) |  | N=1, n=115 |  |  |  |  |  |
| LIF-R (CSF) | N=1, n=40 |  | N=1, n=40 |  |  |  |  |
| LNGFR (SF) | N=1, n=43 |  |  |  |  |  |  |
| LPS (B) |  | N=1, n=25 |  |  |  |  |  |
| LPS (SF) |  | N=1, n=25 |  |  |  |  |  |
| Lubricin (SF) |  |  |  |  |  |  | N=1, n=61 |
| LTα (B) | N=1, n=50 |  |  |  |  |  |  |
| MCP1 (B) | N=1, n=26 | N=2, n=744 |  |  |  |  |  |
| MCP1 (SF) | N=1, n=40 | N=1, n=161 |  |  | N=1, n=40 |  |  |
| MCP1 (CSF) | N=2, n=66 |  |  |  | N=1, n=40 |  |  |
| M-CSF (SF) | N=1, n=34 | N=1, n=83 |  |  |  |  | N=1, n=83 |
| MDA (SF) | N=1, n=40 |  |  |  |  |  |  |
| MIF (B) |  | N=1, n=226 |  |  |  |  |  |
| MIF (SF) |  | N=1, n=226 |  |  |  |  |  |
| miR300 (B) |  | N=1, n=74 |  |  |  |  |  |
| MMP1 (B) | N=1, n=376 |  |  |  |  |  |  |
| MMP1 (SF) | N=2, n=11 |  |  |  |  |  |  |
| MMP2 (SF) | N=1, n=5 |  |  |  |  |  |  |
| MMP3 (B) | N=1, n=376 | N=4, n=984 | N=2, n=604 |  |  |  |  |
| MMP3 (SF) | N=3, n=317 | N=1, n=83 |  |  |  |  | N=2, n=108 |
| MMP8 (SF) | N=1, n=5 |  |  |  |  |  |  |
| MMP9 (SF) | N=1, n=228 |  |  |  |  |  |  |
| MMP13 (B) |  | N=1, n=37 |  |  |  |  |  |
| MMP13 (SF) | N=3, n=316 | N=1, n=83 |  |  |  |  | N=1, n=83 |
| MMP14 (SF) | N=1, n=228 |  |  |  |  |  |  |
| MnSOD (B) |  | N=1, n=29 |  |  |  |  |  |
| Monocyte CD16 (B) |  |  | N=1, n=22 |  |  |  |  |
| mTOR (B) | N=1, n=50 | N=1, n=50 |  |  |  |  |  |
| MUFA (B) |  |  | N=1, n=533 |  |  |  |  |
| Myoglobin (B) | N=1, n=96 |  |  |  |  |  |  |
| Neuropeptide Y (SF) | N=1, n=83 |  |  | N=1, n=100 |  |  |  |
| NGF (SF) | N=1, n=34 | N=1, n=23 |  |  |  | N=1, n=23 |  |
| NGF/TrKA (SF) | N=1, n=43 |  |  |  |  |  |  |
| NO (B) | N=1, n=137 |  |  |  |  |  |  |
| NO (SF) |  |  |  |  |  |  | N=1, n=10 |
| NTXI (B) | N=1, n=96 | N=2, n=774 | N=1, n=580 |  |  |  |  |
| NTXI (U) | N=1, n=46 | N=2, n=774 | N=1, n=580 |  |  |  |  |
| Number Treg (B) | N=2, n=64 | N=1, n=18 |  |  |  |  |  |
| Number Treg (SF) | N=1, n=46 |  |  |  |  |  |  |
| NT-3 (SF) | N=1, n=43 |  |  |  |  |  |  |
| NT-3/LNGFR (SF) | N=1, n=43 |  |  |  |  |  |  |
| OC total (B) |  | N=3, n=2,311 |  |  |  |  |  |
| Omega-3 PUFA (B) |  |  | N=1, n=533 |  |  |  |  |
| Omega-6 PUFA (B) |  |  | N=1, n=533 |  |  |  |  |
| Omega-6/omega-3  PUFA (B) |  | N=1, n=167 |  |  |  |  |  |
| Omentin (B) |  | N=1, n=263 |  |  |  |  |  |
| Omentin (SF) |  | N=2, n=378 | N=1, n=168 |  |  |  |  |
| Osteopontin (B) | N=1, n=60 |  |  |  |  |  |  |
| Osteopontin (SF) | N=1, n=60 | N=1, n=115 | N=1, n=168 |  |  |  |  |
| Osteoprotegerin (SF) | N=1, n=5 |  |  |  |  |  |  |
| PACAP (SF) | N=1, n=101 |  |  |  |  |  |  |
| PDGF (B) | N=1, n=38 |  |  |  |  |  |  |
| PEA (B) | N=1, n=154 |  |  |  |  |  |  |
| PGE2 (B) | N=1, n=96 | N=1, n=12 |  |  |  |  |  |
| PGE2 (SF) | N=1, n=96 |  | N=1, n=70 |  |  |  |  |
| PIIANP (B) | N=1, n=96 | N=5, n=1,487 | N=2, n=1,180 |  |  |  |  |
| PIICP (B) | N=1, n=174 |  |  |  |  |  |  |
| PIIINP (B) | N=1, n=376 | N=1, n=67 |  |  |  |  |  |
| PIGF (CSF) | N=1, n=52 |  |  |  | N=1, n=52 |  |  |
| PINP (B) | N=1, n=376 | N=1, n=1,002 |  | N=1, n=1,002 |  |  |  |
| PRO-C1 (B) |  | N=1, n=146 |  |  |  |  |  |
| PRO-C2 (B) |  | N=1, n=146 |  |  |  |  |  |
| PRO-C3 (B) |  | N=1, n=146 |  |  |  |  |  |
| PRO-C4 (B) |  | N=1, n=146 |  |  |  |  |  |
| ProNGF (SF) | N=1, n=43 |  |  |  |  |  |  |
| ProNGF/LNGFR (SF) | N=1, n=43 |  |  |  |  |  |  |
| RANKL (SF) | N=1, n=5 |  |  |  |  |  |  |
| RANKL/OPG (SF) | N=1, n=5 |  |  |  |  |  |  |
| Resistin (B) | N=3, n=212 | N=8, n=791 |  |  |  |  |  |
| Resistin (SF) | N=2, n=286 | N=5, n=475 |  |  |  |  |  |
| S100A8/S100A9 (B) |  | N=1, n=141 |  |  |  |  |  |
| SCF (CSF) | N=1, n=40 |  | N=1, n=40 |  |  |  |  |
| SFA (B) |  |  | N=1, n=533 |  |  |  |  |
| SCGF-beta (SF) | N=1, n=34 |  |  |  |  |  |  |
| SPLI (CSF) | N=1, n=11 |  |  |  |  |  |  |
| Substance P (SF) | N=1, n=83 | N=1, n=83 |  |  |  |  | N=1, n=83 |
| Succinic acid (B) | N=1, n=154 |  |  |  |  |  |  |
| SOD (SF) | N=1, n=30 |  |  |  |  |  |  |
| T2GM (B) |  |  | N=1, n=31 |  |  |  |  |
| TAC (SF) | N=2, n=70 |  |  |  |  |  |  |
| TGF beta (B) | N=4, n=154 |  |  |  |  |  |  |
| TGF beta (CSF) | N=2, n=55 |  |  |  |  |  |  |
| TIMP (B) | N=1, n=63 |  |  |  |  |  |  |
| TIMP (SF) | N=1, n=6 |  |  |  |  |  |  |
| TIMP-1 (SF) |  |  |  |  |  |  | N=1, n=25 |
| TNF alpha (B) | N=10, n=504 | N=10, n=1,158 | N=1, n=22 | N=1, n=100 |  | N=2, n=75 | N=1, n=98 |
| TNF alpha (U) | N=1, n=50 |  |  |  |  |  |  |
| TNF alpha (SF) | N=10, n=535 | N=4, n=209 | N=2, n=241 |  |  |  | N=3, n=168 |
| TNF alpha (CSF) | N=4, n=173 |  |  |  |  |  | N=1, n=98 |
| TNF alpha-R1 (B) | N=1, n=50 | N=4, n=376 |  |  |  |  |  |
| TNF alpha-R1 (CSF) | N=1, n=50 |  |  |  |  |  |  |
| TNF alpha-R2 (B) | N=1, n=50 | N=4, n=376 |  |  |  |  |  |
| TNF alpha-R2 (CSF) | N=1, n=50 |  |  |  |  |  |  |
| Total cholesterol (B) | N=3, n=277 |  |  |  |  |  |  |
| Total Fatty Acid (B) |  |  | N=1, n=533 |  |  |  |  |
| Total PUFA (B) |  |  | N=1, n=533 |  |  |  |  |
| TrkA (SF) | N=1, n=43 |  |  |  |  |  |  |
| TSG-6 (SF) | N=1, n=6 |  |  |  |  |  |  |
| uPA (SF) | N=1, n=228 |  |  |  |  |  |  |
| Uric acid (B) | N=2, n=228 |  | N=1, n=70 |  |  |  |  |
| Uric acid (SF) |  |  |  | N=1, n=69 |  |  |  |
| VCAM-1 (SF) |  |  |  |  |  |  | N=1, n=25 |
| VCAM-1 (CSF) | N=1, n=52 |  |  |  | N=1, n=52 |  |  |
| VEGF (B) | N=2, n=55 |  |  |  |  |  |  |
| VEGF (SF) | N=1, n=34 |  |  |  |  |  | N=1, n=34 |
| VEGF (CSF) | N=2, n=92 |  | N=1, n=40 |  | N=1, n=52 |  |  |
| VEGFA (CSF) | N=1, n=40 |  | N=1, n=40 |  |  |  |  |
| Visfatin (B) | N=2, n=912 | N=4, n=1,645 |  |  |  |  |  |
| Visfatin (SF) | N=1, n=206 | N=2, n=321 |  |  |  |  |  |
| Vitamin D (B) | N=1, n=90 | N=2, n=263 |  | N=1, n=787 |  |  |  |
| TWEAK (CSF) | N=1, n=40 |  | N=1, n=40 |  |  |  |  |
| YKL-40 (B) | N=2, n=416 | N=3, n=251 |  |  |  |  |  |
| YKL-40 (SF) |  | N=1, n=144 |  |  |  |  |  |
| ZRP (B) | N=1, n=410 |  |  |  |  |  |  |

(B)= blood ; (CSF)= cerebrospinal fluid ; (SF)= synovial fluid ; (U)=urine

N= number of included studies; n=number of OA patients

| Colour significance | Consistently not associated | Uncertainly not associated | Uncertainly  associated | Consistently  associated |
| --- | --- | --- | --- | --- |

Table S6. Assessment of the methodological quality of the included studies. (Abstracts were not assessed).

| Authors |  | Journal if duplicate in authors | Q1 | Q2 | Q3 | Q4 | Q5 | Q6 | Q7 | Q8 | Q9 |
| --- | --- | --- | --- | --- | --- | --- | --- | --- | --- | --- | --- |
| Abassifard | C |  |  |  |  |  |  |  |  |  |  |
| Abd Elazeem | C |  |  |  |  |  |  |  |  |  |  |
| Abd Elghany | C |  |  |  |  |  |  |  |  |  |  |
| Ahn | CS |  |  |  |  |  |  |  |  |  |  |
| Alekseeva | Abstract | | | | | | | | | | |
| Alexander | CS |  |  |  |  |  |  |  |  |  |  |
| Altaie | CS |  |  |  |  |  |  |  |  |  |  |
| Arendt-Nielsen | CS |  |  |  |  |  |  |  |  |  |  |
| Askari | C | PLoS One 2016 ;11 :e0164757 |  |  |  |  |  |  |  |  |  |
| Askari | C | Endocrine Regul 2020 ; 54 :6 |  |  |  |  |  |  |  |  |  |
| Aslam | CS |  |  |  |  |  |  |  |  |  |  |
| Attur | Co | Arthritis Rheum 2011 |  |  |  |  |  |  |  |  |  |
| Attur | Abstract. Arthritis Care Res. 2011 | | | | | | | | | | |
| Awadallah | C |  |  |  |  |  |  |  |  |  |  |
| Azim | Co |  |  |  |  |  |  |  |  |  |  |
| Barker | CS |  |  |  |  |  |  |  |  |  |  |
| Barman | Abstract | | | | | | | | | | |
| Bas | CS |  |  |  |  |  |  |  |  |  |  |
| Bay-Jensen | C | Osteoarthr Cartil Open 2023 |  |  |  |  |  |  |  |  |  |
| Bay-Jensen | CS | Clinical Biochemistry 2018 |  |  |  |  |  |  |  |  |  |
| Bay-Jensen | Abstract. Osteoarthr Cart 2016 | | | | | | | | | | |
| Bay-Jensen | Abstract. Osteoarthr Cart 2015 | | | | | | | | | | |
| Bay-Jensen | Abstract. Osteoarthr Cart 2022 | | | | | | | | | | |
| Bihlet | CS | Arthritis Res Ther 2019 |  |  |  |  |  |  |  |  |  |
| Bihlet | Abstract. Ann Rheum Dis 2015 | | | | | | | | | | |
| Binvignat | CS | BioRxiv 2025 |  |  |  |  |  |  |  |  |  |
| Binvignat | Co | Osteoarthr Cart 2025 |  |  |  |  |  |  |  |  |  |
| Bjurström | Co | Pain Practice. 2022 ;22 :66 |  |  |  |  |  |  |  |  |  |
| Blichfeldt-Eckhardt | Co |  |  |  |  |  |  |  |  |  |  |
| Cafferata | CS |  |  |  |  |  |  |  |  |  |  |
| Calvet | Abstract. Ann Rheum Dis 2018 | | | | | | | | | | |
| Calvet | CS | Osteoarthr Cart 2018 |  |  |  |  |  |  |  |  |  |
| Calvet | Co | Arthritis Res Ther 2024 |  |  |  |  |  |  |  |  |  |
| Chen | CS |  |  |  |  |  |  |  |  |  |  |
| Cheng | CS |  |  |  |  |  |  |  |  |  |  |
| Chiba | Co |  |  |  |  |  |  |  |  |  |  |
| Chong | CS |  |  |  |  |  |  |  |  |  |  |
| Cioroianu | CS |  |  |  |  |  |  |  |  |  |  |
| Colombini | Co |  |  |  |  |  |  |  |  |  |  |
| Connelly | Abstract | | | | | | | | | | |
| Costello | Abstract | | | | | | | | | | |
| Dam | Co |  |  |  |  |  |  |  |  |  |  |
| David Ho | CS |  |  |  |  |  |  |  |  |  |  |
| de Jong | Abstract | | | | | | | | | | |
| Dinç | Co |  |  |  |  |  |  |  |  |  |  |
| Ding | Abstract | | | | | | | | | | |
| Dong | C | Intern Orthop 2015 ;39 :1237 |  |  |  |  |  |  |  |  |  |
| Dong | CS | Intern Orthop 2018 ;42 :1283 |  |  |  |  |  |  |  |  |  |
| Dorleijn | Co |  |  |  |  |  |  |  |  |  |  |
| Dündar | CS |  |  |  |  |  |  |  |  |  |  |
| Eathakkattu | Abstract | | | | | | | | | | |
| Eitner | CS |  |  |  |  |  |  |  |  |  |  |
| Ellabban | C |  |  |  |  |  |  |  |  |  |  |
| Ellaithy | C |  |  |  |  |  |  |  |  |  |  |
| Elnemr | C |  |  |  |  |  |  |  |  |  |  |
| Erden | CS |  |  |  |  |  |  |  |  |  |  |
| Esmayil | CS |  |  |  |  |  |  |  |  |  |  |
| Farinelli | C |  |  |  |  |  |  |  |  |  |  |
| Finckh | Abstract | | | | | | | | | | |
| Fioravanti | CS |  |  |  |  |  |  |  |  |  |  |
| Flores Bjurström | C | Pain 2020 |  |  |  |  |  |  |  |  |  |
| Gaballah | CS |  |  |  |  |  |  |  |  |  |  |
| Galvez | Co |  |  |  |  |  |  |  |  |  |  |
| Gandhi | CS |  |  |  |  |  |  |  |  |  |  |
| García‑Manrique | CS |  |  |  |  |  |  |  |  |  |  |
| Garnero | C | Ann Rheum Dis 2001 |  |  |  |  |  |  |  |  |  |
| Garnero | CS | J Rheumatol 2005 |  |  |  |  |  |  |  |  |  |
| Georgiev | C |  |  |  |  |  |  |  |  |  |  |
| Giordano | C |  |  |  |  |  |  |  |  |  |  |
| Gloersen | Abstract | | | | | | | | | | |
| Gómez-Aristizábal | Co |  |  |  |  |  |  |  |  |  |  |
| Guan | C | Clin Lab 2015 |  |  |  |  |  |  |  |  |  |
| Guan | C | J Muscul Neuro Interact 2019 |  |  |  |  |  |  |  |  |  |
| Hafez | Abstract | | | | | | | | | | |
| Haraden | CS |  |  |  |  |  |  |  |  |  |  |
| Harsanyi | Abstract | | | | | | | | | | |
| Hefferman | C |  |  |  |  |  |  |  |  |  |  |
| Herrero-Manley | C |  |  |  |  |  |  |  |  |  |  |
| Hick | Co |  |  |  |  |  |  |  |  |  |  |
| Ho | Abstract | | | | | | | | | | |
| Huang | Abstract | | | | | | | | | | |
| Huebner | CS | Osteoarthr Cart 2016 |  |  |  |  |  |  |  |  |  |
| Huebner | Abstract. Osteoarthr Cart 2018 | | | | | | | | | | |
| Ilia | CS |  |  |  |  |  |  |  |  |  |  |
| Imamura | C |  |  |  |  |  |  |  |  |  |  |
| Inoue | Co |  |  |  |  |  |  |  |  |  |  |
| Ishijima | CS |  |  |  |  |  |  |  |  |  |  |
| Ismail S | Abstract | | | | | | | | | | |
| Jiang | C |  |  |  |  |  |  |  |  |  |  |
| Jurewicz | CS |  |  |  |  |  |  |  |  |  |  |
| Kalai | Abstract | | | | | | | | | | |
| Kalogera | CS |  |  |  |  |  |  |  |  |  |  |
| Kamel | C |  |  |  |  |  |  |  |  |  |  |
| Kamiab | C |  |  |  |  |  |  |  |  |  |  |
| Kang | CS |  |  |  |  |  |  |  |  |  |  |
| Kapetanakis | C |  |  |  |  |  |  |  |  |  |  |
| Karimov | Abstract | | | | | | | | | | |
| Kato | CS |  |  |  |  |  |  |  |  |  |  |
| Keenan | CS |  |  |  |  |  |  |  |  |  |  |
| Kim | CS |  |  |  |  |  |  |  |  |  |  |
| Kittelson | Abstract | | | | | | | | | | |
| Klein-Wieringa | CS |  |  |  |  |  |  |  |  |  |  |
| Klocke | CS |  |  |  |  |  |  |  |  |  |  |
| Kluzek | Abstract | | | | | | | | | | |
| Kosek | C |  |  |  |  |  |  |  |  |  |  |
| Kraus | C |  |  |  |  |  |  |  |  |  |  |
| Kropáčková | C |  |  |  |  |  |  |  |  |  |  |
| Kumahashi | CS |  |  |  |  |  |  |  |  |  |  |
| Lambova | C |  |  |  |  |  |  |  |  |  |  |
| Larsson | C | Osteoarthr Cart 2015 |  |  |  |  |  |  |  |  |  |
| Larsson | CS | Osteoarthr Cart 2012 |  |  |  |  |  |  |  |  |  |
| Larsson | CS | Osteoarthr Cart 2024 |  |  |  |  |  |  |  |  |  |
| Laskarin | Abstract | | | | | | | | | | |
| Lee | C | Arthritis Care Res 2011 |  |  |  |  |  |  |  |  |  |
| Lee | CS | Int J Vitam Nutr Res 2023 |  |  |  |  |  |  |  |  |  |
| Lei | CS |  |  |  |  |  |  |  |  |  |  |
| Leung | CS |  |  |  |  |  |  |  |  |  |  |
| Levinger | Abstract | | | | | | | | | | |
| Li | C | Scand J Clin Lab Invest 2012 |  |  |  |  |  |  |  |  |  |
| Li | C | Ann Clin Biochemistry 2015 |  |  |  |  |  |  |  |  |  |
| Li | C | Cartilage 2019 |  |  |  |  |  |  |  |  |  |
| Li | CS | BMC Musculoskelet Dis 2020 |  |  |  |  |  |  |  |  |  |
| Liem | Co | Biomark Med 2022 ;16 :633 |  |  |  |  |  |  |  |  |  |
| Liem | Co | Sci Rep 2020 ;10 :11328 |  |  |  |  |  |  |  |  |  |
| Liem | Abstract. Arthritis Rheum 2022 | | | | | | | | | | |
| Lindh | C |  |  |  |  |  |  |  |  |  |  |
| Liu | CS | Plos One 2022 |  |  |  |  |  |  |  |  |  |
| Liu | Abstract. Osteoarthr Cart 2014 | | | | | | | | | | |
| Liu | C | Med Sci Monit 2015 ;21 :363 |  |  |  |  |  |  |  |  |  |
| Liu | C | J App Biomed 2024 |  |  |  |  |  |  |  |  |  |
| Loef | Co |  |  |  |  |  |  |  |  |  |  |
| Loukov | C |  |  |  |  |  |  |  |  |  |  |
| Lubbeke | Abstract | | | | | | | | | | |
| Lubbeke | CS | Int Orthop 2013 |  |  |  |  |  |  |  |  |  |
| Lundborg | CS |  |  |  |  |  |  |  |  |  |  |
| Luo | Co |  |  |  |  |  |  |  |  |  |  |
| Marouf | CS |  |  |  |  |  |  |  |  |  |  |
| Martel-Pelletier | CS |  |  |  |  |  |  |  |  |  |  |
| Massengale | Abstract | | | | | | | | | | |
| Mehta | Co |  |  |  |  |  |  |  |  |  |  |
| Messier | CS |  |  |  |  |  |  |  |  |  |  |
| Miller | Abstract | | | | | | | | | | |
| Mishra | C |  |  |  |  |  |  |  |  |  |  |
| Mohammed | C |  |  |  |  |  |  |  |  |  |  |
| Mohasseb | C | Mediterr J Rheumatol 2019 |  |  |  |  |  |  |  |  |  |
| Mohasseb | Abstract. Osteoporos Int 2014 | | | | | | | | | | |
| Moraes | Abstract | | | | | | | | | | |
| Mukundan | Abstract | | | | | | | | | | |
| Mundermann | CS |  |  |  |  |  |  |  |  |  |  |
| Muraki | Abstract | | | | | | | | | | |
| Najirman | Abstract | | | | | | | | | | |
| Nees | CS | Biomedicines 2022 |  |  |  |  |  |  |  |  |  |
| Nees | CS | J Clin Med 2020 |  |  |  |  |  |  |  |  |  |
| Nees | CS | J Clin Med 2019 |  |  |  |  |  |  |  |  |  |
| Neogi | Abstract | | | | | | | | | | |
| Nongmaithem | CS |  |  |  |  |  |  |  |  |  |  |
| Nwosu | C |  |  |  |  |  |  |  |  |  |  |
| Ogawa | CS |  |  |  |  |  |  |  |  |  |  |
| Ohashi | CS |  |  |  |  |  |  |  |  |  |  |
| Oikonomidis | CS |  |  |  |  |  |  |  |  |  |  |
| Oliinyk | Abstract. Osteoarthr Cart 2020 | | | | | | | | | | |
| Oliinyk | Abstract. Osteoarthr Cart 2016 | | | | | | | | | | |
| Orellana | CS |  |  |  |  |  |  |  |  |  |  |
| Orita | CS |  |  |  |  |  |  |  |  |  |  |
| Palada | C |  |  |  |  |  |  |  |  |  |  |
| Pan | Co |  |  |  |  |  |  |  |  |  |  |
| Papaneophytou | C |  |  |  |  |  |  |  |  |  |  |
| Peeler | Abstract | | | | | | | | | | |
| Pelletier | Abstract | | | | | | | | | | |
| Penninx | CS |  |  |  |  |  |  |  |  |  |  |
| Perruccio | CS |  |  |  |  |  |  |  |  |  |  |
| Pers | CS |  |  |  |  |  |  |  |  |  |  |
| Petersen | C |  |  |  |  |  |  |  |  |  |  |
| Philothra | CS |  |  |  |  |  |  |  |  |  |  |
| Pustjens | Abstract | | | | | | | | | | |
| Puts | CS |  |  |  |  |  |  |  |  |  |  |
| Radojcic | Co |  |  |  |  |  |  |  |  |  |  |
| Reijman | CS |  |  |  |  |  |  |  |  |  |  |
| Ren | C |  |  |  |  |  |  |  |  |  |  |
| Richette | C |  |  |  |  |  |  |  |  |  |  |
| Riegger | CS |  |  |  |  |  |  |  |  |  |  |
| Rotterud | CS |  |  |  |  |  |  |  |  |  |  |
| Ruan | CS | Clin Rheum 2019 |  |  |  |  |  |  |  |  |  |
| Ruan | CS | Osteoarthr Cart 2019 |  |  |  |  |  |  |  |  |  |
| Runhaar | Co |  |  |  |  |  |  |  |  |  |  |
| Saengsiwaritt | Co |  |  |  |  |  |  |  |  |  |  |
| Saich | Abstract | | | | | | | | | | |
| Sandhu | Co |  |  |  |  |  |  |  |  |  |  |
| Sato | CS |  |  |  |  |  |  |  |  |  |  |
| Savitskaya | Abstract | | | | | | | | | | |
| Schutte | CS |  |  |  |  |  |  |  |  |  |  |
| Selistre | CS |  |  |  |  |  |  |  |  |  |  |
| Sellam | Co | Semin Arthritis Rheum 2021 |  |  |  |  |  |  |  |  |  |
| Sellam | Abstract. Ann Rheum Dis 2014 | | | | | | | | | | |
| Shao | C |  |  |  |  |  |  |  |  |  |  |
| Sharma | C |  |  |  |  |  |  |  |  |  |  |
| Shen | Co |  |  |  |  |  |  |  |  |  |  |
| Shibata | CS |  |  |  |  |  |  |  |  |  |  |
| Shimura | C | Osteoarthr Cart 2013 |  |  |  |  |  |  |  |  |  |
| Shimura | Abstract. Osteoarthr Cart 2018 | | | | | | | | | | |
| Si | CS |  |  |  |  |  |  |  |  |  |  |
| Sibille | CS |  |  |  |  |  |  |  |  |  |  |
| Siebuhr | Abstract | | | | | | | | | | |
| Simao | C |  |  |  |  |  |  |  |  |  |  |
| Singh | Abstract | | | | | | | | | | |
| Solignac | Abstract | | | | | | | | | | |
| Song | C |  |  |  |  |  |  |  |  |  |  |
| Sowers | Co |  |  |  |  |  |  |  |  |  |  |
| Srivastava | Abstract. Osteoarthr Cart 2014 | | | | | | | | | | |
| Srivastava | Abstract. Osteoarthr Cart 2015 | | | | | | | | | | |
| Stabler | Abstract | | | | | | | | | | |
| Stannus | Abstract | | | | | | | | | | |
| Strebkova | Abstract. Ann Rheum Dis 2017 | | | | | | | | | | |
| Strebkova | Abstract. Osteoporos Int 2022 | | | | | | | | | | |
| Sturmer | CS |  |  |  |  |  |  |  |  |  |  |
| Sun | C | Disease Markers 2013 |  |  |  |  |  |  |  |  |  |
| Sun | CS | Innate Immun 2019 |  |  |  |  |  |  |  |  |  |
| Takahashi | C |  |  |  |  |  |  |  |  |  |  |
| Tamm | Abstract | | | | | | | | | | |
| Tarasovs | Co |  |  |  |  |  |  |  |  |  |  |
| Taskina | Abstract. Aging Clin. Exp. Res 2023 S244 | | | | | | | | | | |
| Taskina | Abstract. Aging Clin. Exp. Res 2023 S484 | | | | | | | | | | |
| Tay | Abstract | | | | | | | | | | |
| Thudium | Abstract | | | | | | | | | | |
| Trifonova | Abstract | | | | | | | | | | |
| Tsuchiya | CS |  |  |  |  |  |  |  |  |  |  |
| Turan | C |  |  |  |  |  |  |  |  |  |  |
| Udomsinprasert | C |  |  |  |  |  |  |  |  |  |  |
| Valdes | Co | Arthritis Care Res 2011 ; |  |  |  |  |  |  |  |  |  |
| Valdes | Co | Ann Rheum Dis 2011 |  |  |  |  |  |  |  |  |  |
| van Berkel | Co |  |  |  |  |  |  |  |  |  |  |
| van Helvoort | CS |  |  |  |  |  |  |  |  |  |  |
| van Meurs | Co |  |  |  |  |  |  |  |  |  |  |
| Verma | CS |  |  |  |  |  |  |  |  |  |  |
| Villanova Lopez | Abstract | | | | | | | | | | |
| Vincent | CS | Open Orthop J 2013 |  |  |  |  |  |  |  |  |  |
| Vincent | Abstract. Osteoarthr Cart 2018 | | | | | | | | | | |
| Waluyo | CS |  |  |  |  |  |  |  |  |  |  |
| Wang | C | Am J Transl Res 2022 |  |  |  |  |  |  |  |  |  |
| Wang | C | World J Clin Cases 2019 |  |  |  |  |  |  |  |  |  |
| Wang | C | Journal of Jilin University Medicine Edition 2014 |  |  |  |  |  |  |  |  |  |
| Wang L | Abstact. Eur J Immunol 2019 | | | | | | | | | | |
| Wang L | Abstract. Neuropeptides 2016 | | | | | | | | | | |
| Wang Z | Co | Arthritis Res Ther 2023 |  |  |  |  |  |  |  |  |  |
| Warner | CS | Front Immunol 2020 |  |  |  |  |  |  |  |  |  |
| Warner | Co | Eur J Pain 2017 |  |  |  |  |  |  |  |  |  |
| Wen | C |  |  |  |  |  |  |  |  |  |  |
| Wislowska | CS |  |  |  |  |  |  |  |  |  |  |
| Wolfe | CS |  |  |  |  |  |  |  |  |  |  |
| Wu | CS |  |  |  |  |  |  |  |  |  |  |
| Xie | Abstract | | | | | | | | | | |
| Xin | C |  |  |  |  |  |  |  |  |  |  |
| Yan | C |  |  |  |  |  |  |  |  |  |  |
| Yang | CS |  |  |  |  |  |  |  |  |  |  |
| Yokohama | Abstract | | | | | | | | | | |
| Yu | CS | Clin Chim Acta 2017 |  |  |  |  |  |  |  |  |  |
| Zhang | C | Med Sci Monit 2016 |  |  |  |  |  |  |  |  |  |
| Zhang | CS | BMC Musculosk Dis 2018 |  |  |  |  |  |  |  |  |  |
| Zhou | CS |  |  |  |  |  |  |  |  |  |  |
| Zhu | CS |  |  |  |  |  |  |  |  |  |  |
| Zietek | CS |  |  |  |  |  |  |  |  |  |  |
| Zou | C | Clin Chim Acta 2017 |  |  |  |  |  |  |  |  |  |
| Zou | C | Biofactors 2019 |  |  |  |  |  |  |  |  |  |

| **Case control studies (C)** | **Cohorts studies (Co)** | **Cross-sectional studies (CS)** |
| --- | --- | --- |
| **Q1.** Is the case definition adequate ? | **Q1.** Representativeness of the exposed | **Q1.** Is the representativeness of the cases adequate? |
| **Q2.** Is the representativeness of the cases adequate? | **Q2.** Selection of the non exposed | **Q2.** Is the sample size justified and satisfactory? |
| **Q3.** Selection of controls | **Q3.** Ascertainment of exposed | **Q3.** Is the non-response rate satisfactory? |
| **Q4.** Definition of controls | **Q4.** Outcome of interest was no present at start | **Q4.** Is the ascertainment of the screening/surveillance tool validated ? |
| **Q5.** Study controls for the most important factor | **Q5.** Study controls for the most important factor | **Q5.** Are the potential confounders investigated by subgroup analysis or multivariable analysis ? |
| **Q6.** Study controls for any important factor | **Q6.** Study controls for any important factor | **Q6.** Is assessment of the outcome adequate ? |
| **Q7.** Ascertainment of outcome | **Q7.** Assessment of outcome | **Q7.** Is the statistical test clearly described and appropriate ? |
| **Q8.** Same ascertainment method for cases  and controls | **Q8.** Follow-up long enough |  |
| **Q9.** Non response rate | **Q9.** Adequacy of follow up |  |

C = case control study; CS = cross-sectional study; Co = cohort study. = Yes = No = Can’t tell = Not concerned

Table S7. Meta-analysis of correlation coefficients for the most studied biomarkers (i.e., in at least three studies).

| Biomarkers | Fluid | N | n | Overall r expressed by ES | 95% CI |
| --- | --- | --- | --- | --- | --- |
| Adiponectin | Blood | 4 | 1,067 | -0.10 | -0.53, 0.33 |
| Adiponectin | SF | 4 | 496 | 0.05 | -0.12, 0.23 |
| BDNF | Blood | 3 | 82 | 0.07 | -0.36, 0.49 |
| CRP | Blood | 8 | 2,813 | 0.34 | 0.16, 0.52 |
| COMP | Blood | 8 | 926 | 0.26 | 0.01, 0.50 |
| CTX-I | Blood | 3 | 281 | -0.03 | -0.15, 0.08 |
| ESR | Blood | 4 | 874 | 0.29 | 0.04, 0.55 |
| HA | Blood | 7 | 881 | 0.10 | -0.01, 0.20 |
| hsCRP | Blood | 9 | 1,518 | 0.27 | 0.18, 0.36 |
| IL1 | Blood | 9 | 432 | -0.09 | -0.27, 0.08 |
| IL6 | Blood | 14 | 1,107 | 0.19 | 0.07, 0.30 |
| IL6 | SF | 10 | 791 | 0.01 | -0.09, 0.12 |
| IL8 | Blood | 4 | 215 | 0.12 | -0.13, 0.37 |
| IL8 | SF | 3 | 136 | -0.02 | -0.47, 0.43 |
| IL10 | Blood | 5 | 407 | 0.12 | 0.02, 0.22 |
| IL17 | Blood | 3 | 235 | 0.32 | 0.20, 0.44 |
| Leptin | Blood | 8 | 674 | 0.25 | 0.12, 0.28 |
| Leptin | SF | 6 | 813 | 0.09 | 0.02, 0.16 |
| MMP3 | SF | 3 | 317 | 0.04 | -0.40, 0.48 |
| MMP13 | SF | 3 | 316 | 0.32 | 0.02, 0.63 |
| PIIANP | Blood | 3 | 349 | 0.09 | -0.02, 0.19 |
| Resistin | Blood | 3 | 150 | 0.19 | 0.03, 0.35 |
| Resistin | SF | 3 | 395 | 0.14 | -0.11, 0.39 |
| TNF alpha | Blood | 11 | 549 | 0.15 | -0.02, 0.32 |
| TNF alpha | SF | 9 | 539 | 0.21 | -0.03, 0.45 |
| TNF alpha | CSF | 3 | 153 | 0.06 | -0.10, 0.22 |
| TNF alpha R1 | Blood | 4 | 152 | -0.13 | -0.57, 0.31 |
| TNF alpha R2 | Blood | 4 | 152 | 0.19 | -0.10, 0.47 |

N= number of studies; n= number of patients; r= correlation coefficient; ES= effect size; CI= confidence interval; SF= synovial fluid; CSF= cerebrospinal fluid

Figure S1. Forest plot of the correlation coefficients between pain and blood C reactive protein.

ES: effect size; CI: confidence interval

Figure S2. Forest plot of the correlation coefficients between pain and blood high sensitivity C Reactive protein.

ES: effect size; CI: confidence interval

Figure S3. Forest plot of the correlation coefficients between pain and blood interleukin 17.

ES: effect size; CI: confidence interval
